# Candidate Structure Prioritization in Non-Target Screening with Predicted Collision Cross-Sections and Uncertainty Quantification

**DOI:** 10.1021/acs.jcim.6c02492

**Published:** 2026-09-24

**Authors:** Lucas Ferrando Plo, Anneli Kruve

**Affiliations:** † Department of Chemistry, 7675 Stockholm University, 11418 Stockholm, Sweden; ‡ Department of Environmental Science, 7675 Stockholm University, 11418 Stockholm, Sweden

## Abstract

Candidate structure prioritization in non-target screening (NTS) can be performed using collision cross-sections (CCSs) predicted by a machine-learning (ML) model, with a threshold on the relative difference between measured and *in silico* CCSs. Such thresholds generally are heuristic and lack a statistical foundation. In this context, we have constructed a conformal predictive system to quantify the uncertainty of the ML-predicted CCSs and provide guarantees on the minimum percentage of prioritized true structures among the candidate lists of the NTS features detected in a sample. The system is designed as a wrapper around the open-source ML model GraphCCS and can, in principle, be combined with any CCS model. Additionally, we propose the detection of the applicability domain of the conformal predictive system based on maximum common-edge subgraph (MCES) molecular distances. We showcase the application of our tool to two independent *in silico* NTS datasets. Results on the two tested external datasets suggest that applicability domain detection based on the MCES distance allows to maintain the guarantees on the minimum percentage of true structures prioritized. In terms of candidate structure prioritization performance, on average ∼16% of incorrect candidates could be deprioritized while retaining 90% of all true structures. Although this constitutes a modest 8% improvement over random prioritization, our tool additionally offers adaptivity of removal efficiency to the difficulty of the NTS candidates based on quantified uncertainty. Furthermore, the framework can potentially be combined with other analytical properties to improve candidate structure prioritization in NTS.

## Graphical abstract

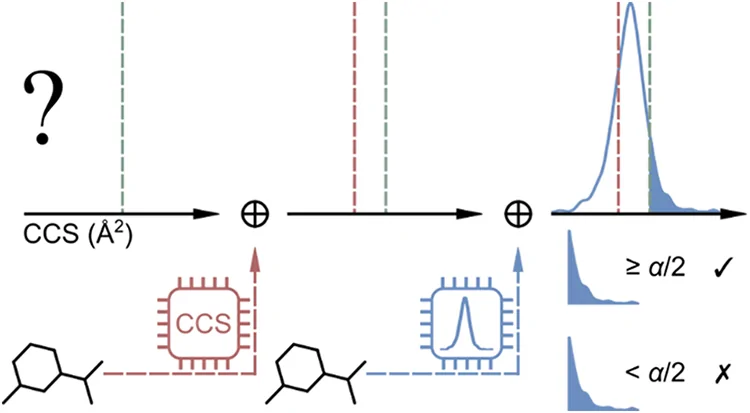

## Introduction

Gaining insights into the structure of unknown small molecules is one of the main challenges in metabolomics, lipidomics, exposomics, and environmental monitoring alike. The collision cross-section (CCS) represents a measure of size and shape of gas-phase ions determined by ion mobility spectrometry (IMS), under defined experimental conditions.^1,2^ IMS has emerged as a powerful technique for obtaining structural information^3^ due to its rapid separation time scale,^4,5^ enabling its incorporation into mass spectrometers (MS) while maintaining separation in additional hyphenated dimensions, such as chromatography.^6^ In particular, IMS has been recognized in structural characterization of small molecules due to the separation mechanisms complementary to chromatography and as an additional source of empirical analytical information for substance identification.^2,7^ Experimental CCS values have been shown to be highly reproducible and, thus, are a promising quantity to aid structure elucidation.^7^

In non-target screening (NTS), a common procedure is to prioritize candidate structures for an unknown feature based on empirical analytical information.^8^ In that regard, CCSs can be leveraged in structural annotation if reference values are either available or can be predicted. While extensive work to generate high-quality comprehensive reference libraries for database searching has been undertaken,^9,10^ it is limited to already measured chemicals. Thus, modeling of CCSs both from first-principles^11^ and through machine learning (ML)^12–19^ has gained attention in recent years. Candidate structure prioritization can then be performed by comparing the predicted CCS for a given candidate structure with the measured CCS of the corresponding NTS feature. An agreement of these values is defined by a threshold on the relative difference between the *in silico* and measured CCS values.^8^ This approach has been shown to reduce the number of candidate structures,^16,18,20–22^ although the removal efficiency greatly depends on the specific NTS dataset at hand. However, relative thresholds generally lack statistical foundation, as they are usually heuristic.

In this context, a validated (that is, calibrated) uncertainty quantification^23^ (UQ) approach is essential in assessing the confidence in the predicted CCS values and, thus, for evaluating the probability of the candidate structure. Additionally, these uncertainty estimates must be sufficiently tight to allow enough discrimination between candidate structures. This requirement is particularly stringent in CCS prediction, where accuracy <1% has been deemed necessary due to the small differences between isomeric species.^7^ Akhlaqi et al.^24,24^ indicated that CCS models need to make estimation errors of 0.15% at most in order to eliminate 95% of the candidate structures. In that regard, de Cripan et al.^14^ showed that widely used CCS prediction tools do not meet this requirement. In addition to well-calibrated and tight uncertainty estimates, defining the border conditions for applicability of the CCS prediction tool—distinguishing out-of-domain (OOD) and in-domain candidate structures—is of utmost importance.^25^ Otherwise, applying the CCS model for OOD candidate structures would potentially lead to the miscalibration of the uncertainty estimates and the loss of the desired accuracy.^26^ Although several existing CCS prediction tools implement some form of prediction confidence,^12,20,27^ there remains a lack of a system that accounts for the interplay between OOD detection and well-calibrated, statistically sound uncertainty estimates.

In this work, we address the urgent need for UQ and OOD detection in CCS-based candidate structure prioritization in NTS. In particular, an approach to estimate the uncertainty of CCSs predicted by available CCS ML models is presented. UQ is based on conformal predictive systems that provide theoretical guarantees on the minimum percentage of true structures retained after filtering among the candidate lists of the NTS features detected in a sample.^28^ We also propose a detection for the applicability domain of the system based on maximum common-edge subgraph (MCES) molecular distances. Results on two independent *in silico* NTS datasets suggest that conformal predictive systems are able to accompany ML-based CCS predictions with calibrated uncertainty estimates and that the OOD detection system is capable of maintaining the aforementioned theoretical guarantees. For example, we observed that ∼16% of incorrect candidates can be deprioritized while retaining 90% of all true structures present in the candidate lists, which constitutes an 8% improvement over random prioritization. The results show that, while CCSs can be used to filter some candidates, they currently need to be combined with other analytical properties for improving prioritization performance.

## Data

### METLIN-CCS: Model Fitting and UQ Calibration

The CCS library METLIN-CCS from Baker et al.^9^ is the largest experimental CCS dataset to date, comprising a wide range of small organic molecules that span a broad chemical space. The dataset was cleaned and standardized. Firstly, SMILES strings^29^ were manually checked for large disconnected structures and intrinsically charged analytes, which can affect the standardization process. Secondly, the SMILES were standardized with RDKit,^30^ while dimers were not considered in the further processing. Lastly, duplicates were checked for but none were found. Altogether, 13 entries showed mismatch of empirical and theoretical information (*m/z*, ion type, *etc*.) or corresponded to large disconnected compounds. These were ultimately retained, as these filtering steps were not considered in previous publications using METLIN-CCS,^14,27^ and removing them here would compromise comparability; however, it may be appropriate to take this into account for future work. The final dataset contains 27,377 SMILES comprising 58,041 ion species, of which 24,830 are [M + H]^+^, 15,048 [M + Na]^+^ and 18,163 [M – H]^−^. Ion species indicates in this work the combination of the ion type and a specific chemical structure (SMILES).

METLIN-CCS was split into a final training and calibration sets in a 9:1 ratio, seeded for reproducibility. This relation was selected because, on the one hand, it leaves a sufficiently large number of training examples to facilitate CCS model fitting and, on the other hand, the number of calibrants allows to guarantee a stable UQ calibration. The final training set was also split to perform seeded random 5 × 5 cross-validation, as suggested by Ash et al.,^31^ and each cross-validation training fold further divided into training and calibration splits in the same 9:1 ratio. The 25 folds were used for hyperparameter optimization of the conformal predictive system for each CCS ML model, and subsequently for the statistical comparison of the best-performing configurations to select a final UQ setup. This differs from the nested hyperparameter-selection procedure proposed by Ash et al.,^31^ in which the configuration of the conformal predictive system may vary across outer folds. Our objective instead was to identify a single, fixed configuration for each CCS model and compare the resulting conformal predictive systems. Accordingly, the complete 5 × 5 cross-validation results were used for both configuration selection and statistical comparison, prioritizing a larger effective sample size for stable calibration while acknowledging the resulting post-selection dependence. Nevertheless, the magnitude of this bias is expected to be limited by applying the same hyperparameter search and selection procedure across all models.

Additionally, 25% of all unique chemicals in each training set were subsampled as “training background”, against which to compute the MCES molecular distances and, thus, reduce the number of MCES calculations. To check that the splits corresponded to the same chemical subspace, box plots of the CCS values, as suggested by Ash et al.,^31^ and myopic MCES distances^32^ with myopic threshold set at 10 were compared across samples (Supporting Information, SI—Split Distributions and SI—Subsampling Distributions).

### EnvCCS & AllCCS: Performance Tests at the Limits of the Applicability Domain

#### EnvCCS

The reference library published by Teri et al.,^10^ here called EnvCCS, includes CCS values for a broad chemical space of environmentally relevant compounds, a domain where NTS is frequently employed.^8^ Moreover, its measurement bias relative to METLIN-CCS can be neglected, as EnvCCS was shown to have inter-lab reproducibility ≤2%.^9,10^ In that regard, relative deviations between all common entries in METLIN-CCS and EnvCCS were found to be <1%, although based only on six instances. The same aforementioned standardization steps for SMILES were followed here. Additionally, only ion types present in METLIN-CCS and ion species not contained in METLIN-CCS were retained. Duplicated entries were merged when the relative difference was ≤1% and removed otherwise. Entries for ion species with higher number of replicates were manually checked. Furthermore, for [M – H]^−^ the presence of hydrogens in the molecular formula was checked and one entry was removed. The final dataset contains 1922 SMILES and 2,888 ion species, of which 921 are [M + H]^+^, 868 [M + Na]^+^ and 1099 [M – H]^−^.

#### AllCCS

The compiled library AllCCS curated by Zhang et al.^12^ was selected as an additional external test dataset complementary to EnvCCS, as it allows to select entries with specific experimental measurement confidence levels. In this study, only the two highest quality classes were considered and the previous described standardization steps for SMILES were applied. Additionally, only ion types present in METLIN-CCS and ion species contained neither in METLIN-CCS nor in EnvCCS were retained. This requirement for a disjoint dataset resulted in the removal of one common entry with METLIN-CCS, with CCS deviation of 1.5%, and 178 common CCS values with EnvCCS, with mean CCS deviation of 0.75%. Same considerations for replicates as for EnvCCS were also applied here. The final dataset contains 990 ion species corresponding to 677 SMILES, of which 419 are [M + H]^+^, 301 [M + Na]^+^ and 270 [M – H]^−^.

### EnvCCS_NTS & AllCCS_NTS: Candidate Structure Prioritization

To evaluate the CCS UQ system for candidate structure prioritization in NTS, one would commonly conduct a spiking experiment with standards. Instead, we leveraged EnvCCS and AllCCS as sources of true structures to compile two independent *in silico* NTS datasets, which enables evaluation on a larger pool of candidate and true structures.

#### High-Resolution MS Data

As candidate structure retrieval in NTS is based on high-resolution MS data, the true structures obtained from the two CCS libraries were complemented with MS^1^ and MS^2^ spectra from public libraries. MassBank of North America, MassBank,^33^ GNPS,^34^ NIST23 ^35^ as well as Wageningen’s Food Safety Mass Spectral Library^36^ were downloaded on the 25th of November 2025 and MS^2^ spectra from liquid chromatography coupled to Orbitrap^37^ instruments were retrieved for compounds with experimental CCS values from EnvCCS and AllCCS. Matching was performed considering the ion type and the first 14 characters of the InChIKey string (InChIKey2D), to account for the fact that MS generally only provides information on connectivity.^38^ When several CCS values for the same ion species based on the InChIKey2D existed, one CCS was randomly picked, seeded for deterministic results. For each ion species, only one parent mass corresponding to the isotopologue with most recorded MS^2^ spectra was selected. Spectra were found for 1661 and 700 ion species of EnvCCS and AllCCS, respectively. MS^1^ isotope patterns were then simulated using IsoSpec^39^ with an intensity threshold of 0.1%. Finally, MS data was distorted at both the MS^1^ and the MS^2^ levels to approximate a more realistic data retrieval. For the former, gaussian noise was added to the intensities^40^ and *m/z* values were shifted according to the difference between the experimental and the theoretical parent masses of the parent ion of the MS^2^. For the latter, one MS^2^ corresponding to a medium collision energy was randomly selected among those available. The results with undistorted MS data are provided in SI—NTS Prioritization with Raw MS Data.

#### Candidate Structure Retrieval

SIRIUS + CSI:FingerID^40^ v. 6.3.3 with default bottom-up settings was applied to retrieve the top 10 candidate structures for each *in silico* NTS feature and SMILES were standardized as described above. Candidate structures were suggested for 1431 and 587 MS spectra of ion species of EnvCCS and AllCCS, respectively, of which 1263 (88%) and 538 (92%) contained the correct structure among the top 10 candidates. The true structure was annotated as the top 1 match in 87% of the ion species and the molecular formula was correct for 87% of the candidate structures, in both datasets. Note that the elucidation performance was higher compared to that observed on data from real sample measurements in NTS.^41^ Consequently, the candidate structure prioritization task for the two *in silico* datasets in this study is comparatively more challenging than in NTS, as structures similar to the correct one are expectedly harder to distinguish from the true structure. Thus, the results presented in this work can be considered conservative. Note that we aim here to isolate and evaluate the CCS-based candidate structure prioritization step alone. True NTS data for assessing the full NTS workflow is much more heterogeneous than what we are using in this study, including wrong formulas, missing candidates, noisier spectra, adduct ambiguity, *etc*. In total, 12,464 and 5274 candidate structures were obtained for EnvCCS and AllCCS, respectively; 11,227 and 4900 counting only those from NTS features for which the correct structure was suggested among the top 10 candidates. Only those 11,227 and 4900 were considered for the final two *in silico* NTS datasets, EnvCCS_NTS and AllCCS_NTS. The number of CCS values for [M + H]^+^, [M + Na]^+^ and [M – H]^−^ in EnvCCS_NTS are 609, 155 and 499, respectively, and 305, 73, and 160 in AllCCS_NTS.

## Methods

### CCS ML Approaches

The best-performing existing ML models for CCS point estimation were selected from the literature based on benchmarking studies by de Cripan et al.^14^ and Hart et al.^27^ These models include:

- GraphCCS,^18^ a deep graph neural network that operates on ion graphs using Gasteiger charges;^42^
- CCSP2.0,^16^ which employs ion-type-specific support vector regressors^43^ that use Mordred molecular descriptors^44^ as input;
- SigmaCCS,^17^ a graph neural network that incorporates 3D conformers as input;
- KerasECFP,^14^ a feed-forward neural network trained on extended connectivity fingerprints (ECFPs) of radius two, ECFP4s, and
- a linear regression model on *m/z* as baseline.

Hyperparameters were retained as reported in the respective publications without further tuning. However, all models were monitored for overfitting, which led to the adjustment of GraphCCS training’s weight decay to 0.01.

### Conformal Predictive Systems

Among the UQ approaches available,^23^ we selected conformal predictive systems,^28^ as they allow to turn a CCS point estimate from a CCS ML regressor into a predictive distribution, without modifying the underlying CCS prediction model. More importantly, the theoretical framework of conformal predictive systems specifically provides statistical guarantees on the minimum percentage of prioritized true structures among the candidate lists of the NTS features detected in a sample.^28^ Briefly, the algorithm to build conformal predictive systems^28,45^ for CCS ML regressors includes:

1. The above-described CCS ML point-estimation models, yielding $\hat{CCS}$ , are fitted on the METLIN-CCS training sets.
2. A function that estimates the absolute error, $\hat{|e|}$ , that the CCS model would make for an ion species, is fitted on the same METLIN-CCS training sets.
3. On each METLIN-CCS calibration set, disjoint from the above-mentioned training sets of their corresponding split, the CCS score for each calibrant *c*, *s*_*c*_^CCS^, is calculated:
1 scCCS=ec|e|^c
  The conformal predictive system is then said to be normalized by the absolute error estimates. After that, all *s*_*c*_^CCS^ are sorted in ascending order, prepending and appending –*∞* and *∞* to the list of *s*_*c*_^CCS^.
4. To obtain a predictive distribution for the $\hat{CCS}$ of a new ion species, each *s*_*c*_^CCS^ is transformed to produce *s*_*c*_^′CCS^
2 sc′CCS=scCCS·|e|^+CCS^
  where $\hat{|e|}$ and $\hat{CCS}$ denote the estimates of the absolute error and the CCS, respectively, of the new ion species. The predictive distribution is represented as the list of all *s*_*c*_^′CCS^.

A Mondrian construction of a conformal predictive system modifies the above procedure by dividing the calibrants into non-overlapping classes, called taxonomies, here denoted as κ. A normalized conformal predictive system is fitted within each taxonomy following the above-described algorithm, resulting in within-taxonomy calibration as long as the classes were found independently of the calibrants. Two approaches are considered here to potentially set the taxonomies: discretization of training $\hat{CCS}$ with heteroscedasticity^45^ or ion types, as there are literature reports indicating different CCS predictive performance across ion types.^14,16,18^

#### Error Function

In this work, $\hat{|e|}$ for a given instance is based on the arithmetic mean of MCES distances to the *k* nearest compounds in the METLIN-CCS training sets, denoted as ⟨MCES⟩_*k*_. Specifically, the error function is defined as *a*⟨MCES⟩_*k*_^*n*^, where *n* and *k* are determined through METLIN-CCS cross-validation and *a* is left undefined, as the calculation of *s*′^CCS^ is independent of it. The prior assumption is that absolute errors follow a monotonically increasing function from (*a*⟨MCES⟩_*k*_^*n*^ = 0, |*e*| = 0). On the other hand, *a*⟨MCES⟩_*k*_^*n*^ was fitted globally and is taxonomy-independent. We also performed additional experiments to evaluate alternative approaches to build an error function based on:

- ⟨MCES⟩_*k*_ with normalized MCES distances,^46^
- ensemble variance from METLIN-CCS cross-validation^47^ or a CCS random forest^48^ regressor on ECFP4s,^49^ and
- a separate error model. Experiments consisted in this case on a feed-forward neural network, random forest or support vector regressors on the embeddings of a CCS graph neural network;^50^ a feed-forward neural network, support vector or random forest regressors on ECFP4s with concatenated one-hot-encoded ion type; and a random forest regressor on a feature space composed of *m/z*, METLIN-CCS cross-validation ensemble standard deviation, $\hat{CCS}$ and one-hot-encoded ion type.^51–53^ Additionally, we implemented the potential support vector regressor^54^ with the maximum common-subgraph kernel as Mohr et al.^55^ To do so, we calculated absolute and normalized MCES distances between ion graphs, generated using the approach proposed by Xie et al.^18^ based on Gasteiger charges.^42^

#### Candidate Structure Prioritization in NTS

For prioritization of a given candidate structure, one can obtain the probability for the measured CCS, $P^{CCS}$ , of the corresponding NTS feature to originate from the candidate’s predictive distribution:

3 $$P^{CCS}=2\min\left(p,100-p\right)$$

where *p* is calculated as

4 $$p=100\frac{c+\tau }{n_{cal}^{CCS}+1}$$

with *c* as the number of finite *s*′^CCS^ in the candidate’s distribution that are lower than or equal to the CCS, *n*_cal_^CCS^ denotes the total number of finite CCS calibrants in the candidate’s distribution and τ is a random number sampled from the uniform distribution, τ ∼ *U*(0,1), needed to break ties. The term 2min deviates from the equation given by Vovk et al.^28^ to calculate a two-sided probability value.

Candidate structures can be prioritized if:

5 $$P^{CCS}\geq \alpha$$

where α ∈ [0%, 100%]. The statistical framework of conformal predictive systems then theoretically guarantees that the percentage of true structures retained after filtering among the candidate lists of the NTS features detected in a sample will stay ≥100 – α (Figure 1).^28^

**Figure 1:**
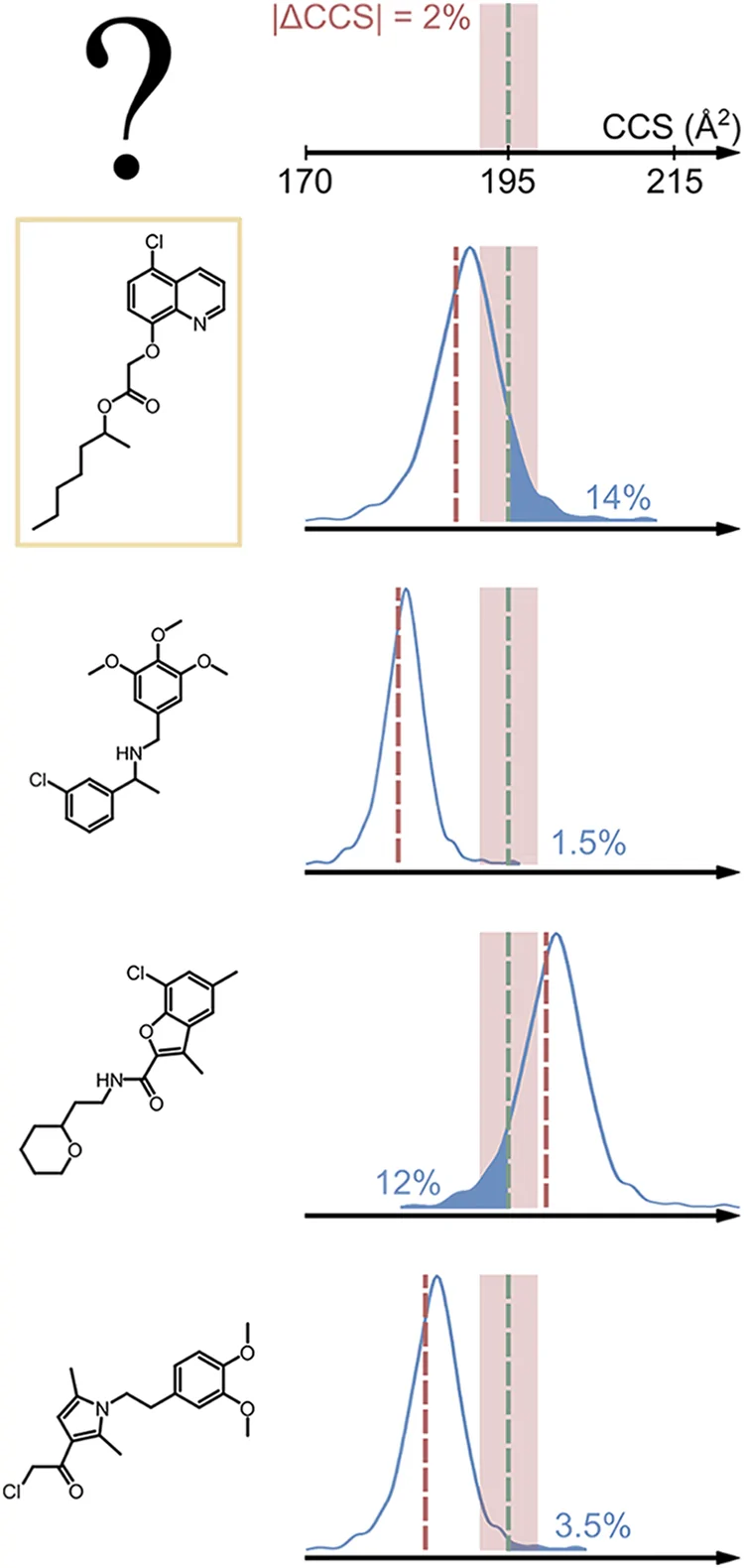
Schematic depiction of candidate structure prioritization in NTS for one unknown (green) with 4 candidates (the true structure is highlighted with a yellow frame). Strategies based on point estimates (red) check if the predicted CCS of each candidate structure shows a deviation relative to the measured CCS higher than a selected cut-off value, *e.g.*, 2%. Strategies based on predictive distributions (blue) evaluate the corresponding tail area that falls either under or above the measured CCS. If the doubled shaded region of a candidate structure is smaller than a selected threshold, α, the chemical is deprioritized. The statistical framework of conformal predictive systems theoretically guarantees that the true structure will be retained in 100 – α (%) of the cases across all NTS features in the NTS dataset. Note that the point estimate does not necessarily need to coincide with the modes of the predictive distributions, as shown on this figure.

On the other hand, continuous ranked probability scores (CRPSs, Å^2^) can be implemented for candidate structure ranking. It quantifies the agreement between the CCS predictive distribution of a candidate structure and the measured CCS of the NTS feature. It considers both the proximity of the distribution to the measured CCS and its tightness around it. The formal definition is given in eq (S1). A CRPS of 0 corresponds to a perfect prediction and the score does not have an upper bound.^28^ However, all candidate structures for an NTS feature must lie within the applicability domain of the system to implement the CRPS as a valid ranking rule.

#### Calibration-Conditional Validity

The chosen calibration set of a final conformal predictive system may be an unrepresentative combination of calibrants. Consequently, the probabilities $P^{CCS}$ generated with eq ([Disp-formula eq3]) may not be generally valid. One possible solution is to condition $P^{CCS}$ on the used calibrants, that is, making $P^{CCS}$ calibration-conditionally valid, here denoted as $P_{\mathrm{ccv}}^{\mathrm{CCS}}$ . To do so, Bates et al.^56^ proposed an empirical approach based on simulating several calibration sets, the total number of which is set to 10,000 in this work. A correction function, *h*, can then be found such that $P_{ccv}^{CCS}=h\left(P^{CCS}\right)$ , by searching for an envelope function that encloses all $P^{CCS}$ generated across all simulated datasets in 100­(1 – δ)% of cases. δ ∈ [0,1] could also be understood as the parameter controlling the confidence of the correction, and in this work δ = 0.05 was used as default. Note that selecting a very high confidence, that is, very low δ, will result in a conformal predictive system with limited discriminative power and only few incorrect candidate structures would be deprioritized. For additional details on *h*, refer to eqs (S2–S5) in the SI or to the work of Bates et al.^56^

### Evaluation Measures

#### Point Estimation

Two point-estimation regression performance metrics, as recommended by Ash et al.,^31^ are considered, specifically the arithmetic mean of *R* ^2^ scores (a.u.) and 90th percentile of percentage absolute errors relative to the true CCSs across the METLIN-CCS 5 × 5 cross-validation folds. Additional performance metrics are presented in the SI.

#### CRPSs

Aside from candidate structure ranking, CRPSs can be applied to assist selection of conformal predictive systems, favoring configurations that yield lower values of this performance metric.^57^ Alternative configurations of conformal predictive systems were compared based on the arithmetic mean of the CRPSs, with stratification to down weight densely populated regions. Specifically, the validation ion species in each METLIN-CCS 5 × 5 cross-validation fold were partitioned based on the continuous variable ⟨MCES⟩_*k*_ into equal-width categories. The binning was designed to yield the maximum number of classes containing at least 25 ion species, resulting in ∼6 bins. This minimum threshold was selected to maximize the number of categories, thereby achieving optimum coverage of the ⟨MCES⟩_*k*_ dimension, while still retaining a sufficient number of ion species per bin to ensure a representative estimate of the arithmetic mean of CRPSs per class. To prevent the distortion of the category boundaries, the 0.5% upper most extreme ion species in terms of ⟨MCES⟩_*k*_ were excluded prior to fitting the partition. Subsequently, the resulting bin edges of the classes were applied to the full validation fold to discretize the continuous ⟨MCES⟩_*k*_ variable into categories. Finally, an overall arithmetic mean across the bin-specific estimates was calculated to obtain ⟨CRPS⟩, thereby assigning equal weight to each ⟨MCES⟩_*k*_ interval irrespective of the number of ion species contained in each bin.

The ⟨CRPS⟩ performance metric across the METLIN-CCS 25 5 × 5 cross-validation folds was statistically compared between conformal predictive systems *via* a repeated measures analysis of variance (ANOVA) with a Tukey’s honestly significant difference *post-hoc* test (HSD), as suggested by Ash et al.^31^ The independence assumption of ANOVA + Tukey’s HSD was approximated by the 5 × 5 cross-validation sampling scheme, whereas the homogeneity of ⟨CRPS⟩ variances across conformal predictive systems was checked with the Levene’s test and with fold differences. Lastly, normality of ⟨CRPS⟩ across the METLIN-CCS 25 5 × 5 cross-validation folds was qualitatively evaluated with quantile-quantile plots and histograms.

#### Recall

Empirical recall is defined as the percentage of true structures that are retained after candidate structure prioritization based on eq ([Disp-formula eq5]). For the datasets EnvCCS and AllCCS, which do not include candidate structures, this can be interpreted similarly: it represents the percentage of ion species that would be prioritized with eq ([Disp-formula eq5]) if each ion species were treated as its own candidate structure, using its database CCS value as the measured CCS of the corresponding NTS feature.

Although a conformal predictive system is designed to theoretically guarantee that empirical recall remains at least 100 – α, it may fluctuate around this target due to the randomness of any dataset. When reporting results for EnvCCS and AllCCS, we approximate the distribution under target recall with a binomial distribution, $\frac{100}{n_{test}}\mathrm{Binom}\left(n_{test},1-\frac{\alpha }{100}\right)$ , where *n*_test_ denotes the number of test ion species, by considering recall as a series of independent Bernoulli trials, each with success probability of $1-\frac{\alpha }{100}$ .^58^ We give the 5th binomial lower-tail percentile as a lower bound, so that empirical recall is considered to fulfill the statistical guarantee as long as it is higher than or equal to the given binomial percentile, even if it does not exactly match the target recall. As the recall guarantees in a Mondrian conformal predictive system apply across all test ion species as well as within each individual taxonomy, we also evaluate the minimum empirical recall across the Mondrian classes. In this case, the *n*_test_ parameter used in the binomial distribution refers only to the number of test ion species within the taxonomy that shows the lowest empirical recall. We also perform the analogous analysis on the ⟨MCES⟩_*k*_ variable. Note that there are no theoretical statistical guarantees in this case.^58^ To do so, the ⟨MCES⟩_*k*_ dimension was partitioned using quantile discretization into categories of 150 instances. The bin size was chosen to balance two competing objectives: minimizing the number of ion species per class to maximize the total number of categories and enable a fine-grained analysis of conditional empirical recall, while maximizing the class size to ensure a sufficiently tight binomial distribution under target recall per category. In this trade-off, priority was given to the former. Subsequently, empirical recall was calculated within each bin and the minimum empirical recall across all classes was reported.

#### Efficiency

An empirical recall exceeding the lower bound given by the binomial percentile alone is insufficient for assessing the applicability of a conformal predictive system in the context of candidate structure prioritization for NTS. For instance, trivially wide CCS predictive distributions could most likely meet this criterion, yet such a system would be of little practical use: its weak discriminative power would result in minimal deprioritization of incorrect candidate structures. Thus, we additionally report the percentage of incorrect candidate structures in EnvCCS_NTS and AllCCS_NTS that are deprioritized, *i.e.*, efficiency, which should ideally be close to 100%. Consequently, a conformal predictive system that fulfills its recall guarantees only by producing wide predictive distributions will exhibit low efficiency, indicating limited practical utility. We provide a lower bound for efficiency based on random prioritization, defined as $\frac{f}{1-\pi }\left(100-r\right)$ , where *r* denotes empirical recall, *f* is the fraction of incorrect candidate structures estimated in-domain and π is the fraction of true structures estimated OOD.

### Applicability Domain

The aforementioned theoretical statistical guarantees provided by a conformal predictive system are valid only for NTS samples where candidate structures fall within its applicability domain. The work of Bates et al.^56^ on conformal prediction for outlier detection was taken as inspiration for OOD detection to flag candidate structures outside the domain of applicability. The algorithm can be concisely described as follows:

1. Fit on each METLIN-CCS training set an OOD scorer that produces OOD scores, *s* ^OOD^, so that a higher *s* ^OOD^ may suggest that the corresponding ion species is further away from the applicability domain.
2. Calculate *s* ^OOD^ for all data points in each METLIN-CCS calibration set and sort all *s* ^OOD^ in ascending order. The calibration set should be disjoint from the training set to avoid data leakage and ensure that *s* ^OOD^ stays unbiased for previously unseen ion species.
3. For a new ion species, calculate its *s* ^OOD^. We give the percentage of calibrants with an *s* ^OOD^ that is lower than or equal to the new ion species’ *s* ^OOD^ as a rank-based measure of the OOD degree for the new ion species, denoted as P^OOD^.

We base the OOD scorer on the error function of the conformal predictive system, as an *s* ^OOD^ resulting in higher evidence that a candidate structure is OOD should correlate with higher absolute errors. Specifically, we set *s* ^OOD^ = ⟨MCES⟩_*k*_. Due to the METLIN-CCS splitting at the ion species level applied in this study, the same neutral compound can potentially appear in both the training and calibration sets. This contradicts the above-described algorithm, as the splitting must be disjoint at the neutral species level for ⟨MCES⟩_*k*_ to be unbiased. To mitigate this effect, self-distances in the calibration sets were set to ∞. In any case, MCES distances from the ion graphs generated with Gasteiger charges,^42^ as suggested by Xie et al.,^18^ could strictly solve this issue, albeit of a higher computational cost. On another note, the terms “chemical space” and “applicability domain” will be used interchangeably, since the OOD scores are derived from molecular distances between new chemicals and those in the training set.

As the main purpose of implementing the conformal predictive system is ultimately retaining the target recall in an NTS sample, we opted to evaluate the OOD detection system based on empirical recall, although several approaches exist to assess the performance of an OOD scorer.^50,59,60^ To do so, we first empirically derive a practical OOD threshold based on P^OOD^ by progressively filtering ion species in the larger EnvCCS dataset according to their P^OOD^ until the lower recall bound given by the binomial percentile was surpassed. Specifically, only ions with P^OOD^ below or equal to values in the range of 40% to 100%, the latter corresponding to no filtering, in steps of 1% were retained and considered to calculate empirical recall. Due to the reduced number of ion species in EnvCCS at lower thresholds, 40% was selected as the minimum. On the other hand, α levels 10%, 5% and 1% (eq 5) were evaluated, as this confidence range was considered to be most important for applications of conformal predictive system in NTS. This P^OOD^ threshold found on EnvCCS was then applied to AllCCS and empirical recall was compared against the lower recall binomial bound across all α ∈ [0%, 100%]. The same *α* analysis for EnvCCS can be found in Figure S29.

The above-described OOD detection based on ⟨MCES⟩_*k*_ operates at the level of each individual ion species. Nevertheless, conformal predictive systems require, for the previously explained guarantees to hold, all ion species in an external dataset to be distributed across the domain of applicability in the same way as the calibrants. In NTS, all true structures may exhibit a very low ⟨MCES⟩_*k*_, suggesting that each may lie within the domain of applicability, yet all true structures could be concentrated in a particular region of the calibrated chemical space, which would violate the requirement. In practice, this requisite cannot usually be satisfied. We instead aim to quantify the distributional shifts between the calibration and the external test datasets, and, thus, empirically assess the practical robustness of the conformal predictive system to variations in the test chemical space. To characterize this divergence, we examine the training molecules for OOD detection selected as nearest neighbors in the computation of ⟨MCES⟩_*k*_ for the ion species in EnvCCS or AllCCS. Specifically, we calculate for each training chemical for OOD detection, *i*, the number of estimated in-domain test ion species for which *i* was selected as nearest neighbor. These counts are then turned into proportions and normalized so that their sum across all training chemicals for OOD detection equals to one

6 $$t_{i}=\frac{\sum_{j=1}^{n_{test}^{ID}}1_{i\in N\left(j\right)}}{k\cdot n_{test}^{ID}}$$

where *j* denotes a chemical among the total number of estimated in-domain test ion-species, *n*_test_^ID^, *N*(*j*) indicates the set of nearest neighbors for test chemical *j* and **1** is the indicator function. The empirical discrete distribution for the test chemical space is then *T* = (*t*_1_,···,*t*_*n*_train_^OOD^_), with *n*_train_^OOD^ as the number of training chemicals for OOD detection. The same procedure is applied to the CCS calibrants

7 $$t_{i}^{\prime }=\frac{\sum_{c=1}^{n_{cal}^{CCS}}1_{i\in N\left(c\right)}}{k\cdot n_{cal}^{CCS}}$$

where *N*(*c*) indicates the set of nearest neighbors for CCS calibrant *c*. The resulting empirical discrete distribution for the CCS calibration chemical space is then $C=\left(t_{1}^{\prime },\cdot \cdot \cdot ,t_{n_{train}^{OOD}}^{\prime }\right)$ . *T* and *C* are then compared using the log_2_ Jensen-Shannon (JS) distance, where a value of 0% indicates complete agreement between the calibrated and the test chemical spaces, and higher values, up to 100%, indicate increasing divergence.

## Results and Discussion

### Comparison of CCS Prediction Models with UQ

A 5x5 cross-validation was performed to choose the final conformal predictive system capable of producing the tightest predictive distributions centered closest to the true CCS values among literature-reported approaches. A sequential strategy was adopted to configure the conformal predictive system for each CCS point-estimation model.

Firstly, the accuracy of CCS predictions was evaluated to enable direct comparison with previously published benchmarking studies. All models exhibited similar performance consistent with reported literature values (SI—METLIN-CCS 5 × 5 Cross-Validation—Point-Estimation Performance). Overall, all models achieved an *R* ^2^ ≥ 0.88 and relative prediction error ≤4.5% in 90% of the cases. These results confirm the limited capability of current CCS prediction models to attain the <1% accuracy previously deemed a necessity for NTS.^7^

Secondly, the suitability of different approaches for constructing Mondrian taxonomies was examined. Point estimates were found to be inadequate for defining κ, as the prediction errors are independent of the point estimates (SI—METLIN-CCS 5 × 5 Cross-Validation—Mondrian Taxonomies—Point Estimates). In contrast, systematic differences in prediction accuracy were observed across ion types (Figure 2A and SI—METLIN-CCS 5 × 5 Cross-Validation—Mondrian Taxonomies—Ion Types). Protonated species exhibit tighter error distributions, while sodium adducts and deprotonated molecules showed distinct and broader error profiles. Such difference might arise from the lower number of [M + Na]^+^ and [M – H]^−^ training examples. Thus, the ion type was selected as the basis for Mondrian partitioning. As a consequence thereof, our tool is strictly applicable only to the ion types present in METLIN-CCS, that is [M + H]^+^, [M + Na]^+^ and [M – H]^−^. In the final calibration, ∼1500 calibrants are present in each of the three taxonomies.

**Figure 2:**
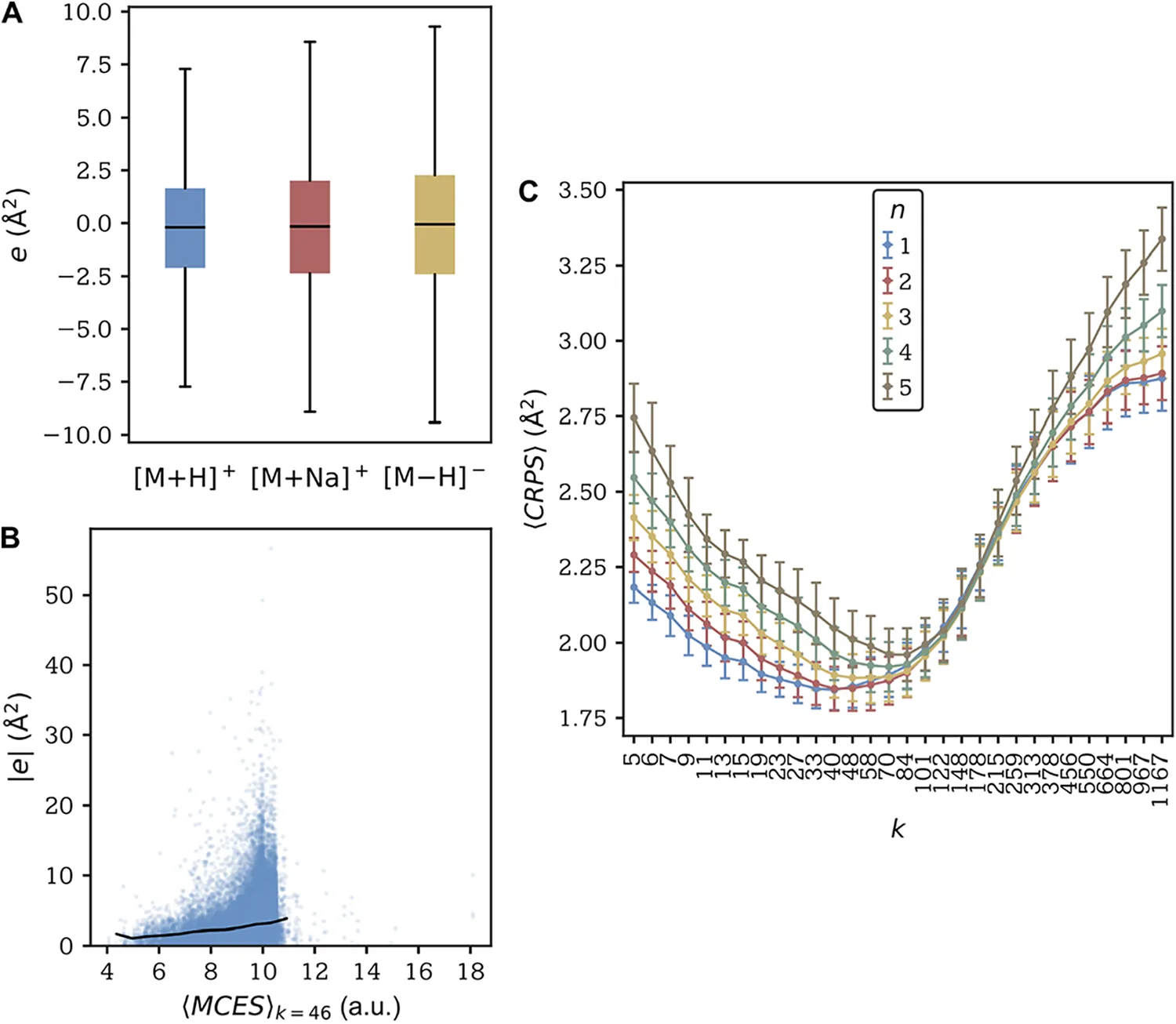
Characteristics of the conformal predictive system based on GraphCCS point-estimation model. (A) Error boxplots depending on the ion type for the final CCS model on the final METLIN-CCS training set. (B) Correlation plot between absolute errors on the final METLIN-CCS training set and ⟨MCES⟩_*k*=46_, together with the mean absolute error across the ⟨MCES⟩_*k*=46_ dimension (black line). C: mean and standard deviation of ⟨CRPS⟩ across the METLIN-CCS 25 5 × 5 cross-validation folds depending on *k* and *n*, as in *a*⟨MCES⟩_*k*_^*n*^,. To obtain the conditional mean in B, the same approach was applied as for the equal-width discretization of ⟨MCES⟩_*k*=46_ to calculate ⟨CRPS⟩ explained in Methods—Evaluation Measures—CRPS. In this case, the minimum sample count per bin was set at 12 to allow for detailed coverage of the ⟨MCES⟩_*k*=46_ dimension with ∼12 classes, albeit of slightly noisier estimates.

Thirdly, the hyperparameters *n* and *k* of the error functions were tuned to minimize ⟨CRPS⟩ (Figures 2C and SI—METLIN-CCS 5 × 5 Cross-Validation—Error Function—*n* & *k*). A characteristic U-shaped dependence of ⟨CRPS⟩ on *k* was observed. At larger *k* values, the computation of ⟨MCES⟩_*k*_ increasingly relies on approximate myopic MCES distances, thereby introducing additional approximation error. Conversely, at small *k* values, the metric loses discriminative power, as a small number of structurally similar compounds can often be found for a new molecule. The optimal *k*, therefore, lies between these two extremes, balancing discriminability against the proportion of approximate distance calculations. Regarding the exponent, *n* = 1 or *n* = 2 were generally preferred. This selection can be analyzed through the mean *|e|* depending on ⟨MCES⟩_*k*_, which reveals an approximately linear increase, in case of GraphCCS with slope 0.40 Å^2^/a.u. (Figure 2B), thereby explaining the selection of low-order exponents. On the other hand, ⟨MCES⟩_*k*_ was found to be a weak predictor of *|e|* but a more informative descriptor of absolute error dispersion. This observation suggests that ⟨MCES⟩_*k*_ could serve as basis for constructing Mondrian taxonomies. In that case, candidate structures associated with a given NTS feature would likely be assigned to the same partition due to their structural similarities,^61^ implying that differences in CCS predictive distributions would strictly be driven by the point-estimation model. Thus, this strategy for building Mondrian taxonomies was not investigated further. All the additional error function approaches were preliminarily evaluated and results can be found in the GitHub SI. None were able to yield a correlation with *|e|* stronger than ⟨MCES⟩_*k*_, as assessed by visual inspection, and were thus not optimized further.

Finally, the performances of each CCS model with their respective optimized configurations of conformal predictive systems were statistically compared with a repeated measures ANOVA and the Tukey’s HSD *post-hoc* test at the 95% confidence level based on ⟨CRPS⟩ (Figure 3), after evaluating the statistical test assumptions (SI—METLIN-CCS 5 × 5 Cross-Validation—ANOVA + Tukey’s HSD). All pairwise differences were statistically significant, yielding the following ranking from lowest to highest ⟨CRPS⟩: GraphCCS, CCSP2.0, SigmaCCS, KerasECFP and LinMZ. The same ordering was observed for the arithmetic mean of *R* ^2^ scores and 90th percentiles of relative errors (SI—METLIN-CCS 5 × 5 Cross-Validation—Point-Estimation Performance). The consistent trend across the three evaluation metrics suggests that the architecture of the conformal predictive systems has negligible influence on the observed performance differences. Thus, here metrics based on point estimates already pinpoint which CCS models yield the tightest predictive distributions, although these performance measures do not strictly operate with uncertainties.

**Figure 3:**
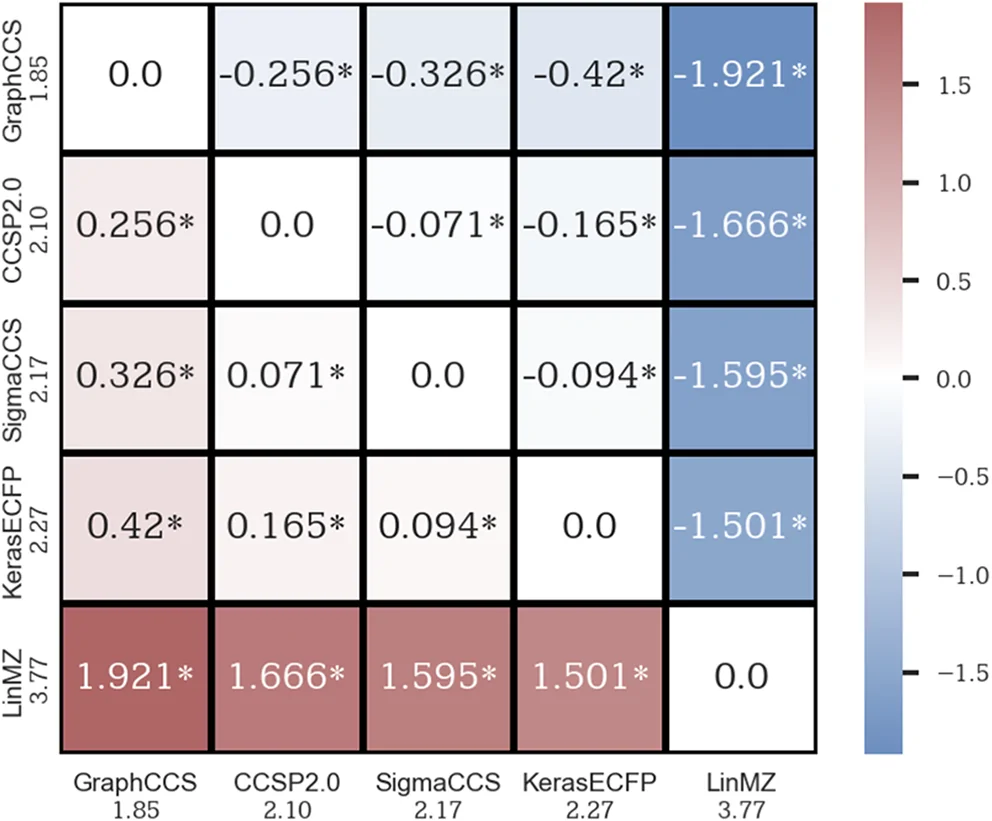
Results of the statistical comparisons with repeated measures ANOVA and Tukey’s HSD *post-hoc* test on ⟨CRPS⟩ (Å^2^), shown on the two axes, for the conformal predictive systems based on GraphCCS (κ: ion type, *n*: 2, *k*: 40), CCSP2.0 (κ: ion type, *n*: 2, *k*: 48), SigmaCCS (κ: ion type, *n*: 1, *k*: 40), KerasECFP (κ: ion type, *n*: 2, *k*: 48) and LinMZ (κ: ion type, *n*: 2, *k*: 84). Additionally, the number in each cell corresponds to the difference of ⟨CRPS⟩ between the conformal predictive systems on the *y*- and on the *x*-axes, color-coded from red to blue, with asterisks indicating statistically significant differences at 95% confidence.

A detailed analysis of the ⟨CRPS⟩ of models considered here suggests the presence of three performance groups. The first category consists solely of LinMZ; the second includes GraphCCS and the third comprises CCSP2.0, SigmaCCS as well as KerasECFP. Excluding the first group, which corresponds to the baseline regressor based on *m/z*, the remaining performance differences can be interpreted in the context of the features of each CCS model. Those in the third category rely on combinations of vectorial representations or graphs with neural networks or traditional ML algorithms, whereas GraphCCS is based on a deep graph neural network operating on ion graphs. The differential characteristics between these two groups suggest that the superior performance of GraphCCS may primarily arise from its more complex architectural depth, although the positive influence of the approximated ion structures cannot be excluded. As GraphCCS’s configuration ranked first, this model was selected for the final training, with *k* scaled up to 46 to account for the larger training set size.

### Recall at the Boundaries of the Applicability Domain

The performance of the OOD detection system was evaluated on the external AllCCS dataset using the final conformal predictive system configuration of GraphCCS. Firstly, a P^OOD^ threshold was found on the EnvCCS dataset by calculating empirical recall on ion species with a P^OOD^ lower than or equal to a cut-off value in the range of 40% to 100% in steps of 1% (Figure 4). For thresholds ≤90%, empirical recall lies close to target recall. In contrast, for thresholds >90%, it falls too low relative to target recall, which is indicated by empirical recall being lower than the lower bound given by the 5th binomial lower-tail percentile under target recall. This drop between the two ranges of P^OOD^ suggests that OOD detection plays an important role in selecting for which structures the system can be applied, so that it can guarantee a minimum empirical recall. On the other hand, the difference between empirical and target recall increases with decreasing α, falling continuously below the target recall in case of the 99% line. This trend indicates that levels corresponding to lower values of α, that is guaranteeing higher recall, are more difficult to calibrate, as they increasingly rely on the tails of the CCS predictive distributions with fewer calibration points. The importance of correcting $P^{CCS}$ for calibration-conditional validity is highlighted by the comparison with the analogous analysis without the proposed adjustment (Figure S28), where empirical recall consistently lies too low relative to target recall.

**Figure 4:**
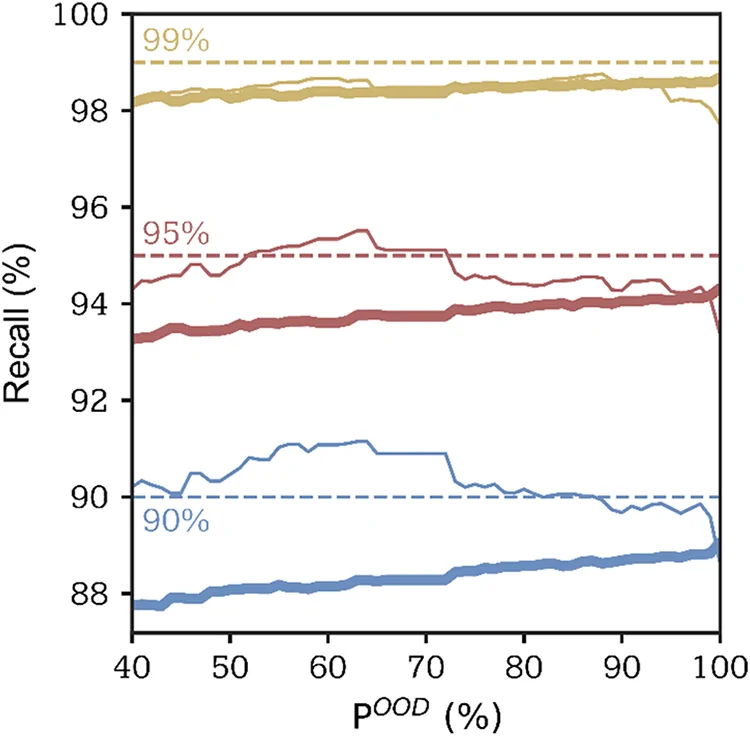
Thin solid lines show empirical recall for EnvCCS dataset at α levels 10% (blue), 5% (red) and 1% (yellow), calculated with ion species that have a P^OOD^ lower than or equal to a given threshold, which ranges from 40% to 100% -the latter corresponding to no filtering-, in steps of 1%. 40% was selected as the minimum threshold due to the limited number of ion species at lower values. Dashed horizontal colored lines indicate the corresponding target recall levels. Thick solid lines represent the 5th binomial lower-tail percentile under target recall.

It can then be empirically suggested that candidate structures with a P^OOD^ > 90% should be considered outside the model’s calibrated chemical space and, therefore, the conformal predictive system cannot be confidently used in those cases. To evaluate the generalizability of this empirical threshold found on EnvCCS, this cut-off value of 90% was applied to AllCCS and empirical recall was calculated across all α ∈ [0%, 100%]. Still, empirical recall was also found to lie close to the target recall for this dataset and above the lower binomial bound across all α levels (Figure 5A). This further suggests that 90% may be used as a default threshold for P^OOD^. The same analysis as the one presented in Figure 5A for EnvCCS can be found in Figure S29A.

**Figure 5:**
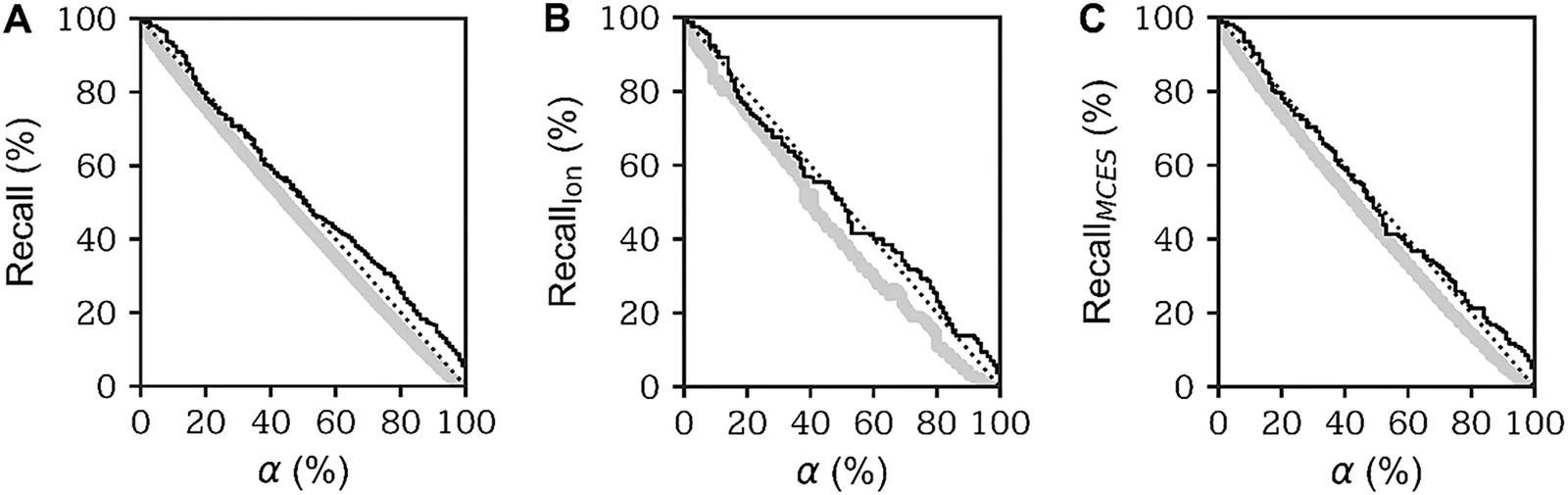
Recall (%) *vs* α (%) curves (A) across all AllCCS ion species with P^*OOD*^ ≤ 90% (*n*_test_^ID^ = 307), (B) for the ion type (*n*_test,[M+H]^+^_^ID^ = 157, *n*_test,[M+Na]^+^_^ID^ = 85, *n*_test,[M–H]^−^_^ID^ = 65) with the lowest empirical recall at each α value and (C) for the region of the ⟨MCES⟩_*k*=46_ dimension with the lowest empirical recall at each α value. The dotted black line indicates the target recall. The grey thicker line indicates the lower bound given by the 5th binomial lower-tail percentile under target recall. Note that only empirical recall in A and B is guaranteed by the Mondrian construction to lie close to the target recall. Also note that empirical recall does not reach 0% at α = 100% due to the calibration-conditional validity correction. To obtain the ⟨MCES⟩_*k*=46_ classes for (C), the equal-size discretization approach explained in Methods—Evaluation Measures—Recall was applied. This resulted in two classes with the following ⟨MCES⟩_*k*=46_ edges: 7.76–9.87–10.34.

We also evaluated the minimum empirical recall for the whole range of α ∈ [0%, 100%] across the different ion types, as this type of conditional empirical recall should also be assured by the Mondrian construction together with the OOD scorer. In this regard, the conformal predictive system can generally be considered well calibrated (Figure 5B) for estimated in-domain examples. The Mondrian taxonomy corresponding to the ion type [M + H]^+^ was almost always selected with the lowest empirical recall, as this taxonomy produces the tightest CCS predictive distributions of the three ion species and is consequently more sensitive to changes in chemical space. In fact, we found the calibration to be dependent on the random seed used to simulate the calibration sets for calibration-conditional validity correction: random seeds that led to a more conservative correction of the $P^{CCS}$ corresponding to the [M + H]^+^ taxonomy would increase calibration stability, and vice versa. We also evaluated if similar performance could be observed across the ⟨MCES⟩_*k*=46_ dimension (Figure 5C), which is not guaranteed by construction. Even so, empirical recall was found to consistently lie above the lower bound and close to target recall for estimated in-domain ion species. The construction of the conformal predictive system scoring metric, ⟨CRPS⟩, that assigns equal weight to all regions of the ⟨MCES⟩_*k*=46_ variable, may be the cause for this robustness. The region corresponding to lower ⟨MCES⟩_*k*=46_ values was always the one with lowest empirical recall, which may be due to the limited number of calibrants in that range. As a result thereof, it can be suggested that target recall guarantees can approximately be retained for NTS datasets containing a vast majority of candidate structures suggested for an ion type or that predominantly fall within a specific ⟨MCES⟩_*k*=46_ region. Similar results can be observed for the dataset EnvCCS (Figure S29B,C).

We report the JS distance between EnvCCS, containing only the ion species estimated to be in-domain, and the METLIN-CCS calibration set, which reaches 60%. In comparison, the JS distance between the METLIN-CCS training and calibration splits equals 7%. This difference aligns with the observation that EnvCCS predominantly comprises environmental pollutants, whereas METLIN-CCS corresponds to a broader chemical space, thereby resulting in a high JS distance. In contrast, the METLIN-CCS calibration split was specifically designed to overlap with the METLIN-CCS training chemical space, which leads to a low JS distance. The fact that the conformal predictive system was able to maintain its theoretical recall guarantees on an external dataset with considerable chemical space shift (increased JS distance) suggests that its architecture may be robust in real-world applications. We also calculated the JS distance between estimated in-domain AllCCS ion species and the METLIN-CCS calibration set as well as EnvCCS, which yielded 74% and 42%, respectively. The first distance result suggests that the conformal predictive system was able to maintain its theoretical recall guarantees on a dataset with a pronounced chemical shift compared to the METLIN-CCS calibrants, whereas the second value indicates a moderate chemical space divergence between EnvCCS and AllCCS. This difference would then show that the conformal predictive system maintained its theoretical recall guarantees in two external test datasets, each coming from partially non-overlapping chemical spaces, further consolidating its practical robustness.

All in all, MCES distances were shown to be a promising metric to aid in OOD detection. An applicability domain detection through graph-based molecular distances may potentially have benefits compared to approaches on fingerprints, descriptors or embeddings.^50,59,60,62,63^ Each vector representation has characteristic descriptive strengths and limitations,^64,65^ which also depend on the chosen OOD model or molecular distance metric.^66^ As a result, their ability to detect candidate structures outside the calibrated chemical space may depend on the types of compounds. From this perspective, MCES distances may offer a potentially broadly applicable alternative. Moreover, ⟨MCES⟩_*k*_ depends only on one learned parameter, *k*. Nevertheless, four aspects must be pointed out for caution. Firstly, ⟨MCES⟩_*k*_ is only a meaningful measure as long as the OOD calibrants belong to the same chemical space as the training compounds. Otherwise, a compound close to the training but far from the calibrated chemical spaces could show a low ⟨MCES⟩_*k*_, and thus obtain a low P^OOD^. Secondly, MCES distances may fail to capture pronounced changes in the structure-CCS landscape as a result of small molecular modifications. Such abrupt shifts are likely more predominant for molecular pairs sharing a substantial common subgraph, where the introduction of a bulky substituent in one molecule may induce a folding behavior different than what has been previously observed in the calibration set. In such cases, the MCES distance remains relatively unchanged, limiting its ability to capture these activity cliffs.^67^ Accordingly, ⟨MCES⟩_*k*_ is poorly suited for detecting such effects, which may explain the weak correlation between absolute errors and ⟨MCES⟩_*k*_ in Figure 2B. Thirdly, P^OOD^ is a measure of the OOD degree; however, it does not yield a formal OOD probability. Thus, there are no hard guarantees that the optimal cut-off value will be 90% across all possible samples or that the recall guarantees will be maintained, and it is always up to the end-user to decide which P^OOD^ threshold to apply. Fourthly, the proposed OOD scorer cannot detect instrumental bias, which will certainly invalidate the conformal predictive system.

On another note, OOD detection based on P^OOD^ > 90% results in a considerably constrained chemical space for the two test datasets. For EnvCCS, applying this cut-off value reduces the applicability of the conformal predictive system to 49% of the full dataset (*n*_test_^ID^ = 1414). For AllCCS, these metrics are further reduced to 31%, which corresponds to *n*_test_^ID^ = 307. We propose to overcome this constrained calibrated chemical space and expand the domain of applicability by tailoring the calibration step to a target chemical space with a specific corresponding dataset. We implemented EnvCCS itself as an alternative calibration set for environmentally relevant contaminants. Note that the measure ⟨MCES⟩_*k*_ for OOD detection needs to be additionally modified, as it assumes that the OOD calibrants come from the same chemical subspace as the training compounds. One possible strategy to overcome this constraint is to compute the calibration *s* ^OOD^ scores using leave-one-out estimates within the newly defined calibration set, while computing the test *s* ^OOD^ scores based on distances to these new calibrants. In this setting, we set the parameter *k* in ⟨MCES⟩_*k*_ at 14 by taking into account the ratio of ⟨MCES⟩_*k*_ training dataset size between METLIN-CCS and EnvCCS calibrations. This update implies that ⟨MCES⟩_*k*_ employed in the error function differs from the one used for OOD detection. With this new proposed calibrated chemical space based on EnvCCS, the applicability of the conformal predictive system for AllCCS increases to 80% of the full dataset (*n*_test_^ID^ = 796), when using the same P^OOD^ threshold of 90% found on EnvCCS with the original METLIN-CCS calibration. The increased number of estimated in-domain species is well aligned with the previously reported lower JS distance between AllCCS and EnvCCS compared to METLIN-CCS. In this case, empirical recall was also found to lie close to the target recall and above the lower binomial bound across all α levels (Figure S41).

In practice, the above-analysis implies for candidate structure prioritization in NTS that, if a percentage of true structures retained after filtering, *x*, is desired, one should set α at 100 – *x*, and the conformal predictive system will yield a recall around 100 – α; however, this is only valid in combination with the OOD detection system. Nevertheless, it is important to note that, though empirically supported, there is no dedicated theoretical analysis on our OOD scorer. Therefore, its ability to maintain empirical recall by the conformal predictive system close to target recall is not theoretically guaranteed. Finally, the calibrated chemical space can be tailored to a specific dataset, such as EnvCCS for environmental contaminants, in case OOD detection heavily constraints the domain of applicability.

### Efficiency in NTS Prioritization

One of the two aims of implementing IMS in NTS is removing the wrong candidate structures and prioritizing the correct ones. Thus, the applicability of the full CCS system in NTS was evaluated based on its capacity to (1) rank the true structure highest and (2) remove wrong candidate structures, *i.e.*, efficiency, while retaining true structures, *i.e.*, recall.

Candidate structure ranking based on CRPSs was applicable to the NTS features that showed all candidates to be estimated inside the applicability domain, that is, with a P^OOD^ ≤90%, which corresponded to 431 (34%) for EnvCCS_NTS and 103 (19%) for AllCCS_NTS. CRPS-based ranking resulted in no practically significant improvements compared to ranking based on CCS differences. For EnvCCS_NTS, 16.2% of true structures were correctly ranked first using CRPS, compared with 14.6% using CCS differences (10.7% random). The ground truth was ranked among the top 5 in 68.4% and 66.8% of the cases, respectively (51.2% random). For AllCCS_NTS, results were similar, with 15.5% *vs* 12.6% (12.8% random) for top 1 ranking performance, and 70.9% *vs* 71.8% (53.4% random) for top 5. This similarity in ranking arises because the range of ⟨MCES⟩_*k*=46_ for each feature is typically ≤1 a.u. (Figure S30). Thus, the CCS predictive distributions are nearly identical for candidate structures corresponding to one NTS feature in terms of distribution width, and their primary difference arises from their location determined by the CCS point estimate. The considerable similarity of the candidates for each feature, and thus their ranking independent of the CRPS or CCS differences, may also be a consequence of the high structure elucidation performance. Hence, higher differences could be observed in an experimental real-world scenario.

For the efficiency *vs* recall analysis, different α ∈ [0%, 100%] as in eq ([Disp-formula eq5]) were evaluated, where 0% indicates no candidate structure removal (Figure 6). The P^OOD^ > 90% condition for OOD detection from Figure 4, together with the correction for calibration-conditional validity, resulted in 43% and 52% of the wrong and true candidate structures, respectively, in EnvCCS_NTS that were retained independent of the *α* value. Consequently, the maximum achievable efficiency is 57%, limited mostly by the chemical space of METLIN-CCS used for model training and calibration and to a much lesser extent by the calibration-conditionally valid correction (Figure S6). Similar remarks can be made for AllCCS_NTS (Figure S31), where a higher proportion of candidate structures was estimated to be OOD, yielding a maximum achievable efficiency of 31%. Although the maximum achievable efficiency lies at 72% with EnvCCS calibration, instead of at 31% observed with METLIN-CCS, the empirical efficiency-recall line falls closer to random prioritization (Figure S42). This decay in prioritization performance is a consequence of CCS predictive distributions that are generally twice wider with EnvCCS than with METLIN-CCS calibrations, as a result of the higher absolute prediction errors made by GraphCCS on ion species reported in EnvCCS (Table S7).

**Figure 6:**
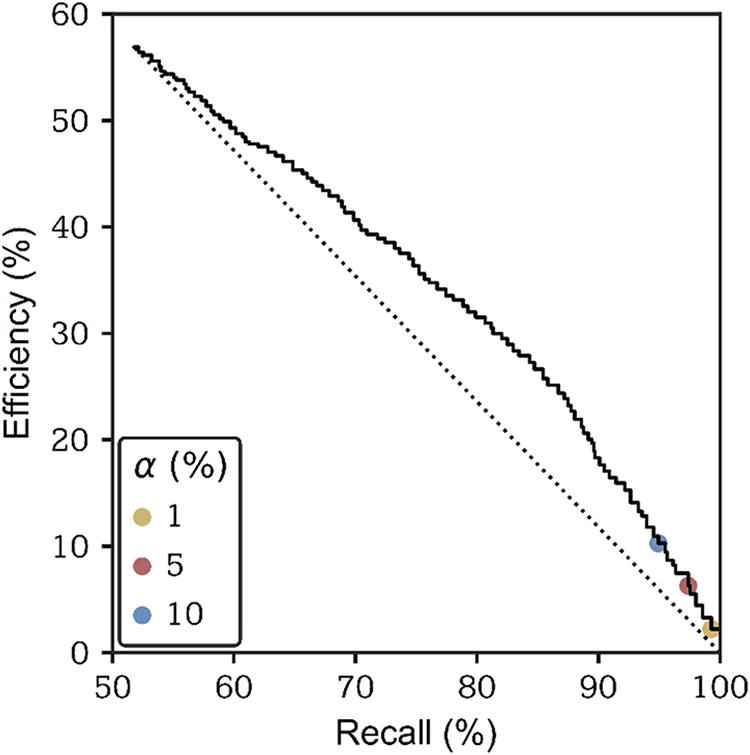
Trade-off between efficiency (percentage of wrong NTS candidate structures filtered out) and recall (percentage of correct NTS candidate structures retained), shown by the black solid line, when prioritizing structures corresponding to EnvCCS_NTS dataset as $P_{ccv}^{CCS}\geq \alpha$ at different α ∈ [0%, 100%], where 0% indicates no removal. If a candidate showed a P^OOD^ > 90% it was always retained, as it cannot be confidently evaluated with the conformal predictive system. As a result of OOD detection and the correction for calibration-conditional validity, 43% and 52% of the wrong and true candidate structures, respectively, were filtered-in across all *α*. The dotted black line indicates the theoretical performance of random prioritization. Yellow, red and blue scatter points indicate performances at α values 1%, 5% and 10%, respectively. Note that due to the candidates that were always retained, the random baseline lies above the diagonal and empirical recall appears higher than the target recall given by 100 – α.

A good prioritization performance would be indicated by high efficiency at high recall. In that regard, the conformal predictive system outperforms random prioritization for all recall levels, indicated in Figure 6 by the empirical efficiency-recall line lying above that of random prioritization. The difference in efficiencies at same recall is mostly ≤8% and decreases down to ≤4% for recall levels >90%, which corresponds to the range in which the method would presumably be primarily applied. A similar trend is observed for AllCCS_NTS dataset, with an efficiency delta in general ≤4% that is reduced to ≤2% for recall levels >90% (Figure S31). The small improvement over random prioritization results mainly from the limited CCS prediction accuracy, with an additional contribution from the error function. On the other hand, CCS alone has reduced discriminative power, as suggested by the <1% CCS accuracy necessary for NTS.^7^ Nevertheless, to put some perspective into the removal performance, it may be appropriate to consider here that the maximum achievable efficiency is 57% for EnvCCS_NTS and 31% for AllCCS_NTS. The maximum achievable efficiencies imply that efficiency-recall curves for EnvCCS_NTS and AllCCS_NTS datasets that were hypothetically fully in-domain and with no calibration-conditional correction would lie 1.75 and 3.22 times higher, respectively, at correspondingly rescaled recall. Consequently, the delta over random prioritization would also be proportionally higher. The efficiency-recall curves considering only NTS features for which the entire candidate list was estimated to be in-domain and for which the calibration-conditional validity correction yielded a $P_{ccv}^{CCS}$ < 100% can be found in the SI (Figures S32 and S33). On the other hand, prioritization of the candidate structures estimated to be in-domain based on 2% relative differences achieves a ∼32% efficiency at ∼78% recall for EnvCCS_NTS and ∼17% at 87% for AllCCS_NTS, which approximately coincides with the performance of the conformal predictive system at α = 46%. However, note that arbitrary thresholding, though widely discussed in the literature, will, in general, not guarantee a percentage of true structures prioritized after filtering, *i.e.*, recall.

Efficiency is expected, on average, to improve with tighter CCS predictive distributions. We analyze this conditional efficiency at α = 10%. Depending on [M + H]^+^, [M + Na]^+^ or [M – H]^−^, the conformal predictive system yielded 13.8%, 8.9% and 4.8% conditional efficiencies, respectively, for EnvCCS_NTS as well as 8.0%, 2.0% and 2.0% in the case of AllCCS_NTS. A similar trend can be observed for the mean MCES distance to the calibration chemical space of the candidates for each NTS feature (Figure S34). This occurs for candidate structures that lie closer to the calibrated chemical space, as measured by ⟨MCES⟩_*k*_, and which correspond to Mondrian taxonomies, *i.e.*, ion types, characterized by tighter error distributions (Figure 2A).

In terms of computational cost, the screening speed on a laptop equipped with 10 CPU cores (13th Gen Intel Core i7-1360P) and one GPU (NVIDIA RTX A500 Laptop GPU) amounts to 22 candidate structures per minute. In principle, the throughput can be increased approximately proportionally with the number of CPU cores employed, subject to parallelization efficiency.

## Conclusion

Candidate structure prioritization in NTS based on CCSs can be performed with suggested thresholds of relative differences between the measured CCS of an NTS feature and a prediction from a ML model.^8^ However, such a prioritization approach does not provide any theoretical statistical guarantees on the percentage of true structures retained after filtering among the candidate lists of the NTS features detected in a sample, *i.e.*, recall. In this study, we work within the statistical framework of conformal predictive systems^28,45^ to provide assurance on the recall. The developed system enables converting CCS point estimates from an existing CCS ML model, called GraphCCS,^18^ into predictive distributions. Furthermore, it accounts for the MCES distances to the training chemicals of GraphCCS (width of distribution) and for the ion type (shape of the distribution) of the candidate structure. Moreover, we propose a simple OOD detection approach that leverages the same molecular distances to identify compounds that lie outside the domain of applicability and to which the predictions should therefore not be applied. We utilize the extensive CCS dataset METLIN-CCS^9^ to fit both the conformal predictive system and the OOD detection system.

On two NTS datasets, we show that OOD detection is indeed key to ensuring that the predictions are applied only where reliability can be maintained. Otherwise, the theoretical statistical guarantees on the recall offered by the conformal predictive system vanish and a minimum percentage of retained true structures after prioritization cannot be assured. However, it should be noted that our proposed OOD detection method is heuristic in nature and serves as a practical safeguard supported empirically rather than by a dedicated theoretical analysis. In terms of prioritization performance, the percentage of wrong candidate structures removed, *i.e.*, removal efficiency, is low, while remaining 5% over that of random prioritization. We attribute the modest improvement over random prioritization to applicability domain limitations and small CCS differences between isomers associated with an NTS feature, which impose a remarkably stringent requirement for both CCS prediction accuracy and uncertainty quantification. These and previous findings by other researchers^24^ suggest that the practical utility of CCSs as an additional source of empirical analytical information for candidate structure prioritization in NTS may be limited.

In this regard, conformal predictive systems can also be extended to alternative analytical properties with potential for discrimination of candidate structures in NTS, such as retention times or adduct formation. The same theoretical recall guarantees explained in this work for CCS-based prioritization would then apply to each empirical property. This framework also opens opportunities for developing approaches to integrate probabilities associated with a given candidate structure across different molecular properties.

## Supplementary Material



## Data Availability

All data used in this study, except for the licensed commercial MS library NIST23,^35^ is publicly available and free of charge from previously published studies and their associated repositories. The NTS prioritization results, Jupyter notebooks with the additional error function experiments as well as for data pre-processing, and code for candidate structure prioritization in NTS with conformal predictive systems, is provided at: https://github.com/kruvelab/CCS-conformal-predictive-distributions (doi.org/10.5281/zenodo.21487070).

## Notes

Split and subsampling distributions; theoretical explanations of CRPSs and calibration-conditional validity; 5 × 5 cross-validation (point estimation, Mondrian taxonomies-point estimates and ion types-, error model −*n* & *k* and error correlation plots-, statistical tests—normality and variance assumption qualitative diagnostics, ANOVA results—and recall checks); point estimation performance of GraphCCS for EnvCCS and AllCCS; OOD detection performance without calibration-conditional validity and recall *vs* α for EnvCCS; histogram counts of the range of ⟨MCES⟩_*k*=46_ per NTS feature for EnvCCS_NTS as well as for AllCCS_NTS; efficiency *vs* recall curve for EnvCCS and AllCCS_NTS for all and fully ID NTS features; as well as conditional efficiency at α = 10% depending on ⟨⟨MCES⟩_*k*=46_⟩ for EnvCCS_NTS and AllCCS_NTS. Additionally, the same type of results for NTS prioritization are given for undistorted MS data and performance based on a new calibration chemical space with EnvCCS is also presented (PDF).

## Abbreviations

CCS: collision cross-section
IMS: ion-mobility spectrometry
MS: mass spectrometry/spectrometer
NTS: non-target screening
ML: machine learning
UQ: uncertainty quantification
OOD: out-of-domain
MCES: maximum common-edge subgraph
SI: Supporting Information
ECFP: extended connectivity fingerprint
*m*/*z*: mass-to-charge ratio
CRPS: continuous ranked probability score
ANOVA: analysis of variance
HSD: honestly significant difference
$\hat{CCS}$: CCS point estimate
$\hat{|e|}$: predicted absolute prediction error
*s* ^CCS^: CCS score
*c*: calibrant *c*
|*e*|: absolute prediction error
*s* ^′*CCS*^: transformed *s* ^CCS^
*κ*: Mondrian taxonomies
*a*⟨MCES⟩_*k*_^*n*^: arithmetic mean of MCES distances to the *k* nearest compounds in the METLIN-CCS training sets, for error function
$P^{CCS}$: probability for the measured CCS of the corresponding NTS feature to originate from the candidate’s predictive distribution
*p*: one-sided probability value
τ: random number sampled from the uniform distribution, τ ∼ *U*(0,1), needed to break ties in the calculation of *p*
*n*_cal_^CCS^: total number of finite CCS calibrants in the candidate’s distribution
α: prioritization threshold for $P^{CCS}$
CRPS: continuous ranked probability score
$P_{ccv}^{CCS}$: calibration-conditionally valid $P^{CCS}$
*h*: correction function for calibration-conditional validity
δ: parameter controlling the confidence of the calibration-conditional correction, as 1 – δ
ANOVA: analysis of variance
HSD: honestly significant difference
*s* ^OOD^: OOD score
P^OOD^: percentage of calibrants with an *s* ^OOD^ that is lower than or equal to the new ion species
*i*: training chemical for OOD detection
*n*_test_^ID^: total number of estimated in-domain test ion-species
*j*: chemical among *n*_test_^ID^
*N*(*j*): set of nearest neighbors for test chemical *j*
*t*_*i*_: normalized proportion of estimated in-domain test ion species for which *i* was selected as nearest neighbor
*T*: empirical discrete distribution for test chemical space
*n*_train_^OOD^: number of training chemicals for OOD detection
*N*(*c*): set of nearest neighbors for calibrant *c*
*t*_*i*_^′^: normalized proportion of calibrants for which *i* was selected as nearest neighbor
*C*: empirical discrete distribution for calibrated chemical space
