# Supplementary material for: Candidate Structure Prioritization in Non-Target Screening with Predicted Collision Cross-Sections and Uncertainty Quantification
Source: J Chem Inf Model. 2026 Sep 24;66(19):12534–48. doi: 10.1021/acs.jcim.6c02492 (PMC13663483; doi:10.1021/acs.jcim.6c02492)
Supplement: Supplementary file 1 [file ci6c02492_si_001.pdf]

Candidate Structure Prioritization in Non-Target Screening with Predicted Collision Cross-Sections and Uncertainty Quantification

Lucas Ferrando Plo[a] *and Anneli Kruve**[a,b]

[a]Department of Chemistry, Stockholm University, 11418 Stockholm, Sweden.

*E-mail: anneli.kruve@su.se

[b]Department of Environmental Science, Stockholm University, 11418 Stockholm, Sweden

Split Distributions ---------- 3
Subsampling Distributions ---------- 6
CRPS ---------- 8
Calibration-Conditional Validity ---------- 9
METLIN-CCS 5x5 Cross-Validation ---------- 12
Point-Estimation Performance ---------- 12
Mondrian Taxonomies ---------- 18
Point Estimates ---------- 18
Ion Types ---------- 23
Error Function ---------- 28
$\boldsymbol{n}$ & $\boldsymbol{k}$ ---------- 28
Correlation Plots ---------- 32
ANOVA + Tukey's HSD ---------- 36
Recall ---------- 38
Point Estimation Performance for EnvCCS and AllCCS ---------- 40
Recall at the Boundaries of the Applicability Domain ---------- 41
Efficiency in NTS Prioritization ---------- 43
NTS Prioritization with Raw MS Data ---------- 48
EnvCCS Calibration ---------- 54
References ---------- 60

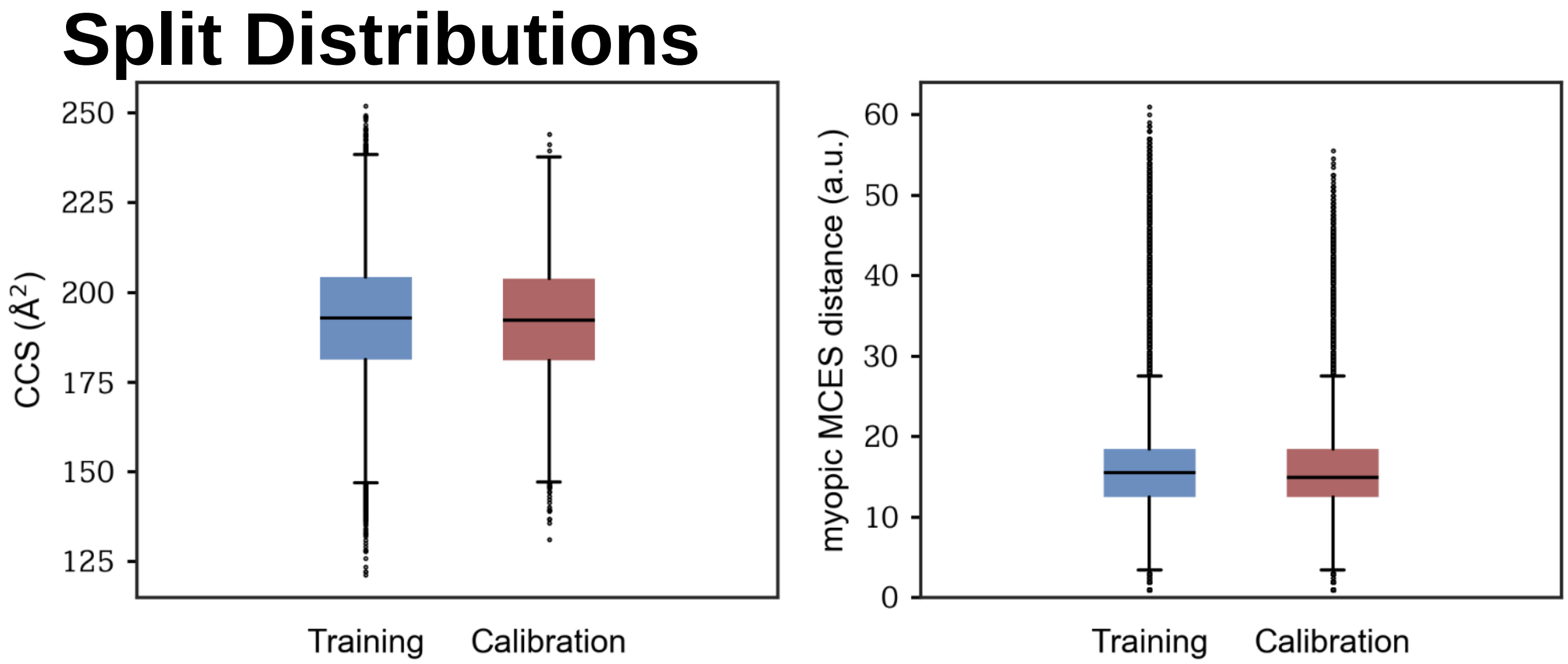

Figure S1. Distribution of CCS (Å^2, left) and myopic MCES distances[1] with myopic threshold 10 (a.u., right) for the final METLIN-CCS training (blue) and calibration (red) sets. Note that the molecular distance for each pair of compounds can be included several times for the different ion types.

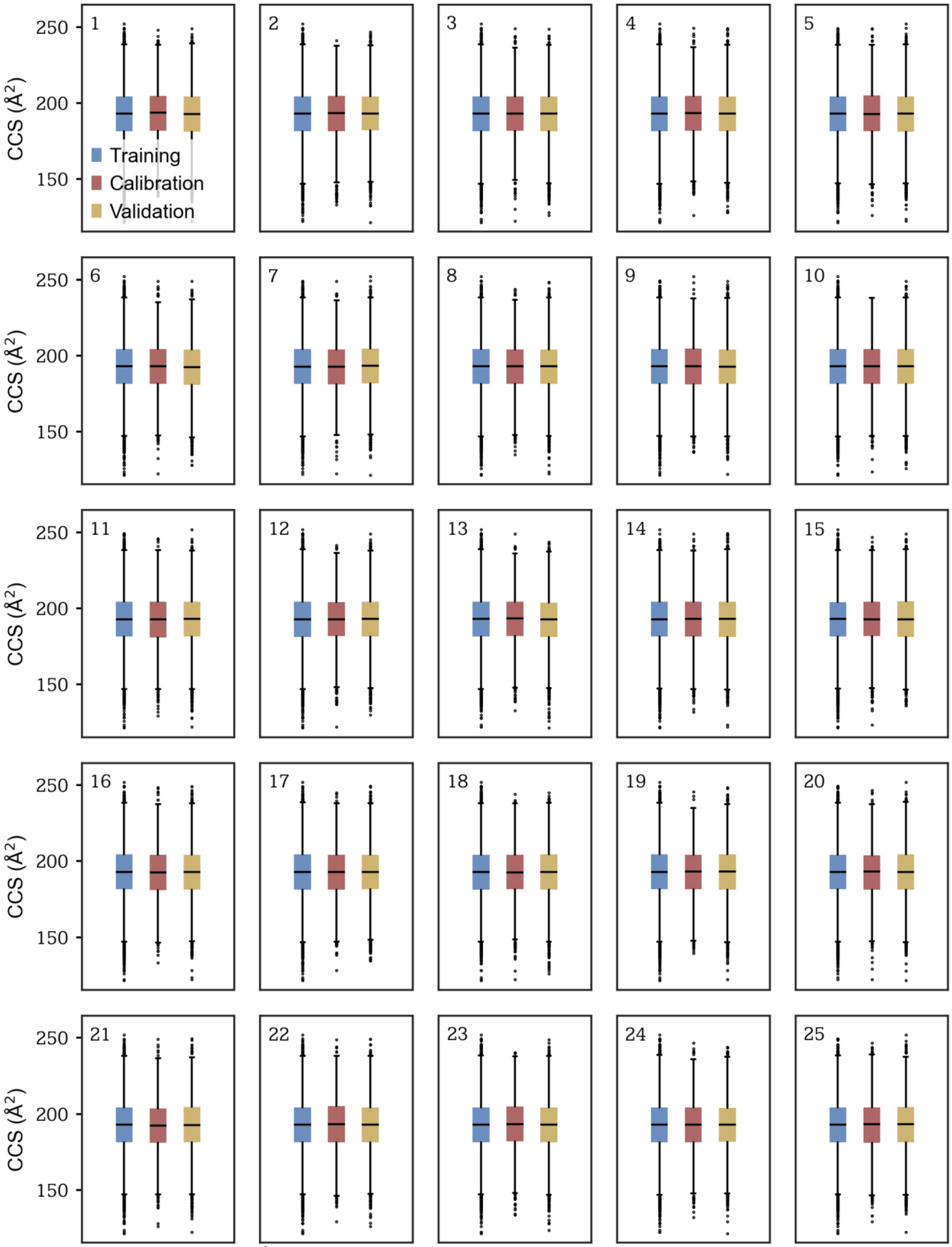

Figure S2. Distribution of CCS (Å^2) for the training (blue), calibration (red) and validation (yellow) sets across the 25 folds of the 5x5 cross-validation on METLIN-CCS.

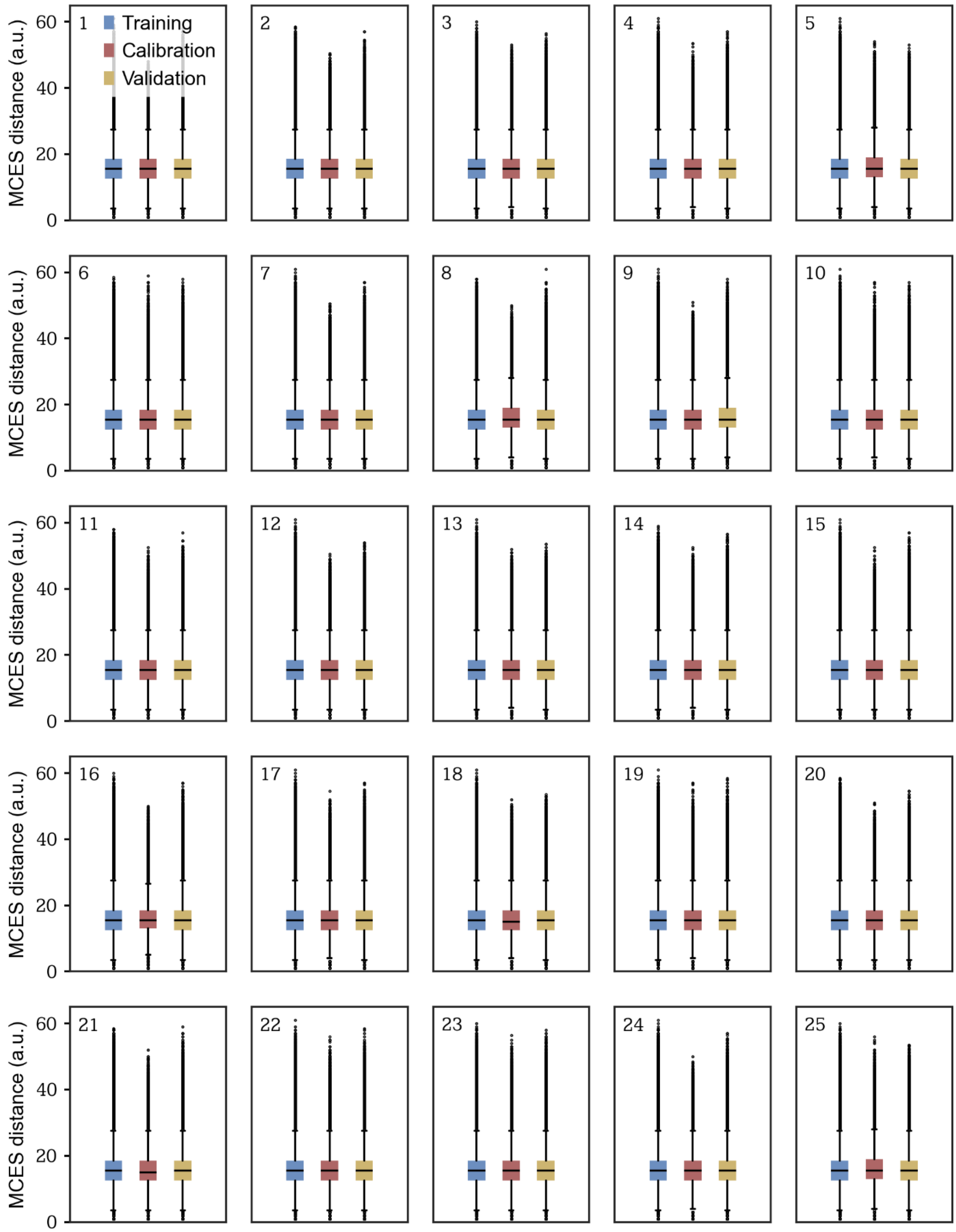

Figure S3. Distribution of the myopic MCES distances[1] with myopic threshold 10 for the training (blue), calibration (red) and validation (yellow) sets across the 25 folds of the 5x5 cross-validation on METLIN-CCS. Note that the molecular distance for each pair of compounds can be included several times for the different ion types.

Subsampling Distributions

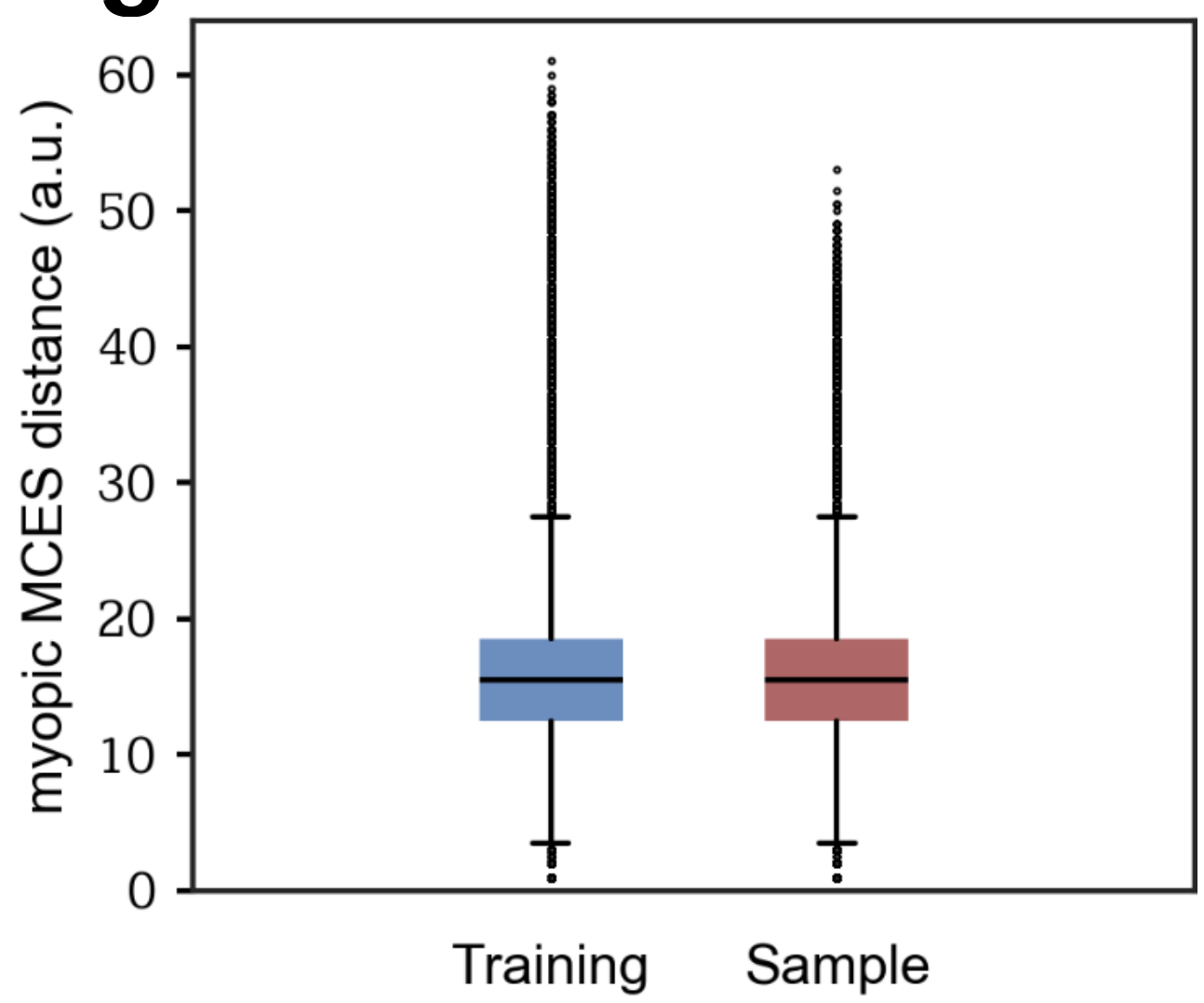

Figure S4. Distribution of the myopic MCES distances[1] with myopic threshold 10 for the final METLIN-CCS training set (blue) and a sample from it (red). Note that the molecular distance for each pair of compounds was included only once, regardless of the presence of different ion types.

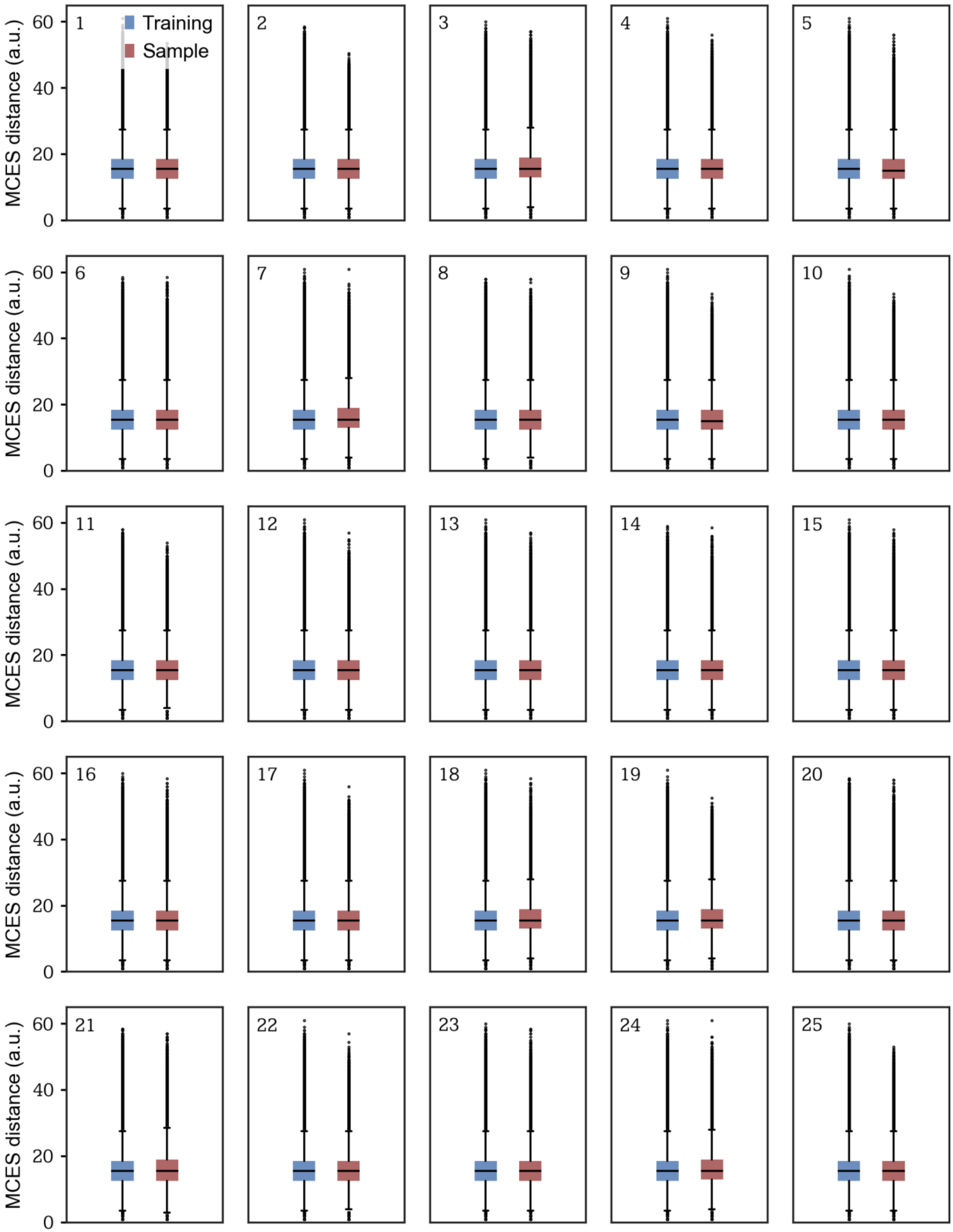

Figure S5. Distribution of the myopic MCES distances[1] with myopic threshold 10 for the training set (blue) and a sample from it (red) for the 25 folds in the 5x5 cross-validation on METLIN-CCS. Note that the molecular distance for each pair of compounds was included only once, regardless of the presence of different ion types.

CRPS

The CRPS is implemented to quantify the agreement between a CCS predictive distribution, PD, and a given CCS value, CCS. It is formally defined as follows:

$$\int_{-\infty}^{\infty} (PD(x) - \mathbb{1}_{x \geq CCS})^2 dx, \tag{S1}$$

where $\mathbb{1}$ denotes the indicator function.

Calibration-Conditional Validity

A concise summary of the work of Bates *et al.*[2] will be presented here for completeness. $\mathbb{P}^{CCS}$ generated by the standard framework of conformal predictive systems through Equation (3) in the main text are valid over both the calibration and external sets. As the intended outcome of this work is to build a final conformal predictive system with fixed calibrants, $\mathbb{P}^{CCS}$ validity needs to be conditioned on the CCS calibration set. To do so, one can find a piece-wise constant correction function, h, for $\mathbb{P}^{CCS}$, so that

$$\mathbb{P}_{ccv}^{CCS} = h(\mathbb{P}^{CCS}) = 100 b_{(\lceil (n_{cal}^{CCS}+1)\mathbb{P}^{CCS}/100 \rceil)}, \quad \text{(S2)}$$

where $\mathbb{P}_{ccv}^{CCS}$ is the calibration-conditionally valid $\mathbb{P}^{CCS}$ and $b_{(c)} \leq b_{(c+1)}$ for $c \in \{1, \dots, n_{cal}^{CCS}\}$ are the correction steps with $b_{(0)} = 0$ and $b_{(n_{cal}+1)} = 1$ (Figure S6). A Monte-Carlo approach to check the validity of the proposed correction function is that h has to satisfy $\mathbb{P}\left[u_{(\mathrm{c})} \leq b_{(\mathrm{c})}, \forall c\right] \geq 1 - \delta$, where each u_c is independently drawn from $U(0,1)$ and $1 - \delta$ is the confidence of the correction. In this work, $\delta = 0.05$ and the number of Monte-Carlo samples, *i.e.*, simulated calibration sets, is fixed at 10,000.

One option for h is to apply the generalized Simes inequality:

$$b_{(n_{cal}^{CCS}+1-c)}^{Simes} = 1 - \delta^{\frac{1}{k}} \left(\frac{c(c-1)\cdots(c-k+1)}{n_{cal}^{CCS}(n_{cal}^{CCS}-1)\cdots(n_{cal}^{CCS}-k+1)} \right)^{\frac{1}{k}}. \quad \text{(S3)}$$

A correction function produced by Equation (S3) already satisfies the validity check. However, it does not yield a reasonably tight solution across [0,1], which, in turn, can be modulated by the parameter k. Another possibility is to use an asymptotic adjustment as $n_{cal}^{CCS} \to \infty$:

$$b_{(c)}^{as} = \min\left(\frac{c}{n_{cal}^{CCS}} + d_{n_{cal}}(\delta) \frac{\sqrt{c(n_{cal}^{CCS}-c)}}{n_{cal}^{CCS}\sqrt{n_{cal}^{CCS}}}, 1 \right), \quad \text{(S4)}$$

where $d_{n_{cal}^{CCS}}(\delta) = \frac{-\ln[-\ln(1-\delta)] + 2\ln\ln n_{cal}^{CCS} + 1/2\ln\ln\ln n_{cal}^{CCS} - 1/2\ln\pi}{\sqrt{2\ln\ln n_{cal}^{CCS}}}$. Equation (S4) has the advantage that it provides reasonably tight bounds across the whole range. However, it only satisfies the validity check at the limit of infinite number of calibrants, so it is not applicable for finite calibration sets.

The solution proposed by Bates *et al.*[2] is to apply a hybrid adjustment composed of Equation (S3) and Equation (S4):

$$h^{hyb} = \min\left(h^{Simes}, h^{as}\right). \tag{S5}$$

With $k = n_{cal}/2$ in Equation (S3), h^{Simes} is modulated to be the correction selected for lower values of c, whereas h^{as} tends to yield a tighter envelope at higher values of c. Additionally, Equation (S4) needs to be modified by finding an updated δ, $\hat{\delta}$, to adjust for the calibration set finiteness. This is done with a bisection search for the root of $\delta - \mathbb{P}\left[u_{(c)} > b_{(c)}^{hyb}(\hat{\delta}), \forall c\right]$ for $\hat{\delta}$ in (0,1). Equation (S4) is further modified by taking the tangent line at $c = \frac{n_{cal}^{CCS}}{2}$ for $c \geq \frac{n_{cal}^{CCS}}{2}$. This linear extension is implemented to loosen the asymptotic envelope at high c and, thus, tighten the hybrid envelope for lower values of $\mathbb{P}^{CCS}$, where candidate structure prioritization is critical.

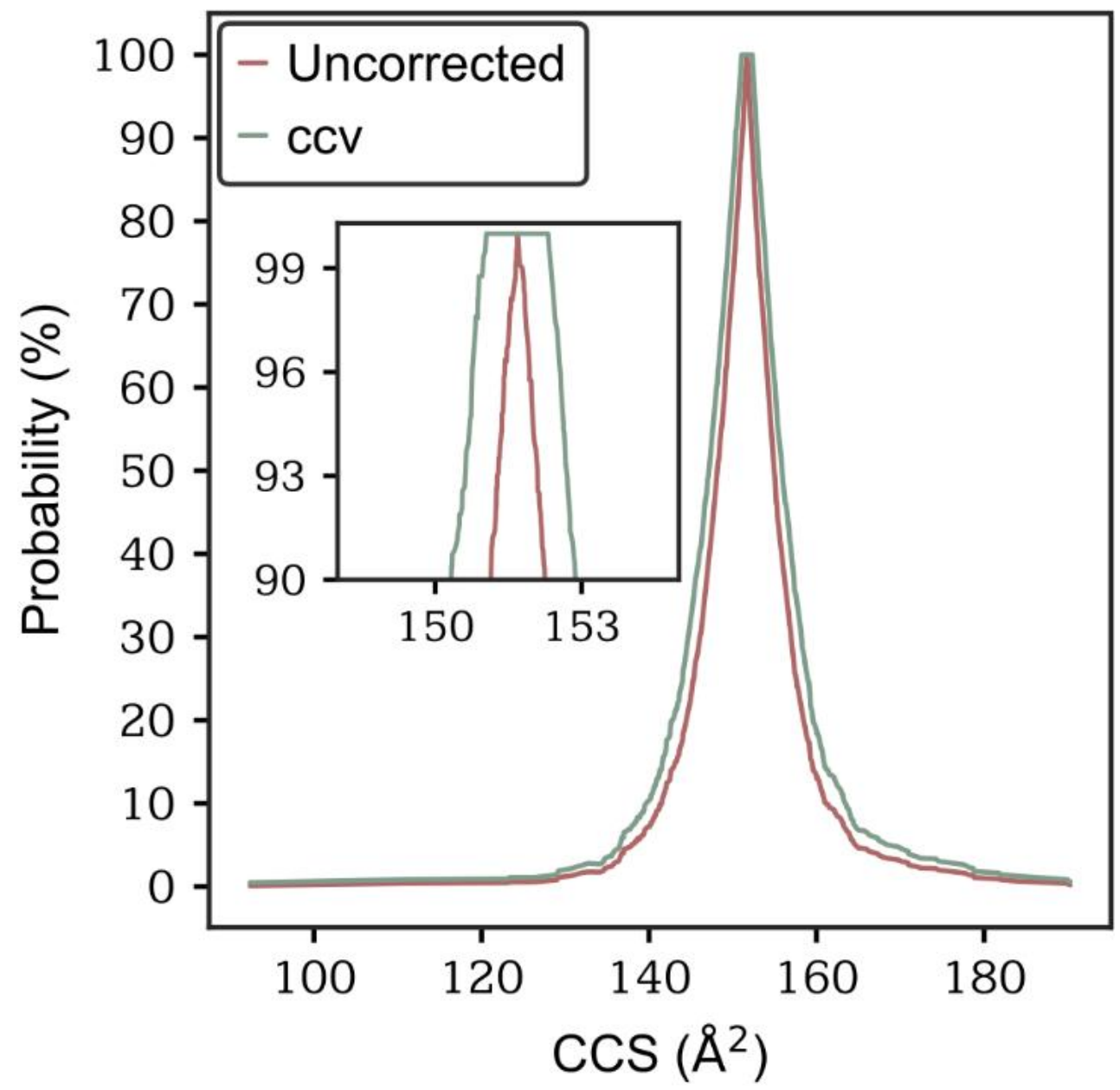

Figure S6. Probability (%) for the 1557 calibration CCS values of a given predictive distribution with (green, $\mathbb{P}_{ccv}^{CCS}$) and without (red, $\mathbb{P}^{CCS}$) correction for calibration-conditional validity. The zoomed-in plot shows the effect that the hybrid envelope has on CCSs with a high $\mathbb{P}^{CCS}$, leading to a plateau of $\mathbb{P}_{ccv}^{CCS}$ at 100% instead of a sharp peak as with $\mathbb{P}^{CCS}$. Consequently, candidate structures with a $\mathbb{P}_{ccv}^{CCS}$ equal to 100% are, in practice, considered as a perfect match with the NTS measured CCS and will be retained across any $\alpha \in [0\%, 100\%]$ as in Equation (5) in the main text.

METLIN-CCS 5x5 Cross-Validation

Point-Estimation Performance

Table S1. Point-estimation performance metrics for GraphCCS across the METLIN-CCS 25 5x5 cross-validation folds and their arithmetic mean.

Fold	R^2_{train}	$RMSE_{train}$	MAE_{train}	$MdAE_{train}$	$MAPE_{train}$	$^{90}PAE_{train}$	R^2_{cal}	$RMSE_{cal}$	MAE_{cal}	$MdAE_{cal}$	$MAPE_{cal}$	$^{90}PAE_{cal}$	R^2_{val}	$RMSE_{val}$	MAE_{val}	$MdAE_{val}$	$MAPE_{val}$	$^{90}PAE_{val}$
1	0.918	4.72	3.36	2.46	1.74	3.71	0.903	5.11	3.60	2.63	1.86	3.95	0.913	4.90	3.50	2.59	1.81	3.86
2	0.923	4.57	3.26	2.38	1.69	3.64	0.911	4.91	3.50	2.54	1.81	3.94	0.905	5.04	3.60	2.61	1.85	4.02
3	0.920	4.66	3.31	2.41	1.71	3.69	0.909	4.94	3.53	2.55	1.82	3.88	0.909	4.93	3.50	2.54	1.81	3.85
4	0.928	4.42	3.17	2.33	1.64	3.51	0.907	5.00	3.54	2.55	1.82	3.93	0.906	5.02	3.52	2.48	1.82	3.96
5	0.925	4.50	3.23	2.38	1.66	3.55	0.912	4.95	3.49	2.51	1.79	3.87	0.910	4.96	3.50	2.54	1.81	3.84
6	0.926	4.49	3.19	2.31	1.65	3.55	0.910	4.90	3.46	2.48	1.79	3.91	0.912	4.90	3.48	2.53	1.81	3.90
7	0.924	4.53	3.22	2.34	1.67	3.58	0.912	4.85	3.45	2.52	1.78	3.75	0.909	4.94	3.50	2.53	1.80	3.89
8	0.919	4.68	3.36	2.46	1.73	3.71	0.905	5.04	3.64	2.69	1.87	4.07	0.906	5.01	3.52	2.58	1.82	3.85
9	0.939	4.06	2.93	2.17	1.51	3.21	0.907	5.06	3.56	2.53	1.83	3.88	0.906	5.05	3.53	2.52	1.82	3.96
10	0.922	4.59	3.28	2.40	1.69	3.63	0.909	4.97	3.48	2.48	1.80	3.82	0.912	4.90	3.49	2.57	1.80	3.84
11	0.917	4.73	3.40	2.50	1.75	3.75	0.908	5.03	3.55	2.59	1.84	3.88	0.908	4.99	3.54	2.55	1.83	3.97
12	0.937	4.12	2.97	2.21	1.54	3.30	0.912	4.89	3.47	2.49	1.80	3.91	0.907	5.08	3.60	2.60	1.86	4.07
13	0.940	4.04	2.93	2.17	1.51	3.25	0.902	5.14	3.61	2.61	1.86	4.01	0.902	5.10	3.60	2.61	1.87	4.06
14	0.923	4.56	3.26	2.38	1.69	3.66	0.909	4.95	3.54	2.65	1.83	3.95	0.907	4.99	3.48	2.51	1.81	3.78
15	0.921	4.63	3.33	2.44	1.71	3.67	0.902	5.25	3.58	2.55	1.85	3.90	0.912	4.88	3.51	2.58	1.80	3.84
16	0.952	3.59	2.63	1.98	1.36	2.87	0.903	5.14	3.61	2.58	1.86	4.09	0.896	5.32	3.65	2.57	1.89	4.04
17	0.920	4.66	3.34	2.48	1.72	3.64	0.907	5.00	3.56	2.62	1.83	3.89	0.910	4.90	3.50	2.57	1.80	3.81
18	0.932	4.28	3.08	2.26	1.59	3.41	0.911	4.88	3.49	2.53	1.80	3.95	0.908	5.00	3.56	2.59	1.84	3.98
19	0.922	4.60	3.27	2.41	1.70	3.64	0.906	4.96	3.54	2.59	1.83	3.99	0.909	4.99	3.53	2.54	1.83	3.94
20	0.917	4.75	3.41	2.51	1.76	3.77	0.901	5.16	3.66	2.66	1.90	4.05	0.910	4.92	3.53	2.62	1.82	3.92
21	0.919	4.68	3.37	2.49	1.73	3.68	0.901	5.11	3.57	2.60	1.85	3.87	0.912	4.88	3.50	2.61	1.80	3.79
22	0.918	4.70	3.35	2.45	1.73	3.73	0.907	5.05	3.57	2.63	1.85	3.95	0.910	4.93	3.51	2.62	1.81	3.84
23	0.921	4.61	3.29	2.41	1.70	3.66	0.911	4.90	3.49	2.57	1.80	3.80	0.905	5.09	3.61	2.61	1.87	3.99
24	0.920	4.66	3.34	2.46	1.72	3.68	0.905	5.06	3.57	2.62	1.84	3.86	0.910	4.91	3.49	2.53	1.80	3.87
25	0.912	4.88	3.52	2.61	1.82	3.90	0.914	4.88	3.54	2.59	1.83	3.97	0.902	5.15	3.67	2.69	1.90	4.11
**AVG**	0.925	4.51	3.23	2.38	1.67	3.57	0.907	5.01	3.54	2.57	1.83	3.92	0.908	4.99	3.54	2.57	1.83	3.92

R^2: R^2 score; $RMSE$: root-mean-squared error; MAE: mean absolute error; $MdAE$: median absolute error; $MAPE$: mean absolute percentage error; ^{90}PAE: 90th percentile of percentage absolute errors. $train$: training set; cal: calibration set; val: validation set.

Table S2. Point-estimation performance metrics for CCSP2.0 across the METLIN-CCS 25 5x5 cross-validation folds and their arithmetic mean.

Fold	R^2_{train}	$RMSE_{train}$	MAE_{train}	$MdAE_{train}$	$MAPE_{train}$	$^{90}PAE_{train}$	R^2_{cal}	$RMSE_{cal}$	MAE_{cal}	$MdAE_{cal}$	$MAPE_{cal}$	$^{90}PAE_{cal}$	R^2_{val}	$RMSE_{val}$	MAE_{val}	$MdAE_{val}$	$MAPE_{val}$	$^{90}PAE_{val}$
1	0.897	5.27	3.79	2.77	1.96	4.23	0.885	5.56	3.98	2.95	2.05	4.26	0.897	5.31	3.83	2.80	1.97	4.29
2	0.898	5.27	3.78	2.76	1.95	4.23	0.897	5.27	3.82	2.83	1.98	4.25	0.890	5.44	3.95	2.90	2.03	4.41
3	0.898	5.27	3.78	2.75	1.95	4.25	0.889	5.44	3.94	2.93	2.03	4.30	0.893	5.36	3.87	2.88	2.00	4.33
4	0.899	5.24	3.78	2.77	1.95	4.24	0.891	5.41	3.89	2.85	2.00	4.42	0.888	5.48	3.94	2.89	2.03	4.37
5	0.897	5.27	3.79	2.76	1.95	4.24	0.898	5.33	3.85	2.87	1.98	4.22	0.893	5.41	3.90	2.87	2.02	4.34
6	0.896	5.30	3.80	2.79	1.96	4.24	0.895	5.27	3.82	2.75	1.97	4.27	0.898	5.30	3.86	2.83	2.00	4.32
7	0.898	5.27	3.79	2.78	1.96	4.22	0.895	5.33	3.89	2.90	2.01	4.40	0.891	5.39	3.88	2.85	2.00	4.34
8	0.898	5.26	3.78	2.75	1.95	4.25	0.892	5.35	3.91	2.88	2.01	4.40	0.891	5.42	3.90	2.89	2.02	4.32
9	0.898	5.26	3.77	2.75	1.95	4.20	0.896	5.35	3.91	2.90	2.01	4.30	0.889	5.48	3.93	2.87	2.03	4.41
10	0.897	5.27	3.79	2.76	1.95	4.23	0.890	5.45	3.90	2.81	2.01	4.33	0.895	5.36	3.88	2.88	2.00	4.27
11	0.897	5.26	3.79	2.76	1.96	4.22	0.890	5.48	3.91	2.89	2.03	4.30	0.893	5.38	3.88	2.84	2.00	4.35
12	0.897	5.26	3.78	2.76	1.95	4.22	0.898	5.26	3.82	2.79	1.97	4.22	0.893	5.46	3.95	2.89	2.04	4.36
13	0.898	5.27	3.79	2.76	1.95	4.25	0.890	5.46	3.90	2.87	2.01	4.29	0.891	5.38	3.89	2.88	2.01	4.35
14	0.898	5.26	3.79	2.76	1.95	4.25	0.894	5.36	3.90	2.85	2.01	4.36	0.890	5.44	3.88	2.83	2.00	4.32
15	0.897	5.27	3.79	2.77	1.96	4.24	0.889	5.56	3.89	2.83	2.01	4.39	0.896	5.29	3.88	2.89	2.00	4.31
16	0.900	5.20	3.76	2.76	1.94	4.21	0.891	5.45	3.94	2.94	2.03	4.40	0.884	5.61	3.96	2.85	2.05	4.38
17	0.898	5.28	3.79	2.77	1.96	4.23	0.887	5.49	3.93	2.87	2.03	4.39	0.893	5.34	3.86	2.81	1.99	4.37
18	0.896	5.30	3.80	2.78	1.96	4.25	0.898	5.23	3.87	2.89	2.00	4.29	0.896	5.31	3.86	2.85	1.99	4.27
19	0.897	5.27	3.78	2.75	1.95	4.23	0.889	5.38	3.89	2.88	2.01	4.36	0.895	5.36	3.90	2.89	2.02	4.32
20	0.897	5.29	3.79	2.77	1.96	4.23	0.888	5.48	3.92	2.82	2.02	4.29	0.895	5.31	3.86	2.86	1.99	4.31
21	0.897	5.28	3.80	2.78	1.96	4.25	0.884	5.53	3.92	2.87	2.04	4.43	0.897	5.29	3.83	2.81	1.98	4.21
22	0.897	5.28	3.79	2.76	1.96	4.24	0.891	5.47	3.98	2.99	2.06	4.43	0.895	5.32	3.83	2.80	1.98	4.29
23	0.899	5.24	3.77	2.76	1.94	4.21	0.895	5.31	3.84	2.87	1.98	4.17	0.889	5.52	3.99	2.92	2.06	4.46
24	0.898	5.26	3.78	2.75	1.95	4.22	0.889	5.45	3.93	2.89	2.03	4.35	0.892	5.38	3.91	2.91	2.02	4.31
25	0.897	5.27	3.79	2.77	1.95	4.23	0.901	5.23	3.83	2.83	1.98	4.28	0.891	5.43	3.89	2.84	2.01	4.38
**AVG**	0.898	5.27	3.79	2.76	1.95	4.23	0.892	5.40	3.90	2.87	2.01	4.32	0.893	5.39	3.89	2.86	2.01	4.34

R^2: R^2 score; $RMSE$: root-mean-squared error; MAE: mean absolute error; $MdAE$: median absolute error; $MAPE$: mean absolute percentage error; ^{90}PAE: 90th percentile of percentage absolute errors. $train$: training set; cal: calibration set; val: validation set.

Table S3. Point-estimation performance metrics for SigmaCCS across the METLIN-CCS 25 5x5 cross-validation folds and their arithmetic mean.

Fold	R^2_{train}	$RMSE_{train}$	MAE_{train}	$MdAE_{train}$	$MAPE_{train}$	$^{90}PAE_{train}$	R^2_{cal}	$RMSE_{cal}$	MAE_{cal}	$MdAE_{cal}$	$MAPE_{cal}$	$^{90}PAE_{cal}$	R^2_{val}	$RMSE_{val}$	MAE_{val}	$MdAE_{val}$	$MAPE_{val}$	$^{90}PAE_{val}$
1	0.905	5.06	3.80	3.02	1.99	4.16	0.882	5.62	4.20	3.32	2.19	4.53	0.889	5.52	4.15	3.28	2.17	4.51
2	0.891	5.44	4.06	3.16	2.08	4.27	0.885	5.58	4.19	3.23	2.15	4.41	0.876	5.76	4.30	3.34	2.20	4.54
3	0.890	5.47	4.12	3.26	2.11	4.31	0.870	5.90	4.38	3.40	2.24	4.59	0.874	5.81	4.36	3.40	2.23	4.59
4	0.902	5.16	3.88	3.08	2.03	4.20	0.879	5.70	4.26	3.28	2.21	4.58	0.876	5.76	4.28	3.32	2.23	4.65
5	0.903	5.12	3.86	3.06	2.02	4.24	0.883	5.69	4.22	3.20	2.20	4.75	0.876	5.82	4.28	3.26	2.24	4.72
6	0.903	5.11	3.79	2.95	1.95	4.05	0.889	5.42	4.02	3.12	2.07	4.32	0.894	5.40	4.01	3.09	2.07	4.34
7	0.912	4.90	3.64	2.83	1.89	3.98	0.885	5.57	4.13	3.15	2.14	4.56	0.886	5.53	4.07	3.11	2.10	4.47
8	0.904	5.11	3.82	2.96	1.96	4.12	0.874	5.79	4.29	3.33	2.20	4.62	0.876	5.76	4.25	3.24	2.18	4.52
9	0.908	4.98	3.71	2.85	1.93	4.07	0.887	5.60	4.11	3.09	2.14	4.62	0.883	5.62	4.12	3.12	2.14	4.53
10	0.911	4.91	3.64	2.81	1.88	3.94	0.887	5.53	4.07	3.14	2.10	4.34	0.891	5.44	4.03	3.11	2.08	4.34
11	0.909	4.96	3.70	2.91	1.93	4.01	0.887	5.57	4.07	3.16	2.12	4.48	0.887	5.53	4.05	3.06	2.10	4.48
12	0.901	5.15	3.81	2.92	1.97	4.17	0.896	5.31	3.92	3.00	2.03	4.32	0.890	5.52	4.05	3.05	2.10	4.46
13	0.888	5.52	4.24	3.48	2.23	4.54	0.863	6.10	4.56	3.64	2.39	4.91	0.862	6.05	4.60	3.70	2.42	4.99
14	0.899	5.23	3.96	3.14	2.07	4.32	0.876	5.79	4.39	3.51	2.29	4.68	0.874	5.81	4.34	3.41	2.27	4.74
15	0.914	4.83	3.58	2.77	1.86	3.90	0.888	5.59	3.99	2.99	2.08	4.37	0.895	5.34	3.94	2.99	2.03	4.30
16	0.916	4.77	3.54	2.72	1.83	3.87	0.889	5.50	4.04	3.08	2.08	4.34	0.882	5.66	4.04	2.98	2.09	4.47
17	0.915	4.81	3.56	2.76	1.86	3.92	0.888	5.47	4.03	3.07	2.09	4.43	0.888	5.48	4.04	3.06	2.09	4.43
18	0.906	5.03	3.80	3.01	1.95	4.00	0.883	5.61	4.27	3.41	2.20	4.55	0.883	5.65	4.25	3.29	2.19	4.54
19	0.893	5.38	4.07	3.24	2.08	4.25	0.866	5.90	4.44	3.40	2.27	4.74	0.874	5.86	4.42	3.46	2.26	4.60
20	0.889	5.49	4.12	3.26	2.16	4.49	0.872	5.85	4.35	3.39	2.27	4.80	0.881	5.65	4.26	3.37	2.22	4.63
21	0.902	5.15	3.89	3.06	2.03	4.27	0.869	5.90	4.31	3.31	2.26	4.74	0.877	5.77	4.30	3.32	2.24	4.71
22	0.910	4.92	3.63	2.79	1.87	3.91	0.886	5.61	4.11	3.07	2.11	4.48	0.891	5.44	3.99	3.02	2.05	4.36
23	0.878	5.73	4.46	3.69	2.34	4.80	0.849	6.38	4.83	3.82	2.53	5.24	0.846	6.48	4.93	3.92	2.59	5.39
24	0.913	4.87	3.61	2.78	1.85	3.87	0.883	5.60	4.06	2.98	2.09	4.49	0.887	5.50	4.05	3.08	2.08	4.34
25	0.876	5.78	4.57	3.88	2.41	4.84	0.860	6.23	4.88	4.09	2.57	5.30	0.846	6.47	5.01	4.16	2.64	5.33
**AVG**	0.902	5.16	3.87	3.06	2.01	4.18	0.879	5.71	4.25	3.29	2.20	4.61	0.879	5.71	4.24	3.29	2.20	4.60

R^2: R^2 score; $RMSE$: root-mean-squared error; MAE: mean absolute error; $MdAE$: median absolute error; $MAPE$: mean absolute percentage error; ^{90}PAE: 90th percentile of percentage absolute errors. $train$: training set; cal: calibration set; val: validation set.

Table S4. Point-estimation performance metrics for KerasECFP across the METLIN-CCS 25 5x5 cross-validation folds and their arithmetic mean.

Fold	R^2_{train}	$RMSE_{train}$	MAE_{train}	$MdAE_{train}$	$MAPE_{train}$	$^{90}PAE_{train}$	R^2_{cal}	$RMSE_{cal}$	MAE_{cal}	$MdAE_{cal}$	$MAPE_{cal}$	$^{90}PAE_{cal}$	R^2_{val}	$RMSE_{val}$	MAE_{val}	$MdAE_{val}$	$MAPE_{val}$	$^{90}PAE_{val}$
1	0.896	5.30	3.95	3.06	2.06	4.36	0.867	5.97	4.40	3.38	2.29	4.91	0.878	5.79	4.31	3.31	2.24	4.82
2	0.922	4.59	3.38	2.60	1.75	3.65	0.882	5.65	4.12	3.11	2.14	4.50	0.879	5.70	4.18	3.17	2.15	4.59
3	0.896	5.30	3.92	3.01	2.04	4.25	0.874	5.81	4.31	3.32	2.23	4.71	0.875	5.79	4.29	3.29	2.23	4.69
4	0.907	5.03	3.73	2.88	1.93	4.04	0.877	5.75	4.23	3.19	2.17	4.58	0.875	5.79	4.27	3.31	2.20	4.57
5	0.911	4.89	3.62	2.79	1.87	3.92	0.888	5.59	4.12	3.17	2.12	4.47	0.878	5.77	4.21	3.20	2.18	4.58
6	0.909	4.97	3.66	2.81	1.89	3.95	0.879	5.67	4.19	3.22	2.17	4.60	0.884	5.64	4.18	3.19	2.17	4.61
7	0.904	5.10	3.79	2.92	1.98	4.20	0.872	5.86	4.35	3.40	2.27	4.81	0.871	5.87	4.34	3.31	2.26	4.81
8	0.909	4.96	3.66	2.79	1.90	4.00	0.879	5.67	4.21	3.25	2.17	4.60	0.882	5.63	4.10	3.13	2.13	4.45
9	0.913	4.85	3.59	2.76	1.86	3.92	0.883	5.69	4.17	3.12	2.16	4.55	0.879	5.73	4.22	3.23	2.19	4.62
10	0.896	5.30	3.98	3.12	2.08	4.36	0.876	5.80	4.32	3.36	2.25	4.77	0.871	5.94	4.43	3.45	2.32	4.89
11	0.900	5.20	3.85	2.97	2.00	4.23	0.874	5.87	4.31	3.33	2.24	4.75	0.876	5.79	4.29	3.32	2.23	4.71
12	0.909	4.95	3.66	2.81	1.89	3.94	0.881	5.67	4.20	3.22	2.18	4.60	0.880	5.78	4.26	3.25	2.20	4.66
13	0.923	4.57	3.37	2.59	1.75	3.68	0.883	5.63	4.09	3.11	2.13	4.49	0.880	5.65	4.16	3.16	2.16	4.58
14	0.896	5.32	3.96	3.05	2.04	4.25	0.873	5.86	4.37	3.33	2.25	4.68	0.864	6.02	4.41	3.41	2.27	4.72
15	0.900	5.19	3.90	3.07	2.04	4.29	0.867	6.10	4.45	3.42	2.33	4.85	0.875	5.81	4.37	3.42	2.28	4.80
16	0.918	4.70	3.50	2.71	1.81	3.72	0.881	5.70	4.17	3.17	2.15	4.50	0.877	5.78	4.17	3.15	2.15	4.57
17	0.918	4.73	3.49	2.70	1.81	3.76	0.879	5.68	4.16	3.14	2.15	4.56	0.880	5.66	4.15	3.11	2.14	4.51
18	0.908	4.99	3.68	2.84	1.91	4.02	0.884	5.57	4.19	3.20	2.17	4.64	0.878	5.76	4.28	3.32	2.22	4.63
19	0.905	5.06	3.79	2.99	1.96	4.02	0.872	5.78	4.32	3.37	2.23	4.67	0.878	5.78	4.34	3.40	2.24	4.64
20	0.911	4.90	3.63	2.78	1.88	3.91	0.872	5.85	4.27	3.18	2.21	4.72	0.881	5.66	4.19	3.22	2.16	4.48
21	0.912	4.89	3.60	2.76	1.87	3.92	0.873	5.81	4.20	3.26	2.20	4.53	0.882	5.65	4.15	3.14	2.15	4.52
22	0.914	4.83	3.56	2.73	1.85	3.92	0.882	5.69	4.20	3.17	2.18	4.58	0.885	5.58	4.09	3.11	2.12	4.44
23	0.909	4.97	3.68	2.84	1.92	4.04	0.879	5.71	4.20	3.17	2.18	4.55	0.876	5.81	4.31	3.31	2.25	4.75
24	0.911	4.93	3.68	2.87	1.92	4.03	0.871	5.88	4.36	3.40	2.27	4.77	0.879	5.69	4.24	3.28	2.21	4.66
25	0.896	5.29	4.01	3.21	2.06	4.18	0.872	5.94	4.52	3.60	2.32	4.77	0.862	6.12	4.57	3.56	2.35	4.82
**AVG**	0.908	4.99	3.71	2.87	1.92	4.02	0.877	5.77	4.26	3.26	2.21	4.65	0.877	5.77	4.26	3.27	2.21	4.65

R^2: R^2 score; $RMSE$: root-mean-squared error; MAE: mean absolute error; $MdAE$: median absolute error; $MAPE$: mean absolute percentage error; ^{90}PAE: 90th percentile of percentage absolute errors. $train$: training set; cal: calibration set; val: validation set.

Table S5. Point-estimation performance metrics for LinMZ across the METLIN-CCS 25 5x5 cross-validation folds and their arithmetic mean.

Fold	R^2_{train}	$RMSE_{train}$	MAE_{train}	$MdAE_{train}$	$MAPE_{train}$	$^{90}PAE_{train}$	R^2_{cal}	$RMSE_{cal}$	MAE_{cal}	$MdAE_{cal}$	$MAPE_{cal}$	$^{90}PAE_{cal}$	R^2_{val}	$RMSE_{val}$	MAE_{val}	$MdAE_{val}$	$MAPE_{val}$	$^{90}PAE_{val}$
1	0.764	7.99	6.24	5.18	0.03	6.69	0.759	8.05	6.26	5.22	0.03	6.68	0.776	7.85	6.14	5.07	0.03	6.63
2	0.767	7.95	6.21	5.14	0.03	6.66	0.777	7.76	6.06	5.09	0.03	6.56	0.756	8.08	6.33	5.30	0.03	6.81
3	0.766	7.98	6.24	5.16	0.03	6.69	0.765	7.93	6.18	5.12	0.03	6.66	0.766	7.92	6.18	5.17	0.03	6.63
4	0.768	7.94	6.20	5.15	0.03	6.65	0.758	8.07	6.30	5.23	0.03	6.77	0.761	8.02	6.28	5.19	0.03	6.77
5	0.763	7.98	6.24	5.19	0.03	6.70	0.779	7.84	6.15	5.09	0.03	6.64	0.769	7.95	6.18	5.09	0.03	6.61
6	0.764	7.98	6.23	5.18	0.03	6.68	0.762	7.95	6.23	5.14	0.03	6.58	0.771	7.92	6.19	5.12	0.03	6.71
7	0.766	7.97	6.22	5.15	0.03	6.69	0.772	7.83	6.16	5.11	0.03	6.54	0.761	8.00	6.28	5.22	0.03	6.69
8	0.768	7.94	6.20	5.13	0.03	6.67	0.755	8.08	6.30	5.18	0.03	6.70	0.760	8.02	6.28	5.25	0.03	6.69
9	0.764	7.98	6.24	5.19	0.03	6.69	0.769	7.99	6.30	5.25	0.03	6.71	0.769	7.89	6.13	5.02	0.03	6.58
10	0.765	7.96	6.22	5.14	0.03	6.67	0.766	7.96	6.22	5.27	0.03	6.67	0.766	7.99	6.24	5.18	0.03	6.73
11	0.765	7.96	6.22	5.16	0.03	6.68	0.769	7.95	6.21	5.08	0.03	6.63	0.765	7.98	6.24	5.21	0.03	6.69
12	0.764	7.97	6.22	5.17	0.03	6.67	0.771	7.87	6.14	5.02	0.03	6.60	0.770	7.99	6.26	5.20	0.03	6.73
13	0.766	7.97	6.23	5.16	0.03	6.70	0.771	7.87	6.17	5.09	0.03	6.65	0.760	7.98	6.24	5.21	0.03	6.66
14	0.766	7.96	6.23	5.18	0.03	6.66	0.766	7.96	6.22	5.11	0.03	6.73	0.762	7.98	6.21	5.12	0.03	6.70
15	0.765	7.97	6.23	5.19	0.03	6.69	0.763	8.14	6.28	5.18	0.03	6.63	0.770	7.89	6.16	5.07	0.03	6.63
16	0.767	7.93	6.22	5.18	0.03	6.66	0.761	8.08	6.26	5.09	0.03	6.78	0.762	8.02	6.24	5.13	0.03	6.75
17	0.766	7.97	6.23	5.15	0.03	6.69	0.764	7.95	6.24	5.15	0.03	6.60	0.764	7.94	6.21	5.21	0.03	6.65
18	0.765	7.98	6.23	5.17	0.03	6.69	0.764	7.96	6.26	5.22	0.03	6.71	0.770	7.92	6.19	5.10	0.03	6.60
19	0.766	7.97	6.22	5.16	0.03	6.67	0.762	7.89	6.21	5.17	0.03	6.56	0.767	7.98	6.24	5.16	0.03	6.74
20	0.766	7.97	6.22	5.14	0.03	6.68	0.767	7.90	6.20	5.20	0.03	6.66	0.765	7.96	6.24	5.21	0.03	6.67
21	0.765	7.99	6.23	5.17	0.03	6.69	0.756	8.03	6.30	5.23	0.03	6.72	0.773	7.85	6.16	5.10	0.03	6.63
22	0.765	7.97	6.22	5.16	0.03	6.68	0.766	8.02	6.29	5.21	0.03	6.78	0.767	7.94	6.21	5.16	0.03	6.63
23	0.767	7.94	6.20	5.13	0.03	6.65	0.773	7.82	6.12	5.15	0.03	6.67	0.759	8.11	6.34	5.31	0.03	6.80
24	0.766	7.98	6.24	5.20	0.03	6.69	0.766	7.92	6.16	4.96	0.03	6.66	0.764	7.94	6.18	5.10	0.03	6.65
25	0.764	7.98	6.23	5.18	0.03	6.68	0.781	7.79	6.15	5.13	0.03	6.60	0.765	7.99	6.23	5.14	0.03	6.70
AVG	0.766	7.97	6.22	5.16	0.03	6.68	0.766	7.94	6.21	5.15	0.03	6.66	0.766	7.96	6.22	5.16	0.03	6.68

R^2: R^2 score; $RMSE$: root-mean-squared error; MAE: mean absolute error; $MdAE$: median absolute error; $MAPE$: mean absolute percentage error; ^{90}PAE: 90th percentile of percentage absolute errors. $train$: training set; cal: calibration set; val: validation set.

Mondrian Taxonomies

Point Estimates

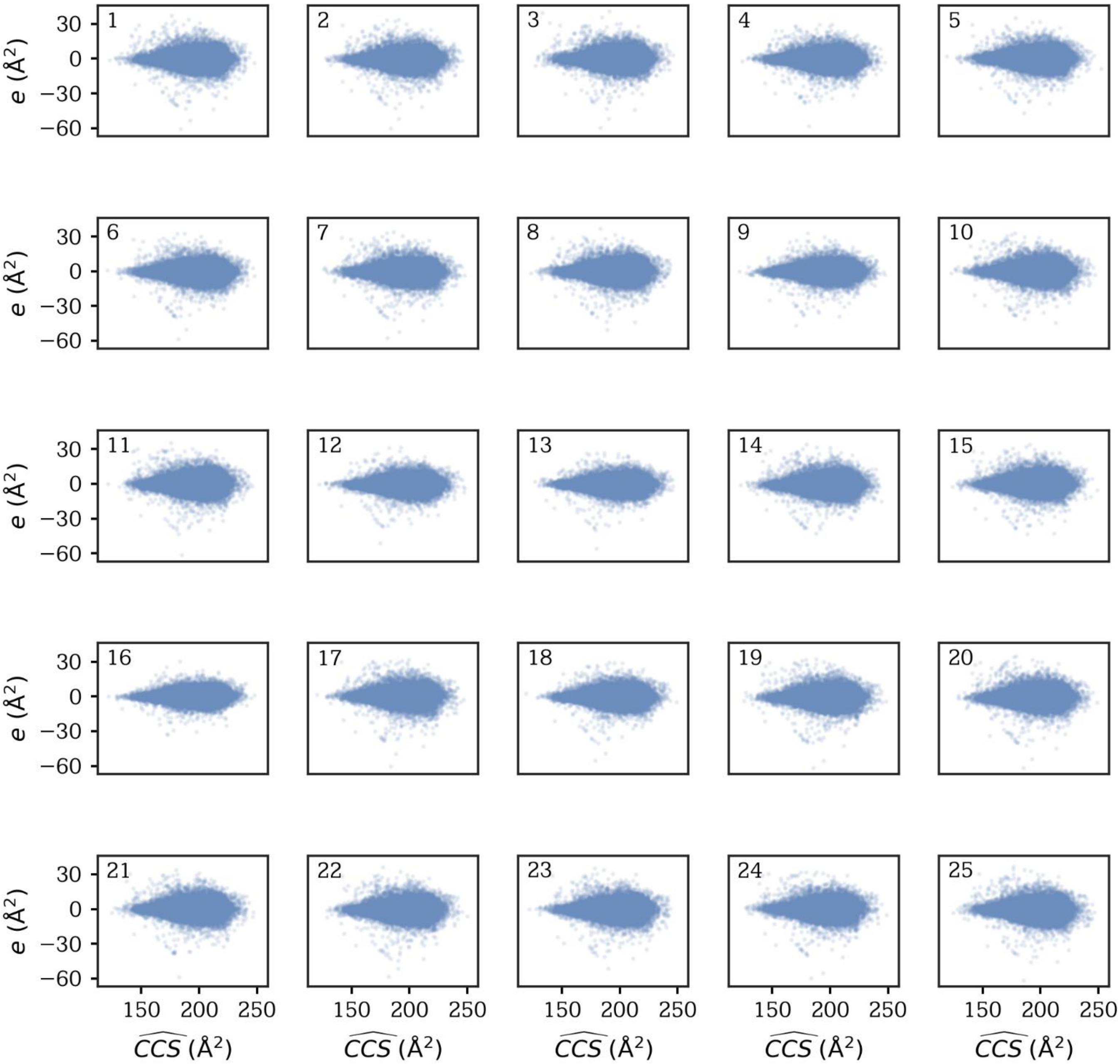

Figure S7. Errors vs. CCS point estimates correlation plots in the training set of each of the METLIN-CCS 25 5x5 cross-validation folds for GraphCCS.

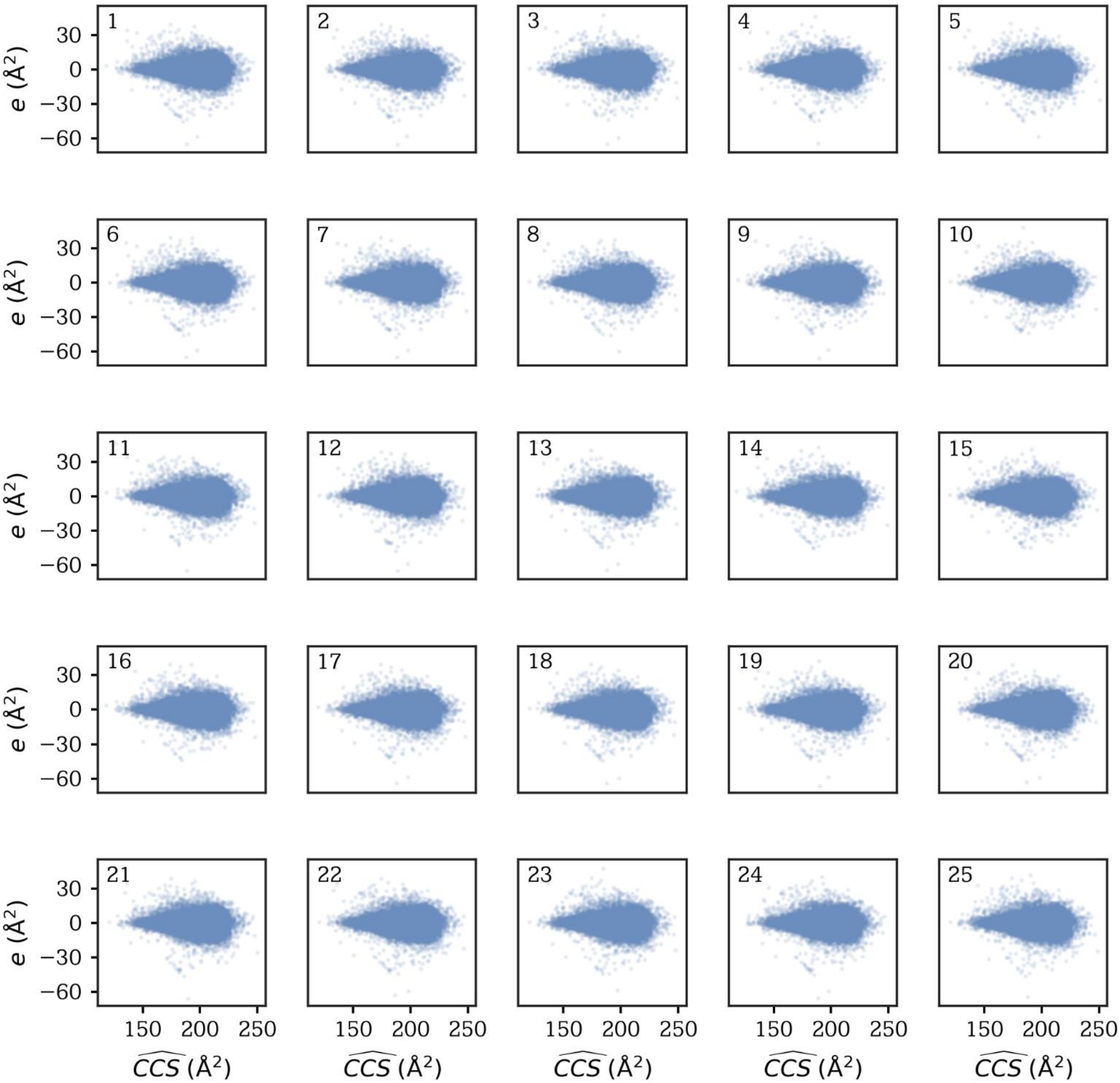

Figure S8. Errors vs. CCS point estimates correlation plots in the training set of each of the METLIN-CCS 25 5x5 cross-validation folds for CCSP2.0.

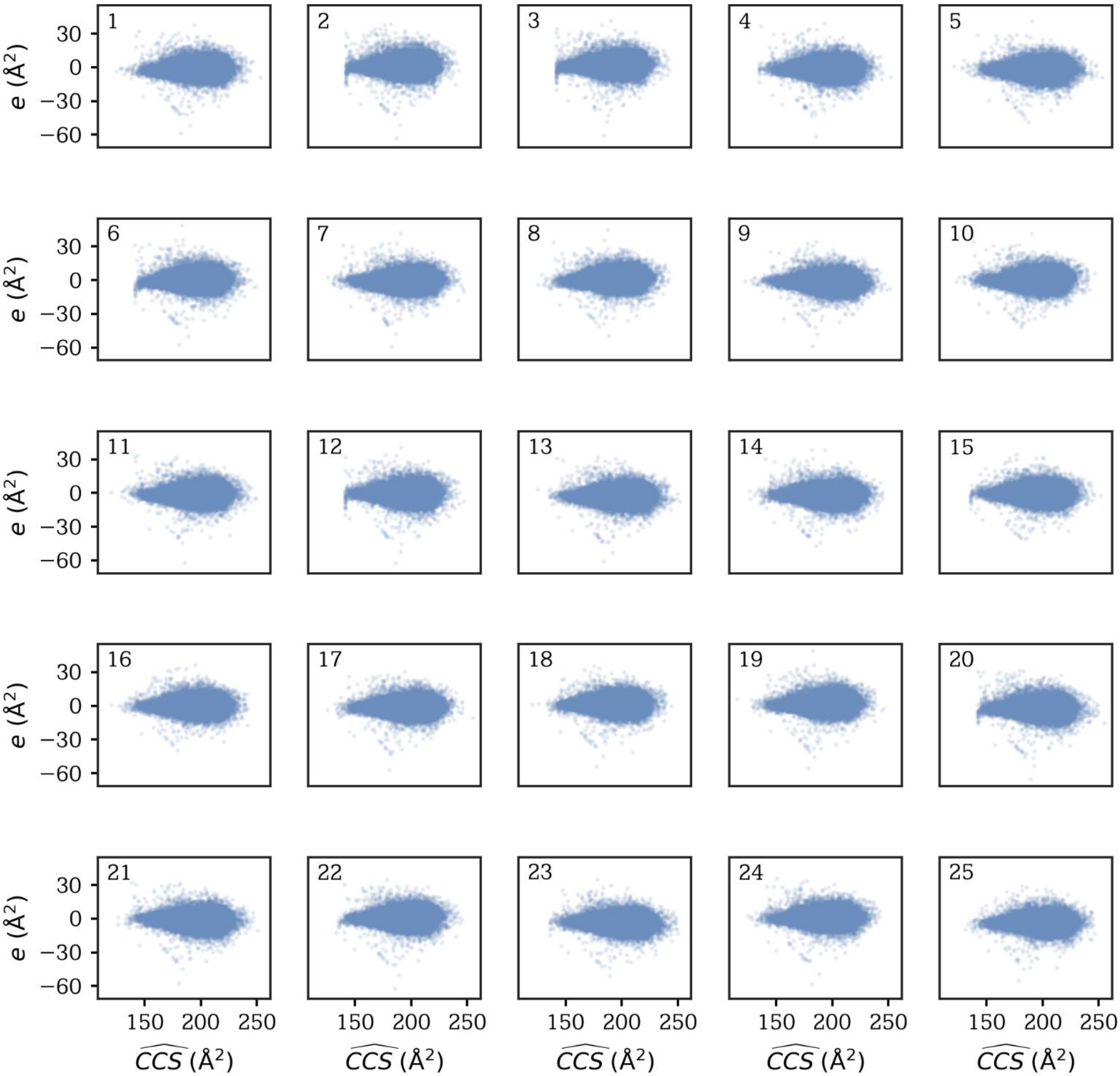

Figure S9. Errors vs. CCS point estimates correlation plots in the training set of each of the METLIN-CCS 25 5x5 cross-validation folds for SigmaCCS.

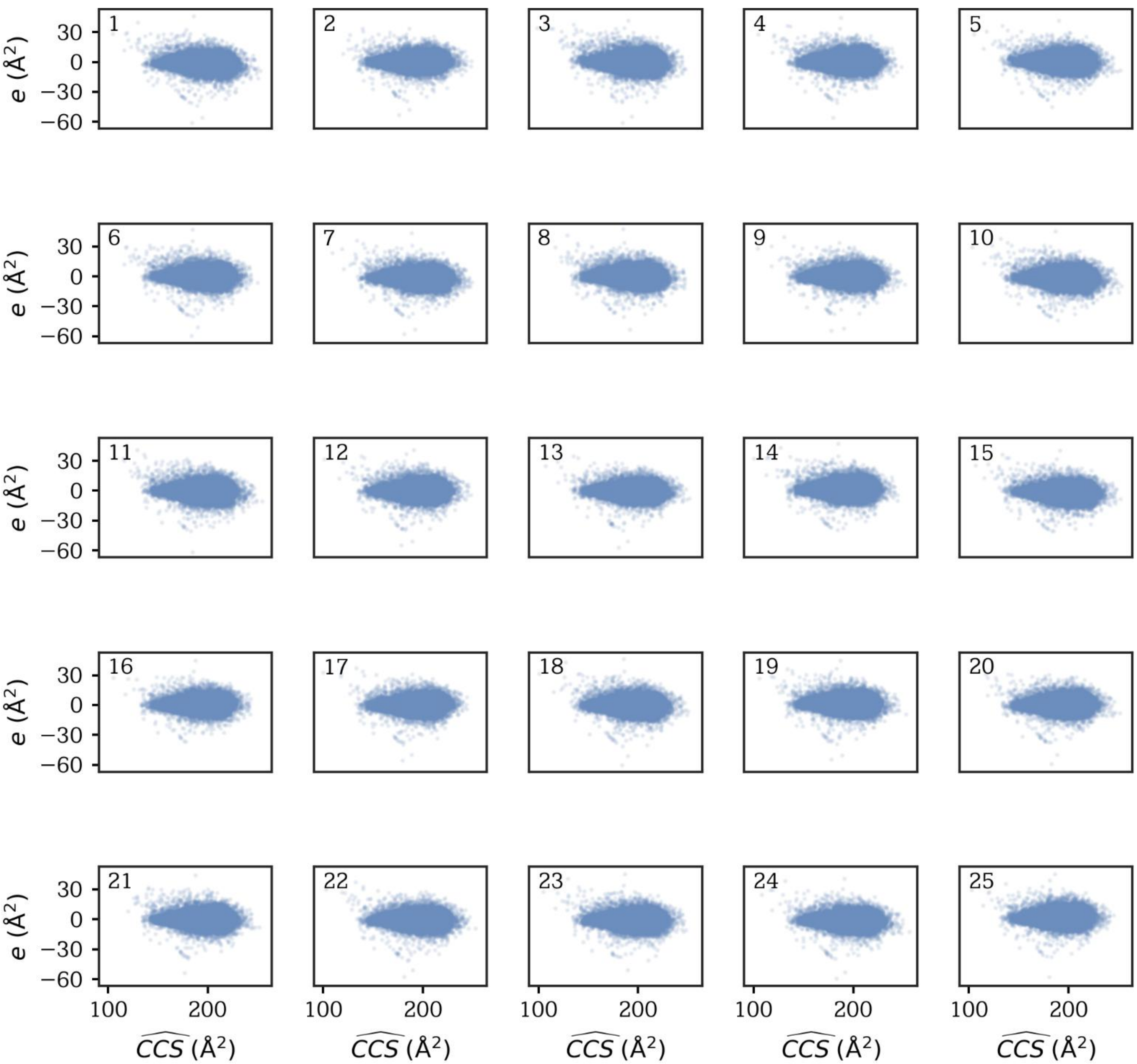

Figure S10. Errors vs. CCS point estimates correlation plots in the training set of each of the METLIN-CCS 25 5x5 cross-validation folds for KerasECFP.

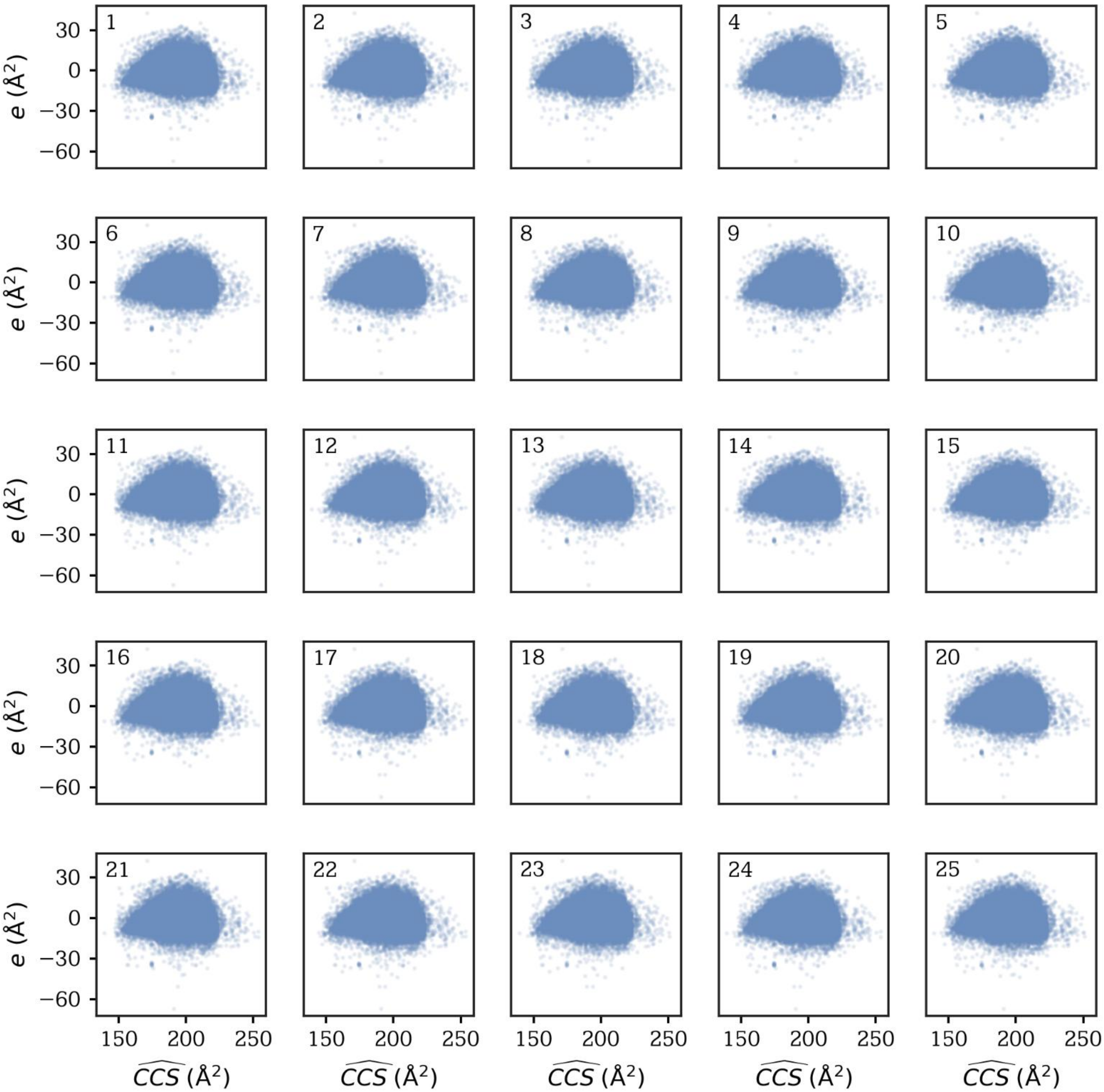

Figure S11. Errors vs. CCS point estimates correlation plots in the training set of each of the METLIN-CCS 25 5x5 cross-validation folds for LinMZ.

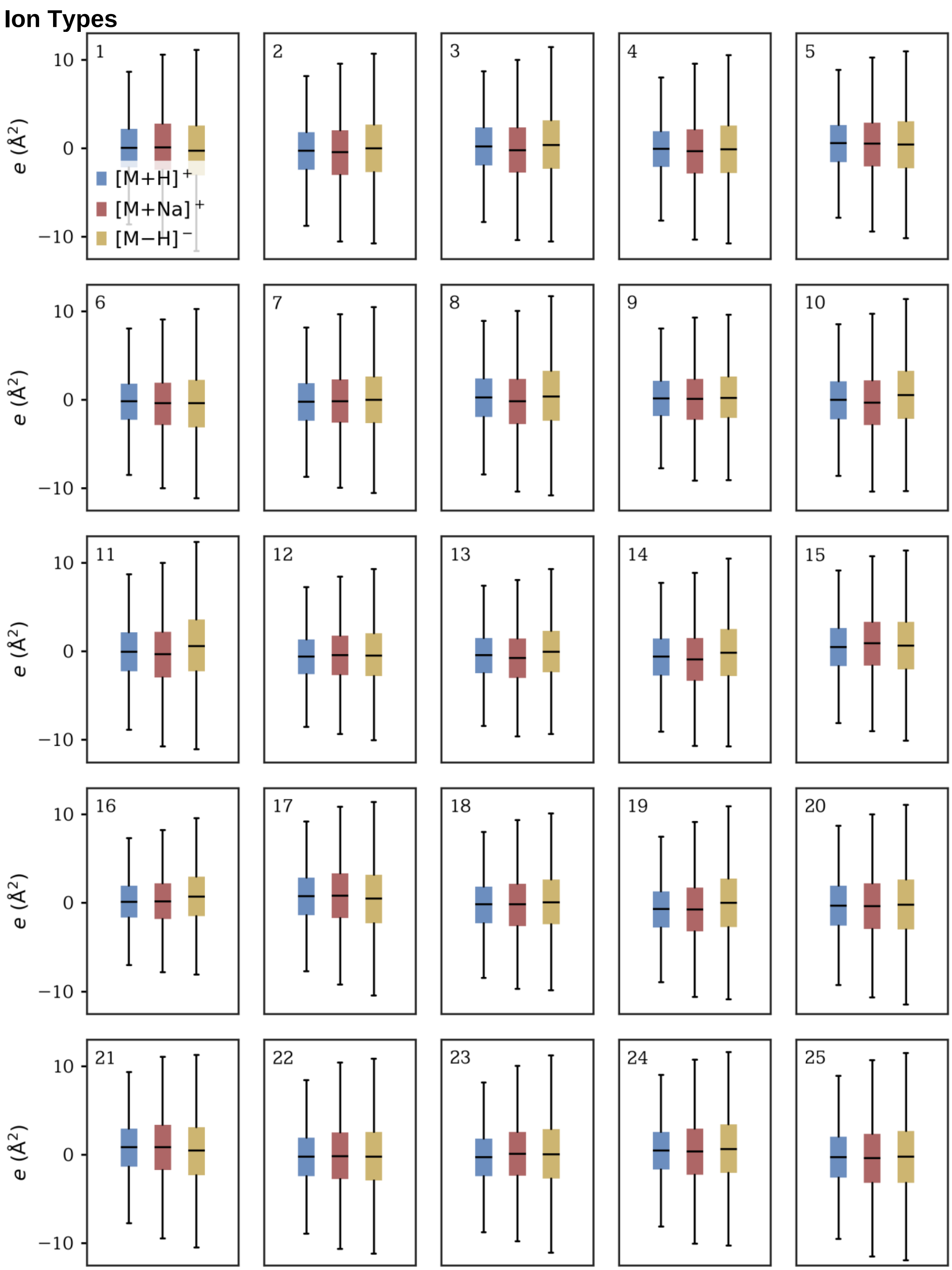

Figure S12. Errors for each ion type in the training set of each of the METLIN-CCS 25 5x5 cross-validation folds for GraphCCS.

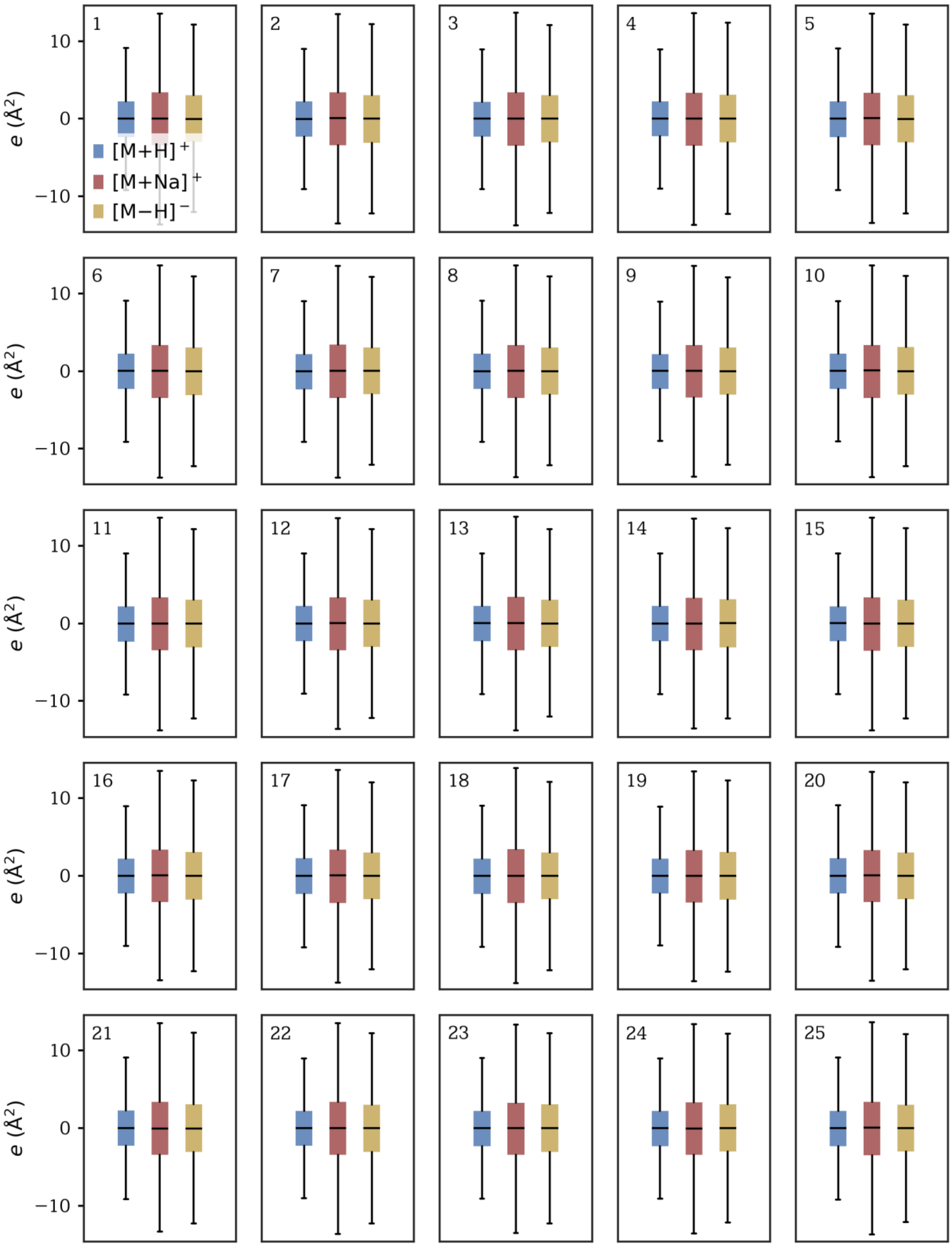

Figure S13. Errors for each ion type in the training set of each of the METLIN-CCS 25 5x5 cross-validation folds for CCSP2.0.

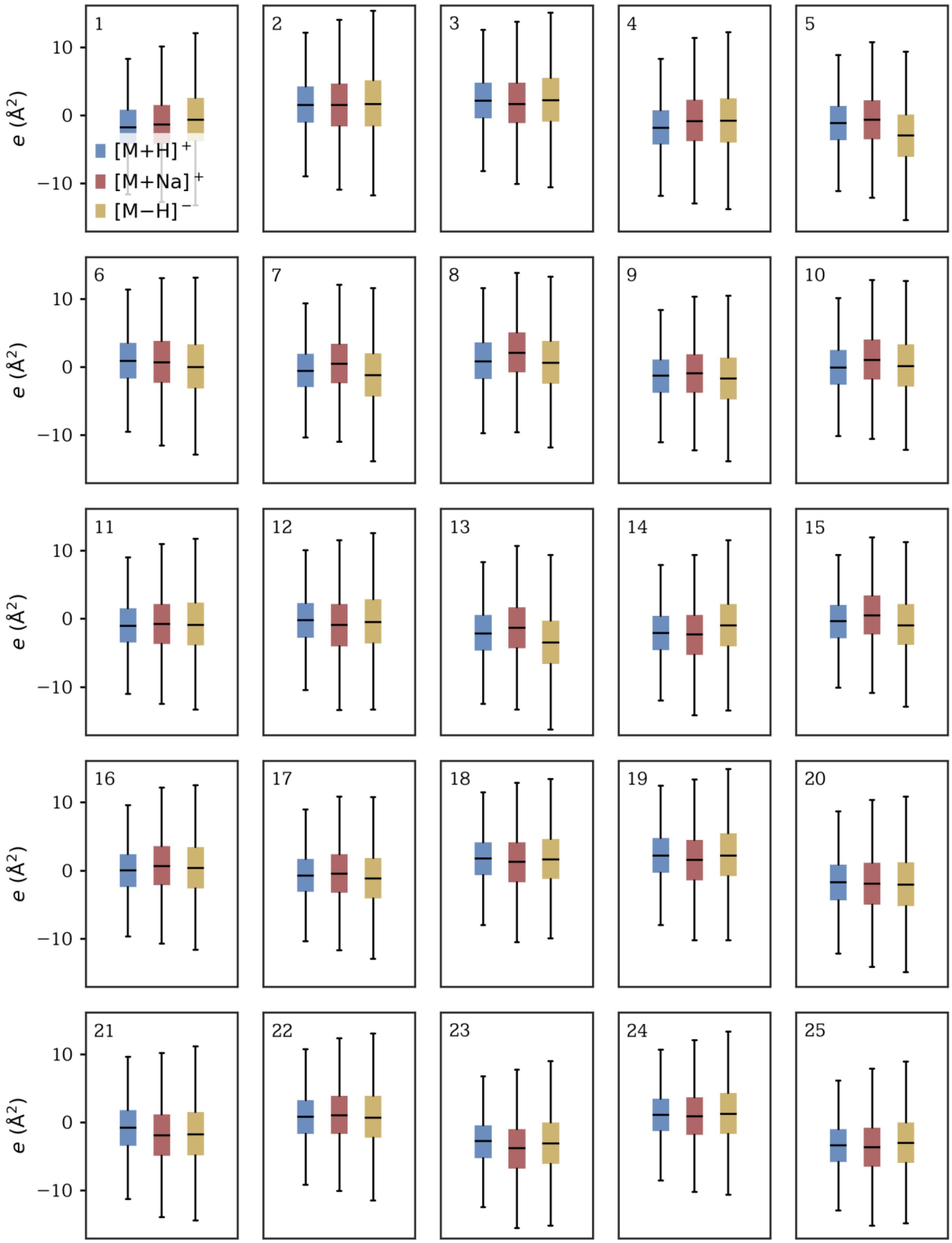

Figure S14. Errors for each ion type in the training set of each of the METLIN-CCS 25 5x5 cross-validation folds for SigmaCCS.

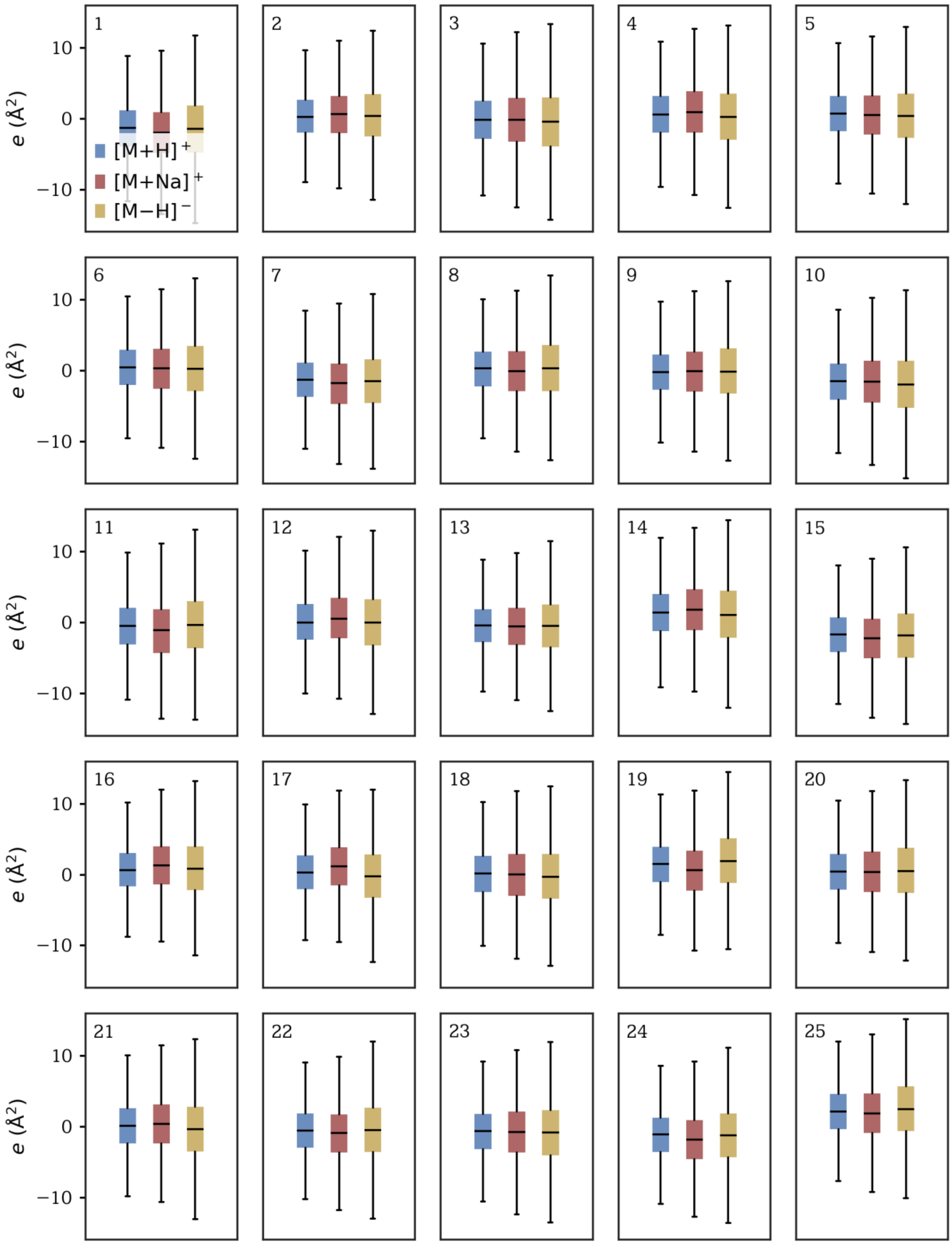

Figure S15. Errors for each ion type in the training set of each of the METLIN-CCS 25 5x5 cross-validation folds for KerasECFP.

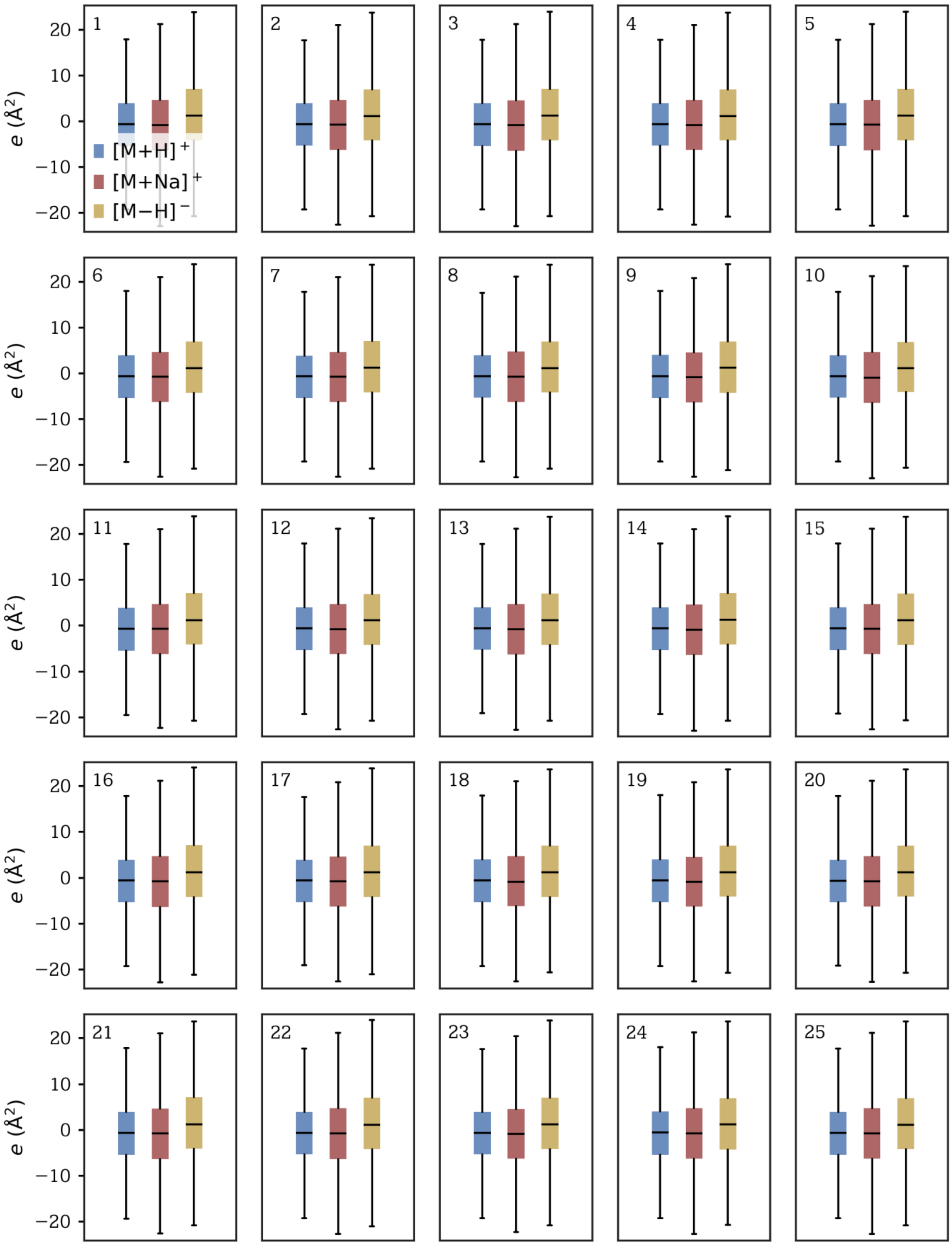

Figure S16. Errors for each ion type in the training set of each of the METLIN-CCS 25 5x5 cross-validation folds for LinMZ.

Error Function

n & k

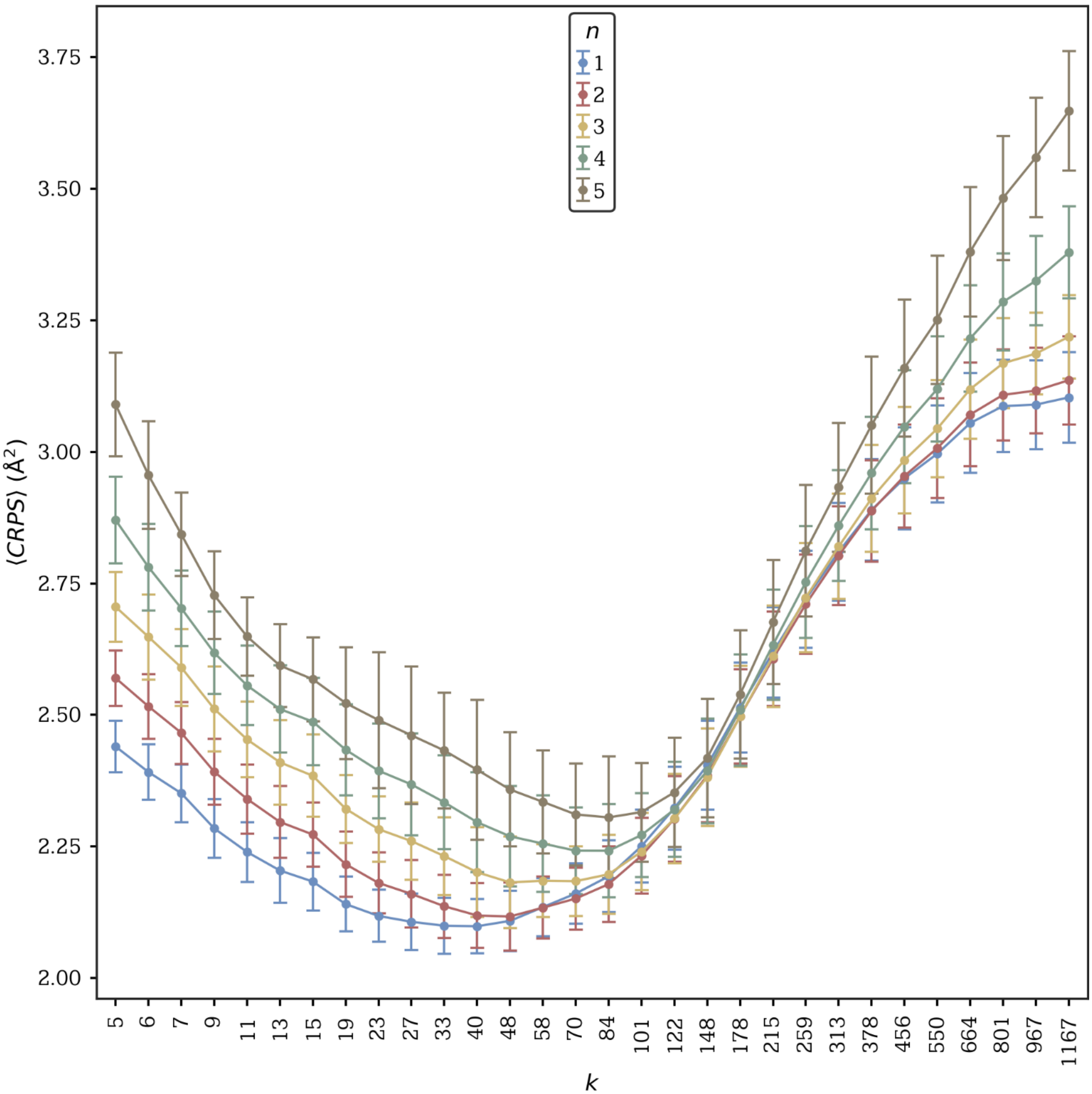

Figure S17. Mean and standard deviation of $\langle CRPS \rangle$ across the METLIN-CCS 25 5x5 cross-validation folds depending on k and n, as in $a\langle MCES \rangle_k^n$, for CCSP2.0.

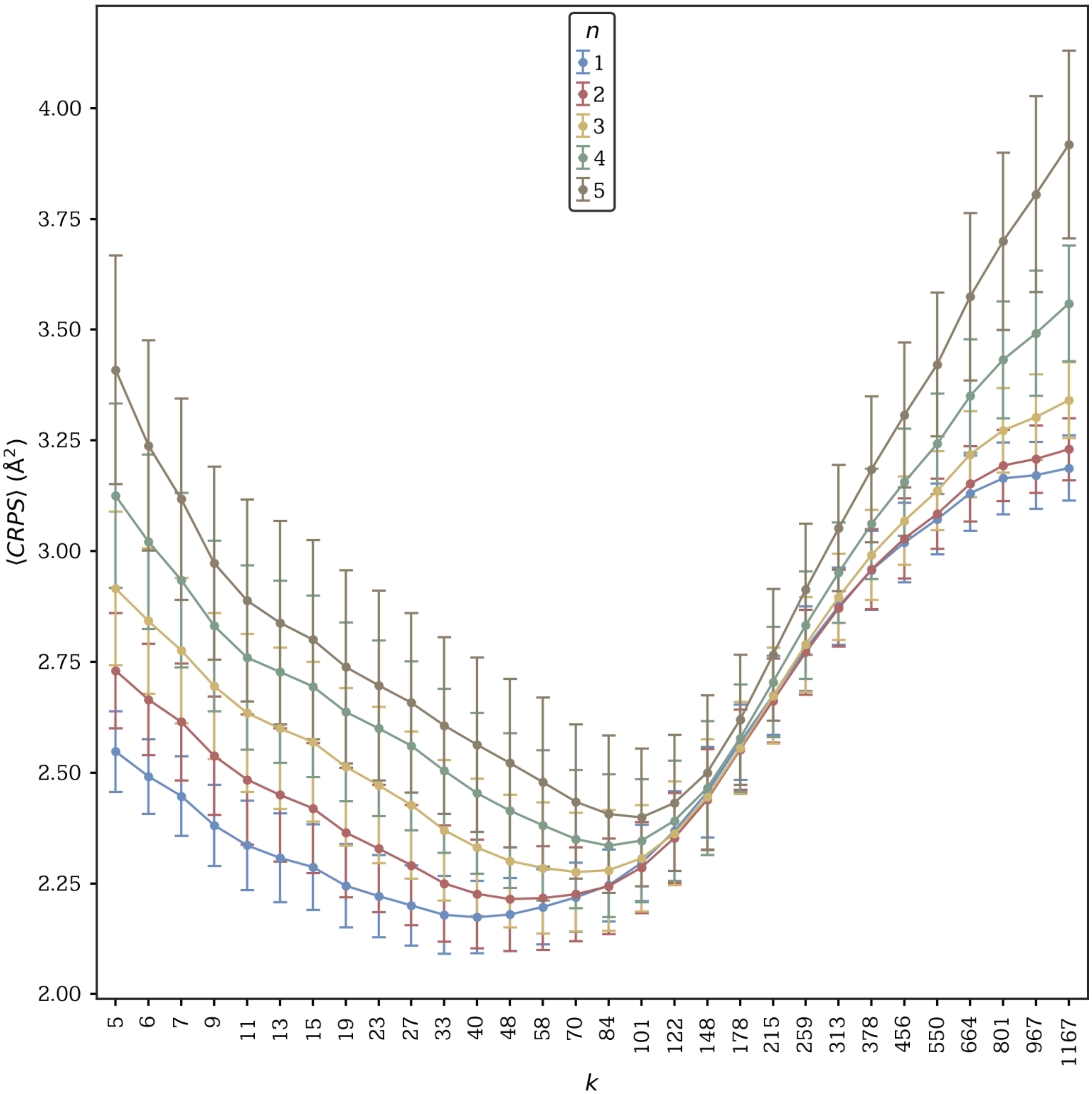

Figure S18. Mean and standard deviation of $\langle CRPS \rangle$ across the METLIN-CCS 25 5x5 cross-validation folds depending on k and n, as in $a\langle MCES \rangle_k^n$, for SigmaCCS.

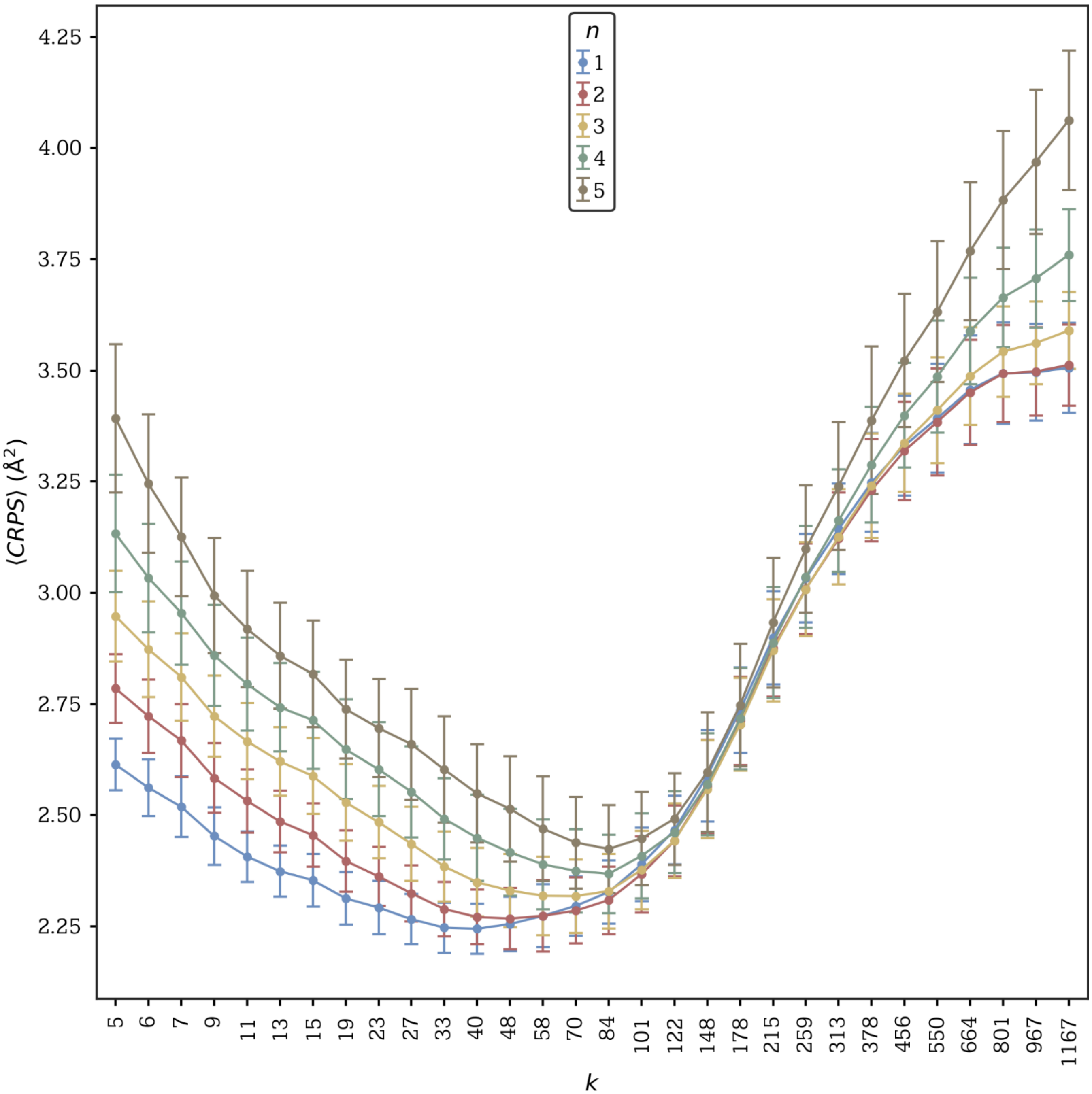

Figure S19. Mean and standard deviation of $\langle CRPS \rangle$ across the METLIN-CCS 25 5x5 cross-validation folds depending on k and n, as in $a\langle MCES \rangle_k^n$, for KerasECFP.

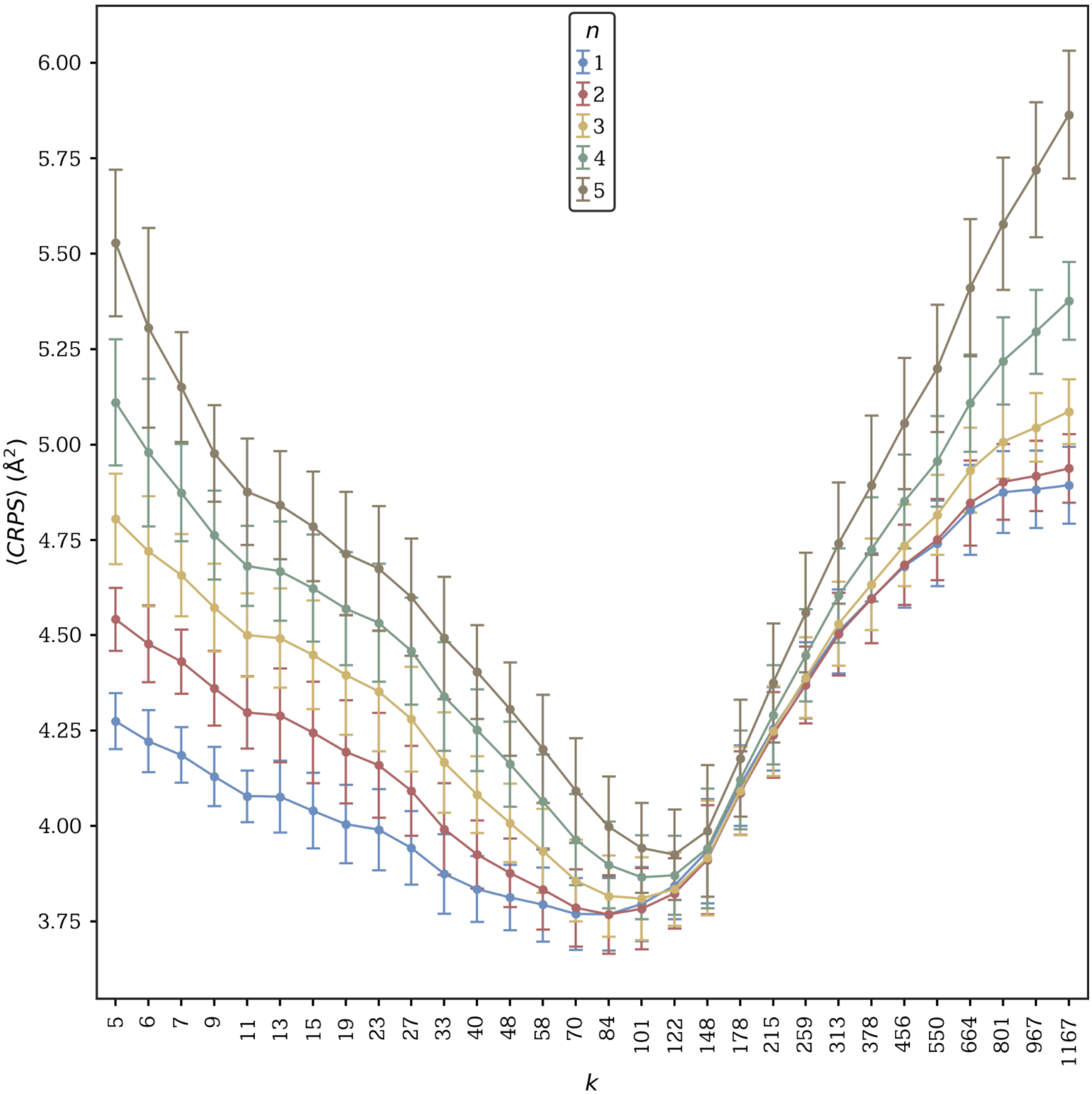

Figure S20. Mean and standard deviation of $\langle CRPS \rangle$ across the METLIN-CCS 25 5x5 cross-validation folds depending on k and n, as in $a\langle MCES \rangle_k^n$, for LinMZ.

Correlation Plots

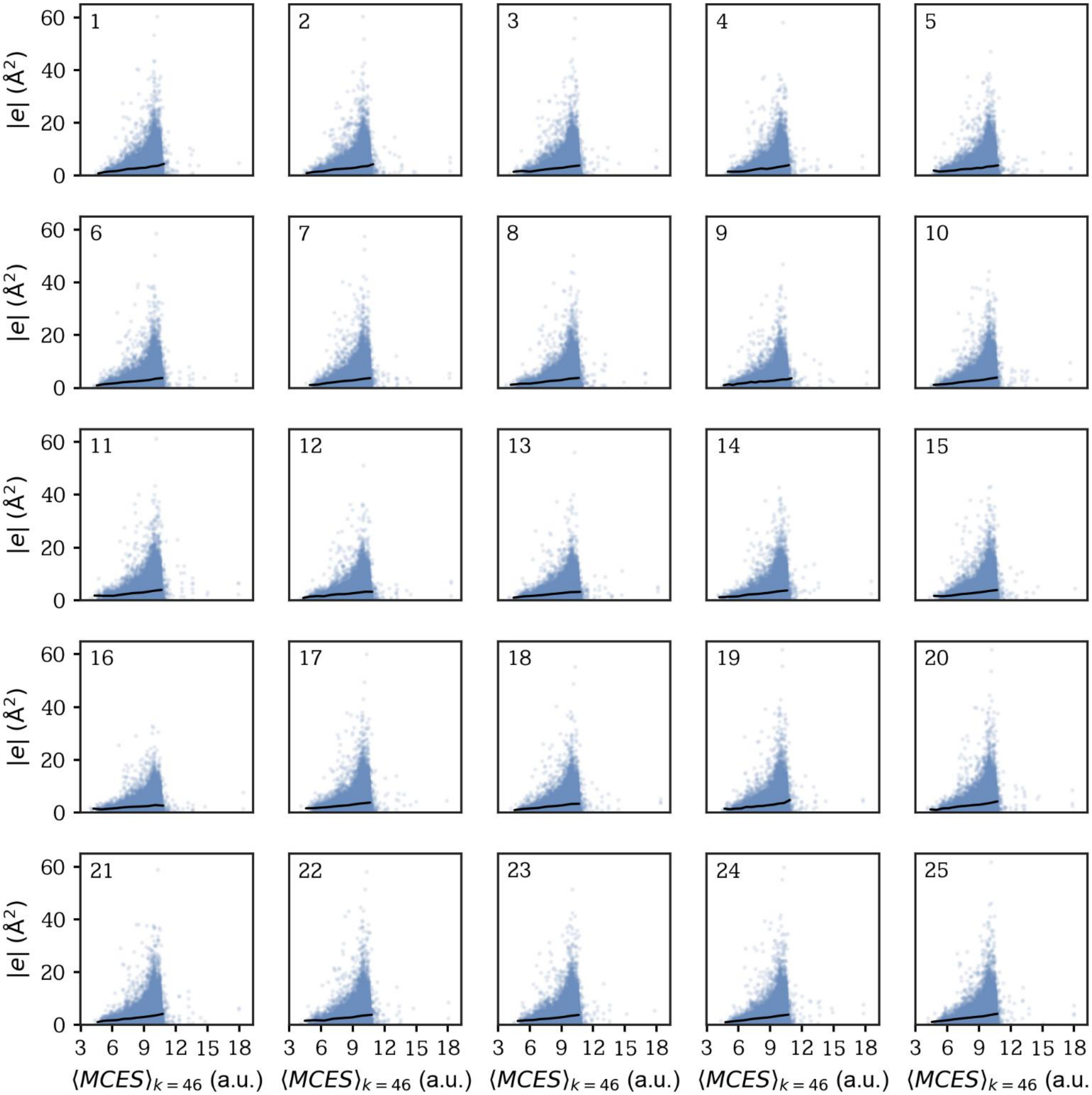

Figure S21. Absolute errors vs. $\langle MCES \rangle_{k=40}$ correlation plots, together with the mean absolute error across the $\langle MCES \rangle_{k=40}$ dimension (black line), in the training set of each of the METLIN-CCS 25 5x5 cross-validation folds for GraphCCS. The approach used to obtain the conditional mean is based on the method for calculating $\langle CRPS \rangle$ with equal-width discretization, explained in the main text in Methods – Evaluation Measures – CRPS. In this case, the minimum sample count per bin was set at 12 resulting in ~10 classes.

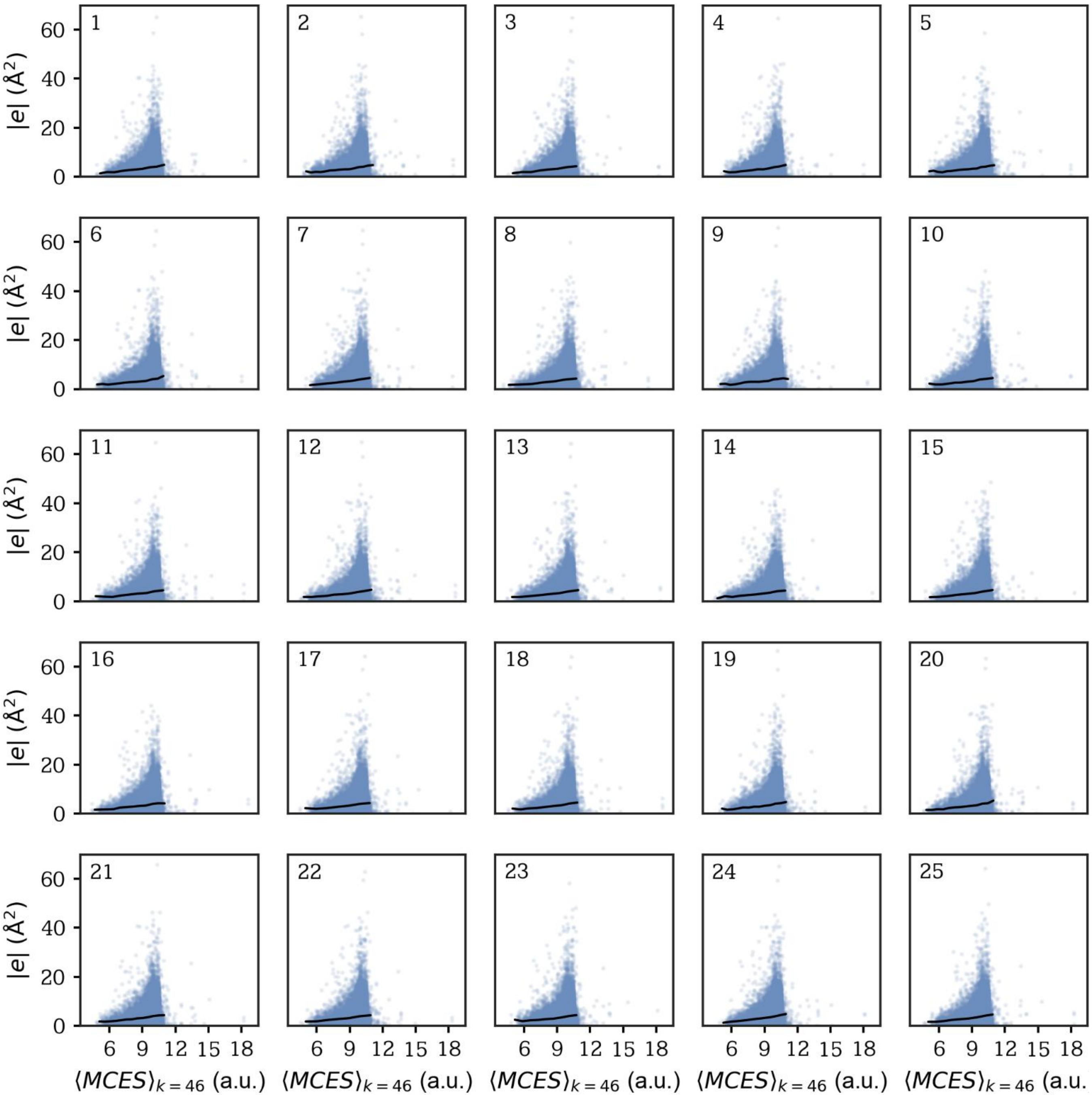

Figure S22. Absolute errors vs. $\langle MCES \rangle_{k=48}$ correlation plots, together with the mean absolute error across the $\langle MCES \rangle_{k=48}$ dimension (black line), in the training set of each of the METLIN-CCS 25 5x5 cross-validation folds for CCSP2.0. The approach used to obtain the conditional mean is based on the method for calculating $\langle CRPS \rangle$ with equal-width discretization, explained in the main text in Methods – Evaluation Measures – CRPS. In this case, the minimum sample count per bin was set at 12 resulting in ~10 classes.

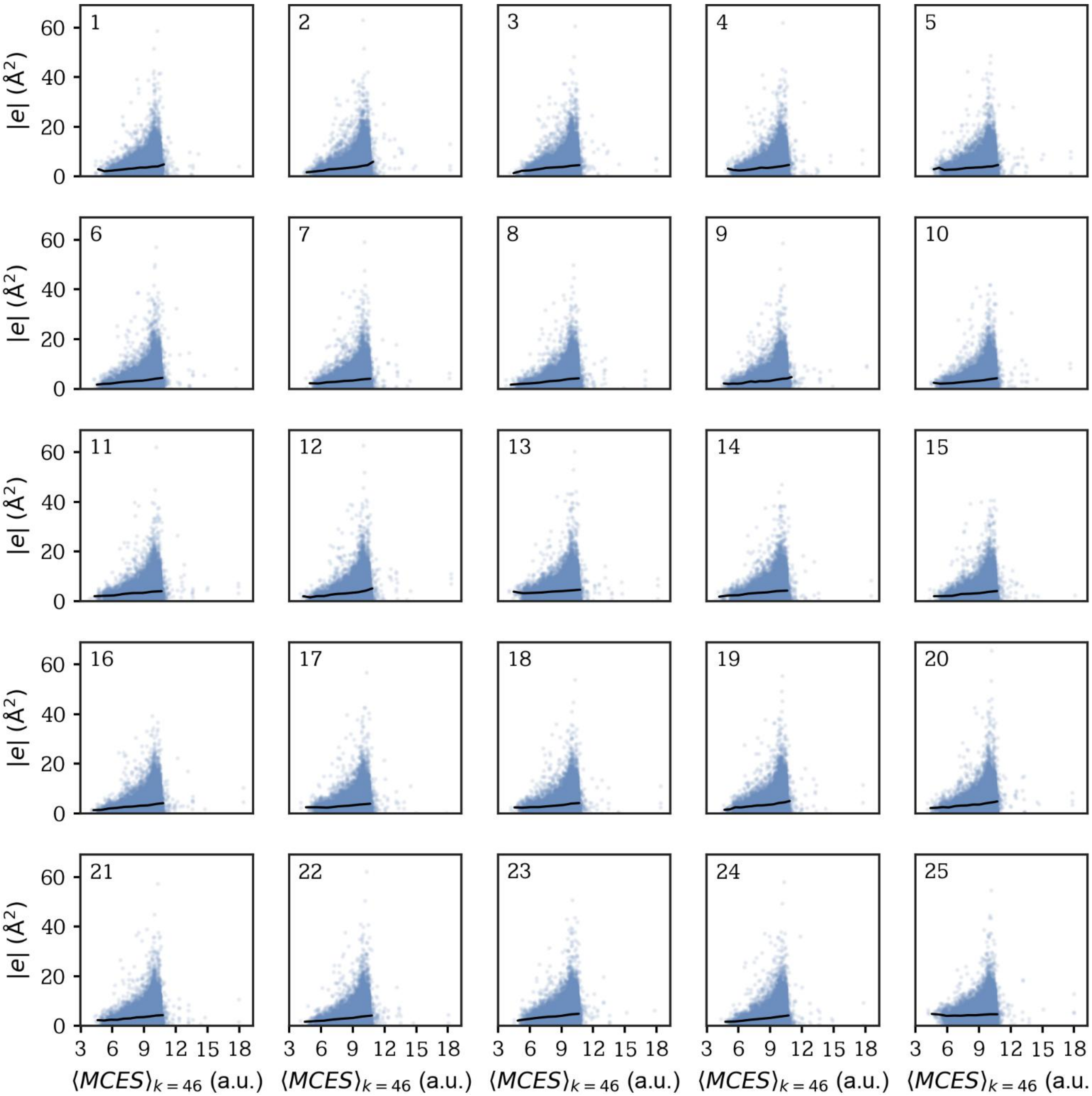

Figure S23. Absolute errors vs. $\langle MCES \rangle_{k=40}$ correlation plots, together with the mean absolute error across the $\langle MCES \rangle_{k=40}$ dimension (black line), in the training set of each of the METLIN-CCS 25 5x5 cross-validation folds for SigmaCCS. The approach used to obtain the conditional mean is based on the method for calculating $\langle CRPS \rangle$ with equal-width discretization, explained in the main text in Methods – Evaluation Measures – CRPS. In this case, the minimum sample count per bin was set at 12 resulting in ~10 classes.

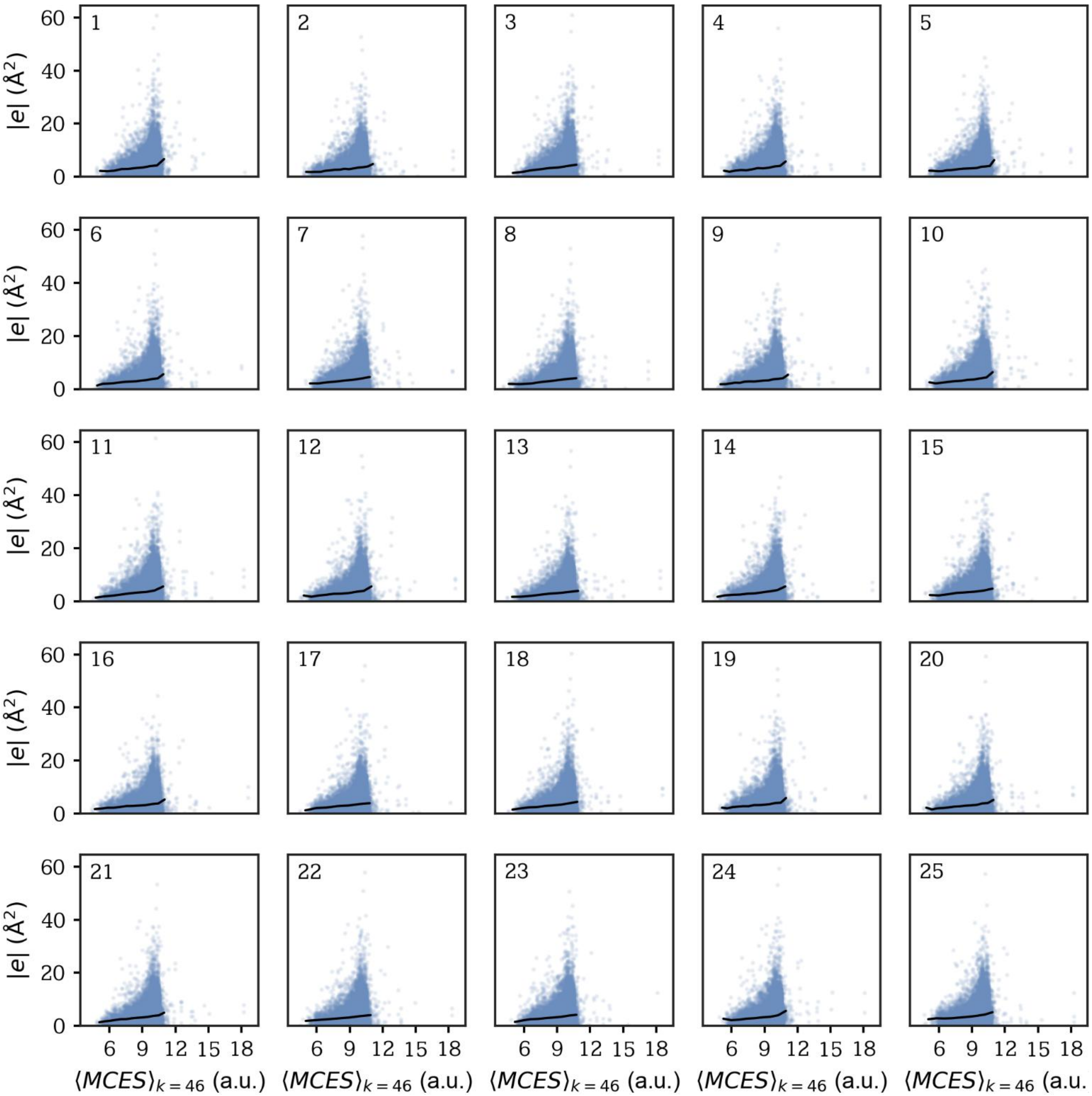

Figure S24. Absolute errors vs. $\langle MCES \rangle_{k=48}$ correlation plots, together with the mean absolute error across the $\langle MCES \rangle_{k=48}$ dimension (black line), in the training set of each of the METLIN-CCS 25 5x5 cross-validation folds for KerasECFP. The approach used to obtain the conditional mean is based on the method for calculating $\langle CRPS \rangle$ with equal-width discretization, explained in the main text in Methods – Evaluation Measures – CRPS. In this case, the minimum sample count per bin was set at 12 resulting in ~10 classes.

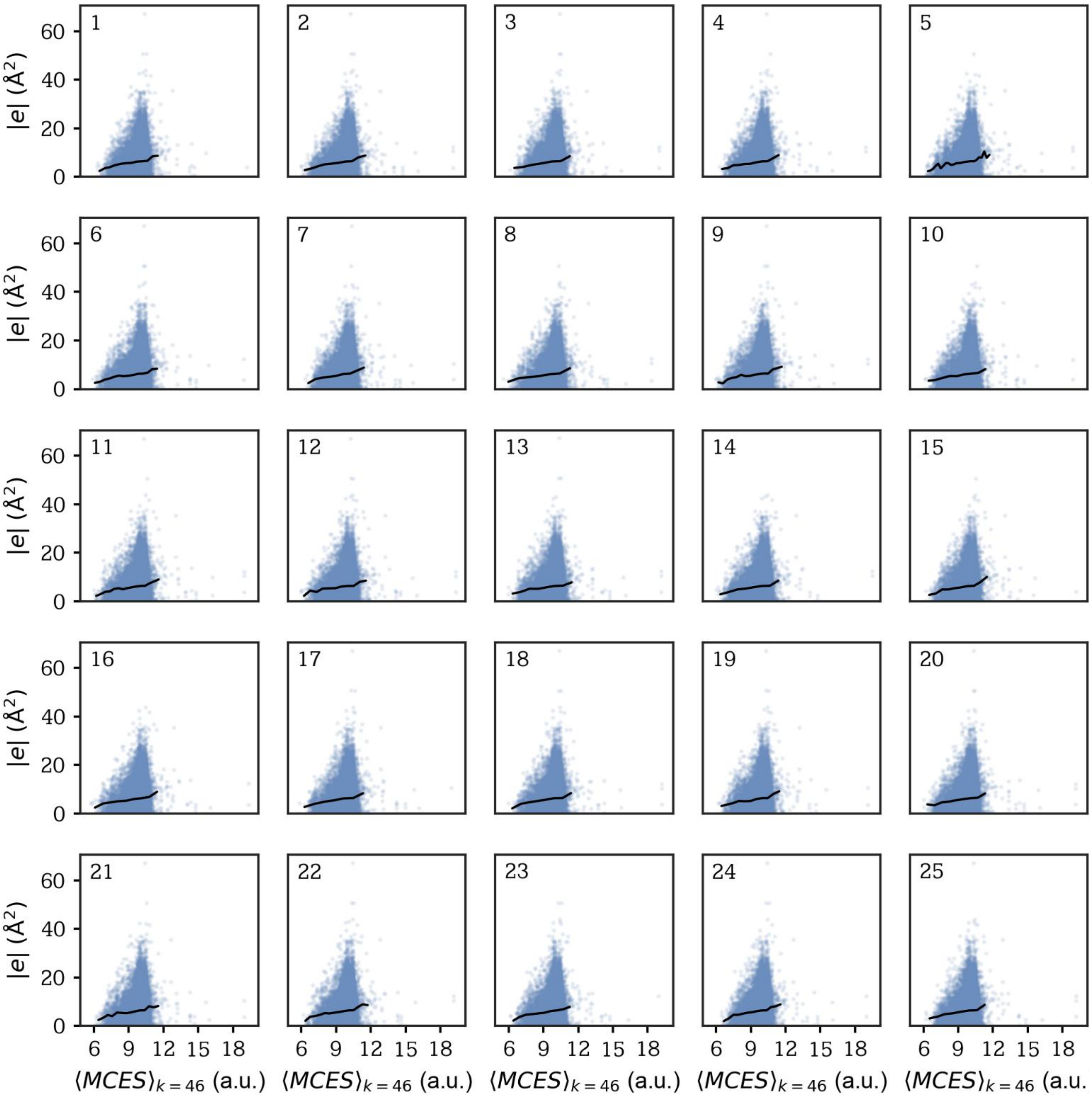

Figure S25. Absolute errors vs. $\langle MCES \rangle_{k=84}$ correlation plots, together with the mean absolute error across the $\langle MCES \rangle_{k=84}$ dimension (black line), in the training set of each of the METLIN-CCS 25 5x5 cross-validation folds for LinMZ. The approach used to obtain the conditional mean is based on the method for calculating $\langle CRPS \rangle$ with equal-width discretization, explained in the main text in Methods – Evaluation Measures – CRPS. In this case, the minimum sample count per bin was set at 12 resulting in ~10 classes.

ANOVA + Tukey's HSD

Levene's test for homogeneity of variances of the performance metrics across conformal predictive systems was statistically significant at 95% confidence for $\langle CRPS \rangle$, but it was not

considered practically significant, as the variance fold difference was 2.5, which is well below 9.[3] Therefore, $\langle CRPS \rangle$ does not practically violate the assumption of homogeneity of variances. On the other hand, it is also the only performance metric with approximately normally distributed values (Figure S26). Consequently, the statistical comparison based on ANOVA + Tukey's HSD is performed only on $\langle CRPS \rangle$.

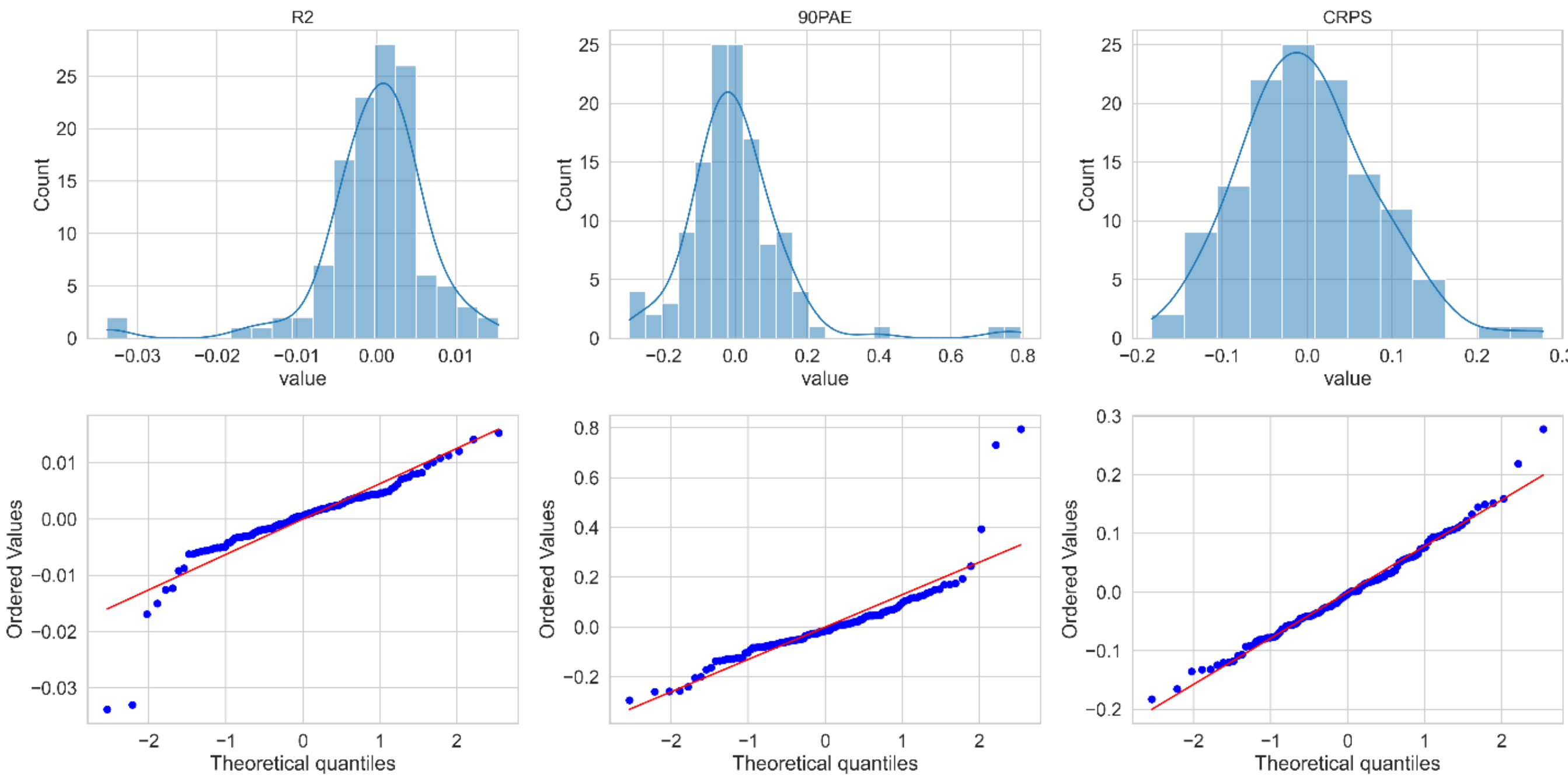

Figure S26. Normality diagnostics for the validation scores R^2, ${}^{90}PAE$ (Å^2) – 90th percentile of percentage absolute errors – and $\langle CRPS \rangle$ (Å^2) – stratified arithmetic mean of CRPSs –. Figure generated with code from Ash *et al.*[3], DOI: 10.1021/acs.jcim.5c01609.

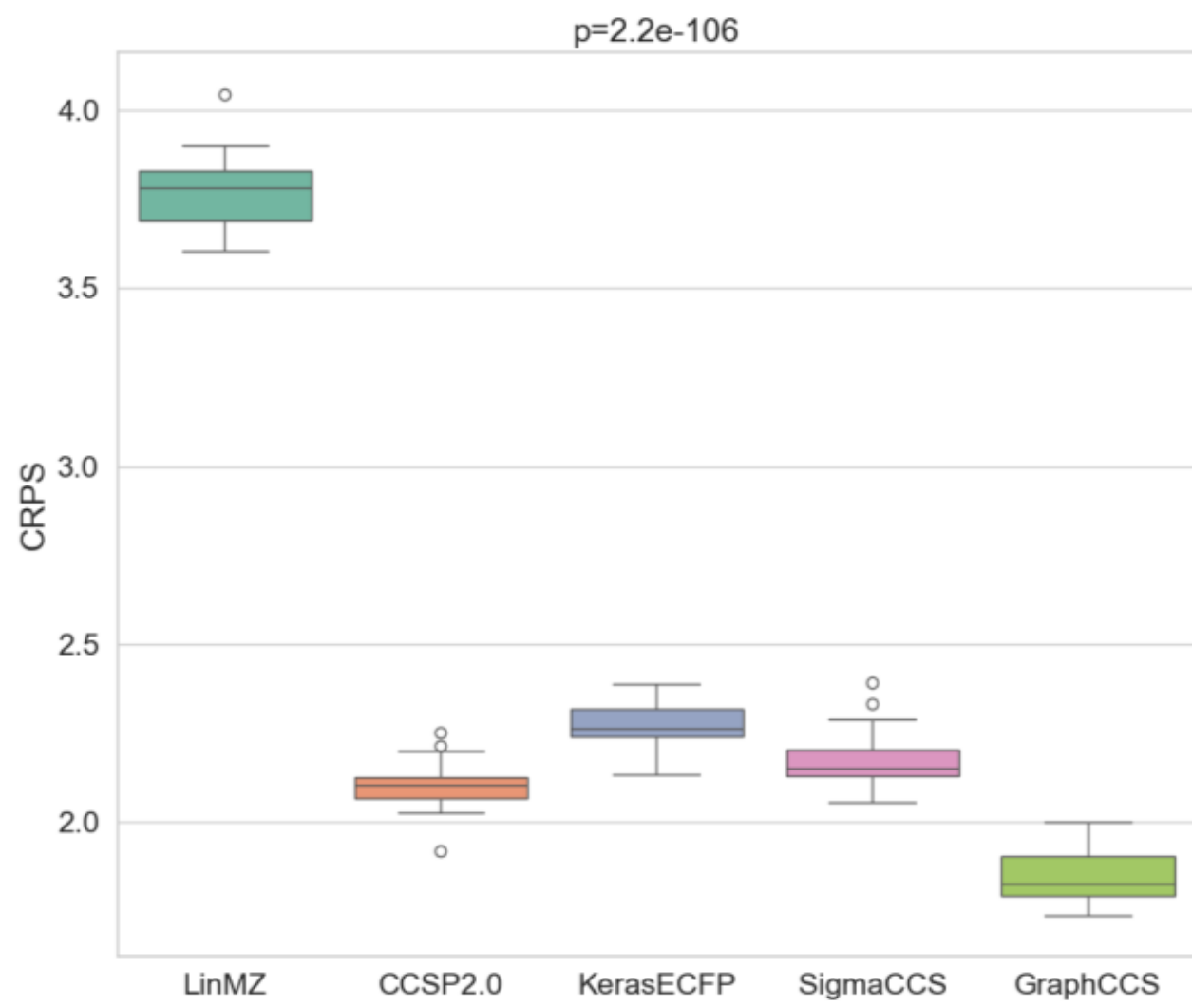

Figure S27. Result for the repeated measures ANOVA of $\langle CRPS \rangle$ (Å^2) – stratified arithmetic mean of CRPSs –. Figure generated with code from Ash *et al.*[3], DOI: 10.1021/acs.jcim.5c01609.

Recall

Empirical recall, here denoted C, was evaluated for all conformal predictive systems across all METLIN-CCS 25 5x5 cross-validation folds to ensure that a valid system had been constructed (Table S6). Additionally, the Mondrian-taxonomy-stratified empirical recall, denoted as C', was calculated.

Table S6. Empirical recall checks, denoted as $C_i^{(\prime)}$ across each of METLIN-CCS 25 5x5 cross-validation folds, with the arithmetic mean. C indicates empirical recall, whereas C' denotes the minimum empirical recall attained across the Mondrian taxonomies. $i = 100 - \alpha$.

	**GraphCCS**			**CCSP2.0**			**SigmaCCS**			**KerasECFP**			**LinMZ**		
**Fold**	C'_{99} (C_{99})	C'_{95} (C_{95})	C'_{90} (C_{90})	C'_{99} (C_{99})	C'_{95} (C_{95})	C'_{90} (C_{90})	C'_{99} (C_{99})	C'_{95} (C_{95})	C'_{90} (C_{90})	C'_{99} (C_{99})	C'_{95} (C_{95})	C'_{90} (C_{90})	C'_{99} (C_{99})	C'_{95} (C_{95})	C'_{90} (C_{90})
1	98.9 (99.0)	95.2 (95.8)	89.5 (91.3)	98.9 (99.3)	94.8 (95.3)	88.2 (90.3)	98.7 (98.9)	94.9 (95.3)	89.6 (90.6)	98.9 (99.0)	95.5 (95.6)	89.5 (90.3)	99.2 (99.3)	95.3 (95.8)	89.6 (90.9)
2	98.9 (99.2)	93.9 (94.3)	89.1 (89.3)	98.7 (99.1)	93.9 (94.3)	88.2 (88.9)	99.0 (99.3)	94.1 (94.9)	88.1 (89.3)	98.9 (99.1)	93.7 (94.2)	88.6 (89.6)	98.3 (98.5)	94.0 (94.3)	88.1 (89.6)
3	98.7 (98.9)	94.4 (95.3)	89.8 (90.4)	99.2 (99.3)	95.2 (95.7)	90.7 (91.4)	99.2 (99.3)	94.9 (95.3)	89.9 (90.4)	98.9 (99.1)	95.0 (95.4)	89.7 (90.0)	99.1 (99.2)	94.7 (95.2)	89.5 (90.4)
4	99.1 (99.2)	94.4 (94.8)	90.1 (90.3)	98.5 (99.0)	95.0 (95.4)	89.7 (89.9)	98.9 (99.2)	94.7 (94.8)	89.1 (89.5)	99.0 (99.1)	94.2 (94.9)	87.3 (89.8)	99.1 (99.3)	95.0 (95.5)	89.9 (90.3)
5	99.1 (99.2)	94.6 (95.3)	89.1 (90.2)	98.8 (99.1)	94.5 (95.2)	88.3 (89.1)	99.1 (99.1)	94.4 (95.1)	88.9 (89.8)	98.4 (98.7)	93.8 (94.9)	87.1 (89.0)	98.7 (99.0)	94.0 (94.5)	88.2 (89.5)
6	98.4 (98.9)	94.5 (94.9)	88.5 (89.8)	98.9 (99.1)	94.0 (95.0)	88.9 (89.8)	98.3 (99.0)	94.4 (95.1)	89.4 (89.8)	98.5 (99.1)	94.2 (95.0)	89.3 (90.0)	98.6 (99.1)	94.3 (95.2)	89.3 (89.7)
7	99.1 (99.2)	93.6 (94.1)	88.7 (89.2)	98.8 (98.9)	94.9 (95.3)	89.4 (89.7)	99.2 (99.3)	95.0 (95.1)	89.7 (90.1)	98.9 (99.1)	94.3 (94.8)	89.0 (89.7)	98.6 (98.9)	94.3 (94.8)	89.0 (89.1)
8	99.0 (99.0)	95.3 (95.8)	91.0 (91.5)	99.1 (99.2)	95.2 (95.4)	90.7 (91.2)	98.7 (99.1)	95.6 (95.8)	90.7 (91.1)	98.7 (99.0)	95.5 (95.8)	90.4 (91.3)	98.7 (98.9)	95.1 (95.6)	89.3 (90.5)
9	98.5 (99.1)	94.7 (95.2)	89.0 (90.0)	98.5 (98.9)	94.3 (94.9)	89.5 (90.0)	98.9 (99.2)	95.0 (95.3)	90.4 (90.6)	98.8 (99.0)	94.6 (95.2)	90.0 (90.4)	98.8 (99.0)	94.8 (95.1)	89.6 (90.4)
10	99.0 (99.3)	94.8 (95.1)	90.1 (90.4)	98.9 (99.3)	95.0 (95.7)	89.2 (90.1)	98.8 (99.1)	94.9 (95.3)	88.8 (89.6)	98.9 (99.0)	94.0 (94.5)	89.5 (89.7)	98.6 (99.0)	94.1 (94.6)	89.5 (89.8)
11	98.9 (99.2)	94.7 (95.1)	89.0 (89.8)	98.9 (99.1)	94.7 (95.6)	89.5 (90.2)	99.0 (99.2)	95.2 (95.3)	89.2 (89.8)	99.3 (99.3)	94.8 (95.0)	90.0 (90.1)	98.7 (98.9)	94.6 (95.4)	89.5 (90.1)
12	99.1 (99.2)	93.7 (94.6)	88.3 (89.3)	98.5 (99.0)	94.1 (94.8)	87.8 (89.1)	98.9 (99.0)	93.9 (94.7)	88.1 (89.6)	99.0 (99.1)	94.2 (94.7)	89.4 (90.2)	99.0 (99.1)	94.7 (95.0)	88.7 (89.4)
13	98.7 (99.0)	94.7 (95.2)	90.4 (90.8)	98.6 (99.0)	94.5 (95.0)	89.3 (90.0)	98.3 (99.1)	93.8 (94.6)	89.0 (89.8)	98.9 (98.9)	94.4 (94.7)	87.9 (89.3)	99.0 (99.0)	94.3 (94.7)	89.0 (89.8)
14	98.8 (99.0)	94.1 (94.9)	88.7 (90.3)	98.6 (99.0)	94.4 (95.1)	89.3 (90.4)	99.1 (99.2)	95.1 (95.5)	88.3 (90.1)	98.6 (98.8)	95.0 (95.3)	89.1 (89.7)	98.6 (98.9)	94.5 (95.2)	89.9 (90.2)
15	98.5 (99.0)	94.5 (94.7)	89.5 (89.8)	98.9 (99.2)	94.0 (94.4)	88.9 (89.9)	99.1 (99.1)	94.0 (94.3)	89.4 (90.0)	98.9 (99.1)	94.8 (95.4)	90.1 (90.8)	99.0 (99.3)	94.7 (95.0)	89.3 (90.0)
16	98.4 (98.7)	94.1 (94.8)	89.8 (90.4)	98.4 (99.0)	93.7 (94.6)	89.1 (90.0)	98.5 (98.8)	94.1 (94.6)	88.9 (89.9)	98.5 (99.0)	94.1 (94.6)	89.0 (89.5)	99.0 (99.1)	94.7 (95.1)	88.6 (89.9)
17	98.2 (98.6)	94.2 (94.7)	88.9 (90.0)	98.4 (98.8)	94.0 (94.9)	88.7 (89.9)	98.6 (98.7)	94.5 (94.7)	87.5 (89.5)	98.5 (98.9)	93.7 (94.8)	88.9 (89.8)	98.7 (98.9)	94.4 (94.9)	88.9 (90.2)
18	98.9 (99.1)	94.7 (95.0)	89.5 (90.4)	98.9 (99.2)	93.2 (94.7)	87.3 (89.9)	98.5 (99.1)	95.1 (95.5)	90.1 (90.6)	98.4 (98.9)	94.7 (95.2)	89.3 (89.9)	98.7 (99.0)	94.9 (95.3)	89.7 (90.2)
19	98.9 (99.0)	94.0 (94.9)	89.3 (90.2)	98.8 (99.0)	95.0 (95.2)	89.7 (90.1)	98.9 (99.1)	95.5 (96.1)	90.0 (90.9)	98.9 (99.1)	94.5 (95.1)	89.9 (90.9)	98.9 (99.1)	94.7 (95.0)	89.3 (89.6)
20	99.2 (99.4)	95.0 (95.7)	90.5 (90.9)	99.1 (99.3)	96.0 (96.2)	89.7 (90.3)	99.2 (99.4)	95.3 (95.8)	91.2 (91.2)	99.0 (99.3)	95.3 (95.9)	90.9 (91.4)	99.0 (99.2)	94.5 (95.0)	88.8 (90.0)
21	98.7 (99.3)	94.9 (95.4)	90.7 (91.2)	98.9 (99.3)	94.6 (95.1)	90.5 (91.0)	99.1 (99.2)	94.6 (95.0)	90.2 (90.6)	98.6 (99.2)	94.6 (95.2)	88.9 (90.3)	99.1 (99.3)	95.0 (95.8)	90.7 (91.4)
22	99.3 (99.3)	95.1 (95.3)	89.0 (90.0)	99.3 (99.4)	95.5 (95.8)	90.6 (90.9)	98.8 (99.3)	95.3 (95.8)	90.3 (90.6)	99.1 (99.2)	95.0 (95.8)	90.8 (91.1)	99.0 (99.1)	95.0 (95.5)	90.3 (90.7)
23	98.7 (98.8)	93.7 (94.2)	88.1 (89.0)	98.6 (98.8)	93.7 (94.0)	88.6 (88.8)	98.9 (99.0)	94.5 (94.6)	88.6 (89.3)	98.7 (98.9)	94.4 (94.7)	87.9 (88.7)	98.5 (98.9)	94.0 (94.7)	88.3 (88.9)
24	99.0 (99.2)	94.6 (95.4)	89.6 (90.1)	98.2 (98.9)	94.4 (95.4)	89.4 (90.3)	98.5 (99.1)	95.0 (95.4)	90.5 (91.0)	98.8 (99.2)	95.5 (95.5)	90.0 (90.4)	98.9 (99.1)	94.8 (95.4)	89.3 (90.5)
25	98.9 (99.0)	93.9 (94.1)	87.8 (88.8)	98.8 (98.9)	94.0 (94.6)	88.3 (89.3)	98.7 (98.8)	93.7 (94.3)	88.6 (88.7)	98.9 (99.0)	94.2 (94.5)	89.4 (89.7)	98.4 (98.8)	94.0 (94.3)	89.1 (89.3)
**AVG**	98.8 (99.1)	94.4 (95.0)	89.4 (90.1)	98.8 (99.1)	94.5 (95.1)	89.2 (90.0)	98.8 (99.1)	94.7 (95.1)	89.4 (90.1)	98.8 (99.0)	94.6 (95.1)	89.3 (90.1)	98.8 (99.0)	94.6 (95.1)	89.3 (90.0)

Point Estimation Performance for EnvCCS and AllCCS

Table S7. Point-estimation performance metrics of GraphCCS for EnvCCS and for each ion type therein.

Subset	*n*	R^2_{EnvCCS}	$RMSE_{EnvCCS}$	MAE_{EnvCCS}	$MDAE_{EnvCCS}$	$MAPE_{EnvCCS}$	$^{90}PAE_{EnvCCS}$
**Global**	2888	0.772	17.46	6.58	3.54	3.49	12.23
**$[M+H]^+$**	921	0.875	11.84	5.28	2.90	2.85	9.11
**$[M+Na]^+$**	868	0.886	9.94	6.27	4.19	3.29	13.42
**$[M-H]^-$**	1099	0.649	24.61	7.93	3.72	4.17	12.86

R^2: R^2 score; $RMSE$: root-mean-squared error; MAE: mean absolute error; $MdAE$: median absolute error; $MAPE$: mean absolute percentage error; ^{90}PAE: 90th percentile of percentage absolute errors.

Table S8. Point-estimation performance metrics of GraphCCS for AllCCS and for each ion type therein.

Subset	*n*	R^2_{AllCCS}	$RMSE_{AllCCS}$	MAE_{AllCCS}	$MDAE_{AllCCS}$	$MAPE_{AllCCS}$	$^{90}PAE_{AllCCS}$
**Global**	990	0.939	9.60	6.44	4.27	3.64	14.44
**$[M+H]^+$**	419	0.961	7.94	5.29	3.54	2.86	10.69
**$[M+Na]^+$**	301	0.891	11.50	7.38	4.23	4.16	17.95
**$[M-H]^-$**	270	0.944	9.62	7.17	5.50	4.26	15.30

R^2: R^2 score; $RMSE$: root-mean-squared error; MAE: mean absolute error; $MdAE$: median absolute error; $MAPE$: mean absolute percentage error; ^{90}PAE: 90th percentile of percentage absolute errors.

Recall at the Boundaries of the Applicability Domain

Random fluctuations without calibration-conditional validity can be approximated with a beta-binomial distribution under target recall, $\frac{100}{n_{test}}\mathrm{BetaBinom}(n_{test}, n_{cal}^{CCS} + 1 - \beta, \beta)$, where $\beta = \lfloor (n_{cal}^{CCS} + 1)\alpha/100 \rfloor$.[4] Due to the Mondrian classes, n_{cal}^{CCS} was calculated as the weighted average of the number of calibrants per taxonomy, where the weights are the proportion of test instances per taxonomy.

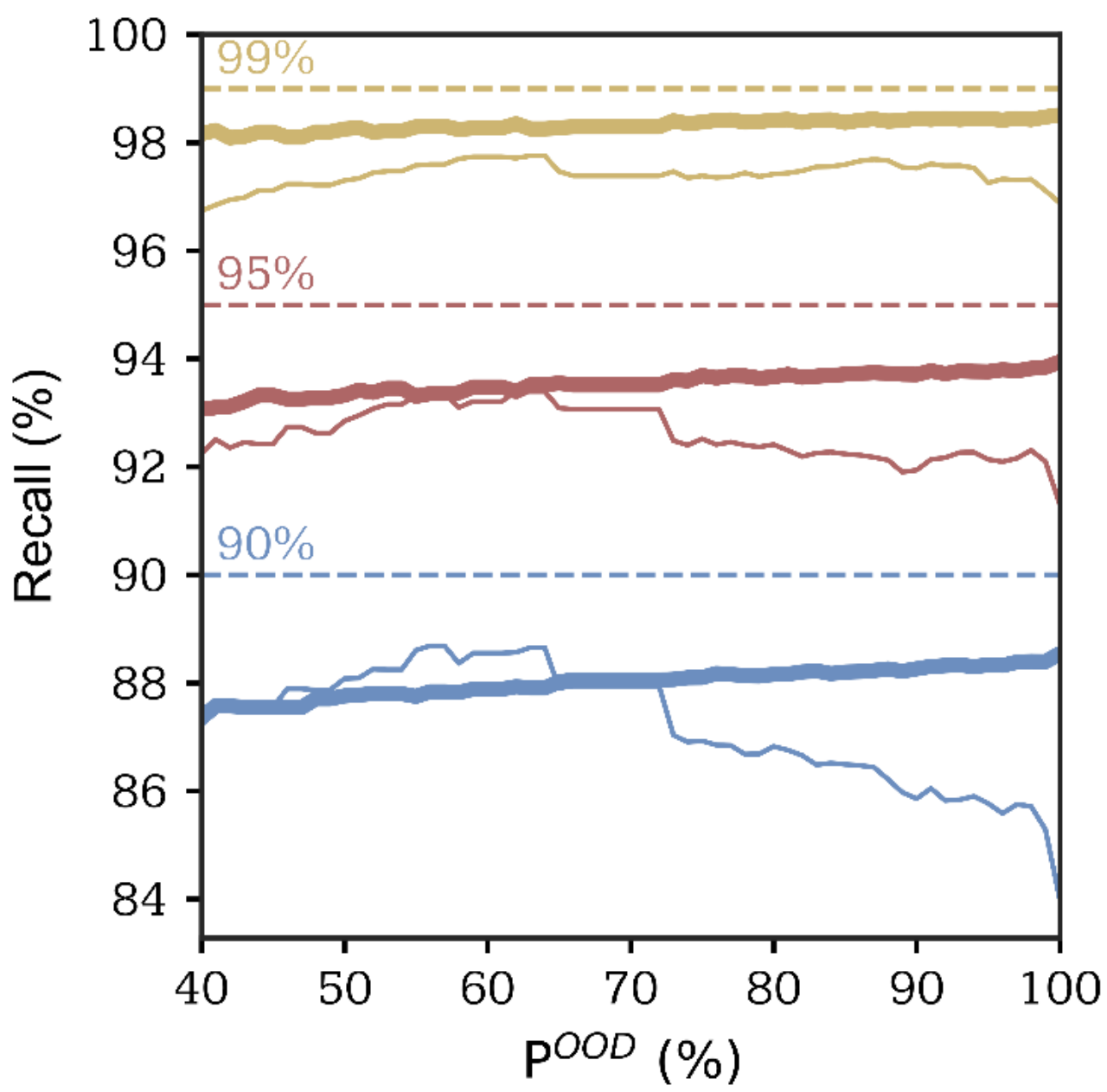

Figure S28. Thin solid lines show empirical recall for EnvCCS dataset at α levels 10% (blue), 5% (red) and 1% (yellow), calculated with ion species that have a P^{OOD} lower than or equal to a given threshold, which ranges from 40% to 100% -the latter corresponding to no filtering-, in steps of 1%. 40% was selected as the minimum threshold due to the limited number of ion species at lower values. Dashed horizontal colored lines indicate the corresponding target recall levels. Thick solid lines represent the 5th beta-binomial lower-tail percentile under target recall.

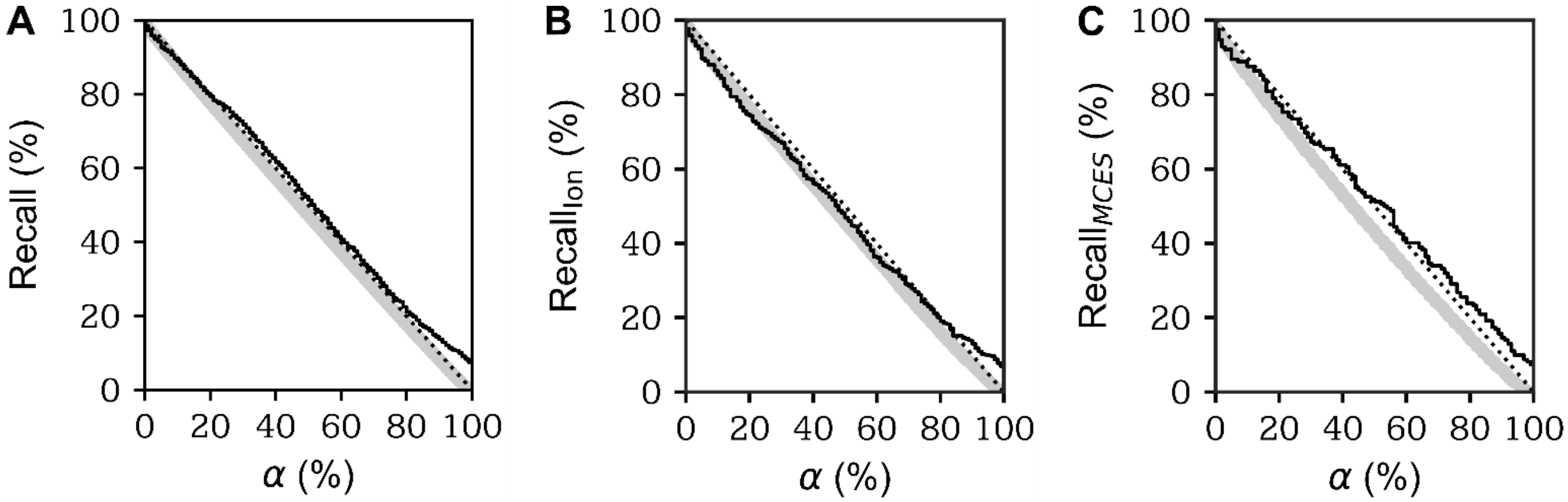

Figure S29. Recall (%) vs. α (%) curves (A) across all EnvCCS ion species with $P^{OOD} \leq 90\%$ ($n_{test}^{ID} = 1{,}414$), (B) for the ion type ($n_{test,[\mathrm{M+H}]^+}^{ID} = 467$, $n_{test,[\mathrm{M+Na}]^+}^{ID} = 378$, $n_{test,[\mathrm{M-H}]^-}^{ID} = 569$) with the lowest empirical recall at each α value and (C) for the region of the $\langle MCES \rangle_{k=46}$ dimension with the lowest empirical recall at each α value. The dotted black line indicates the target recall. The grey thicker line indicates the lower bound given by the 5th binomial lower-tail percentile under target recall. Note that only empirical recall in A and B is guaranteed by the Mondrian construction to lie close to the target recall. Also note that empirical recall does not reach 0% a $\alpha = 100\%$ due to the calibration-conditional validity correction. To obtain the $\langle MCES \rangle_{k=46}$ classes for (C), the equal-size discretization approach explained in the main text in Methods – Evaluation Measures – Recall was applied. This resulted in 9 classes with the following $\langle MCES \rangle_{k=46}$ edges: 5.86 – 9.00 – 9.42 – 9.74 – 9.87 – 9.95 – 10.00 – 10.05 – 10.22 – 10.34.

Efficiency in NTS Prioritization

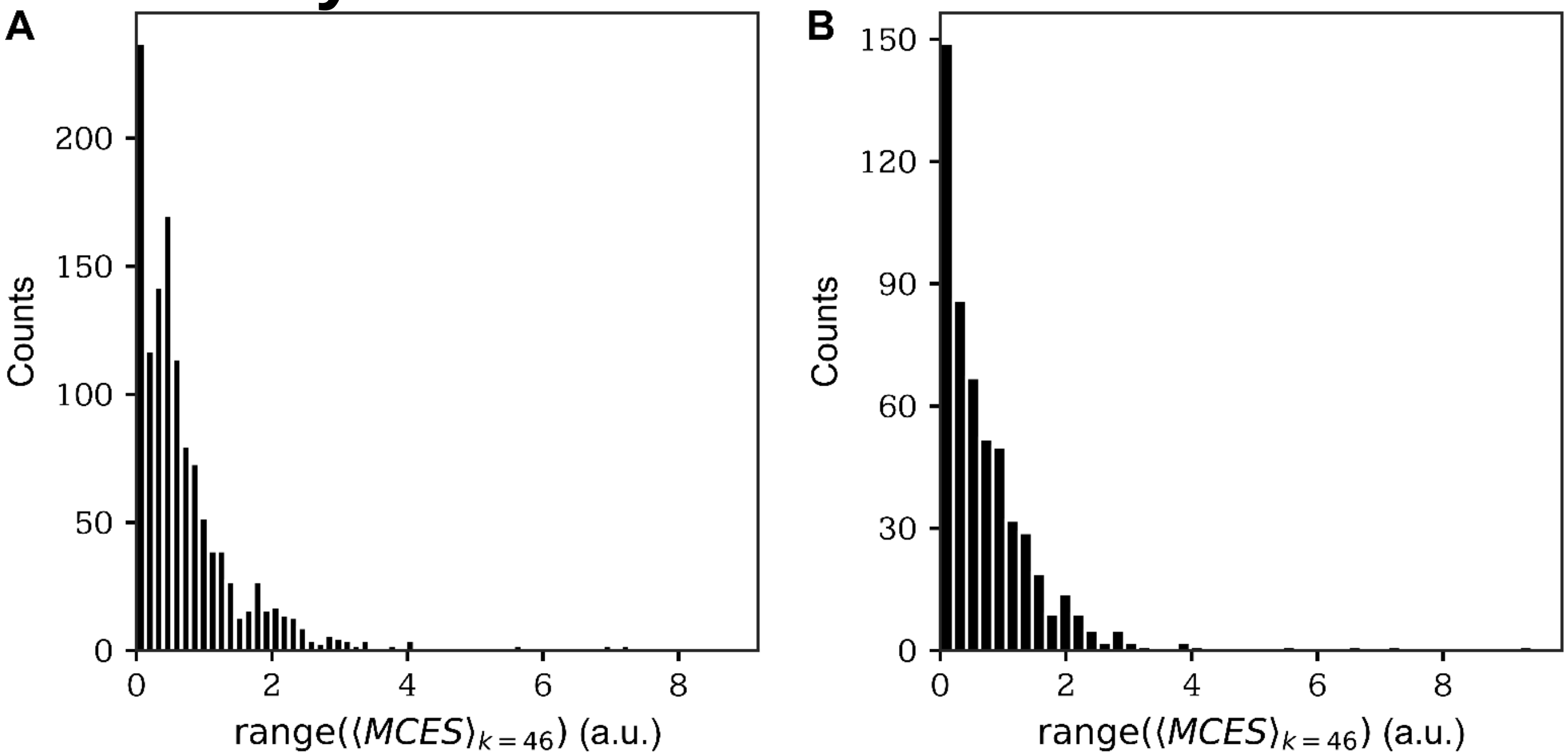

Figure S30. Histogram counts of the range of $\langle MCES \rangle_{k=46}$ for the candidate structures of each NTS feature corresponding to EnvCCS_NTS (A) and AllCCS_NTS (B) datasets.

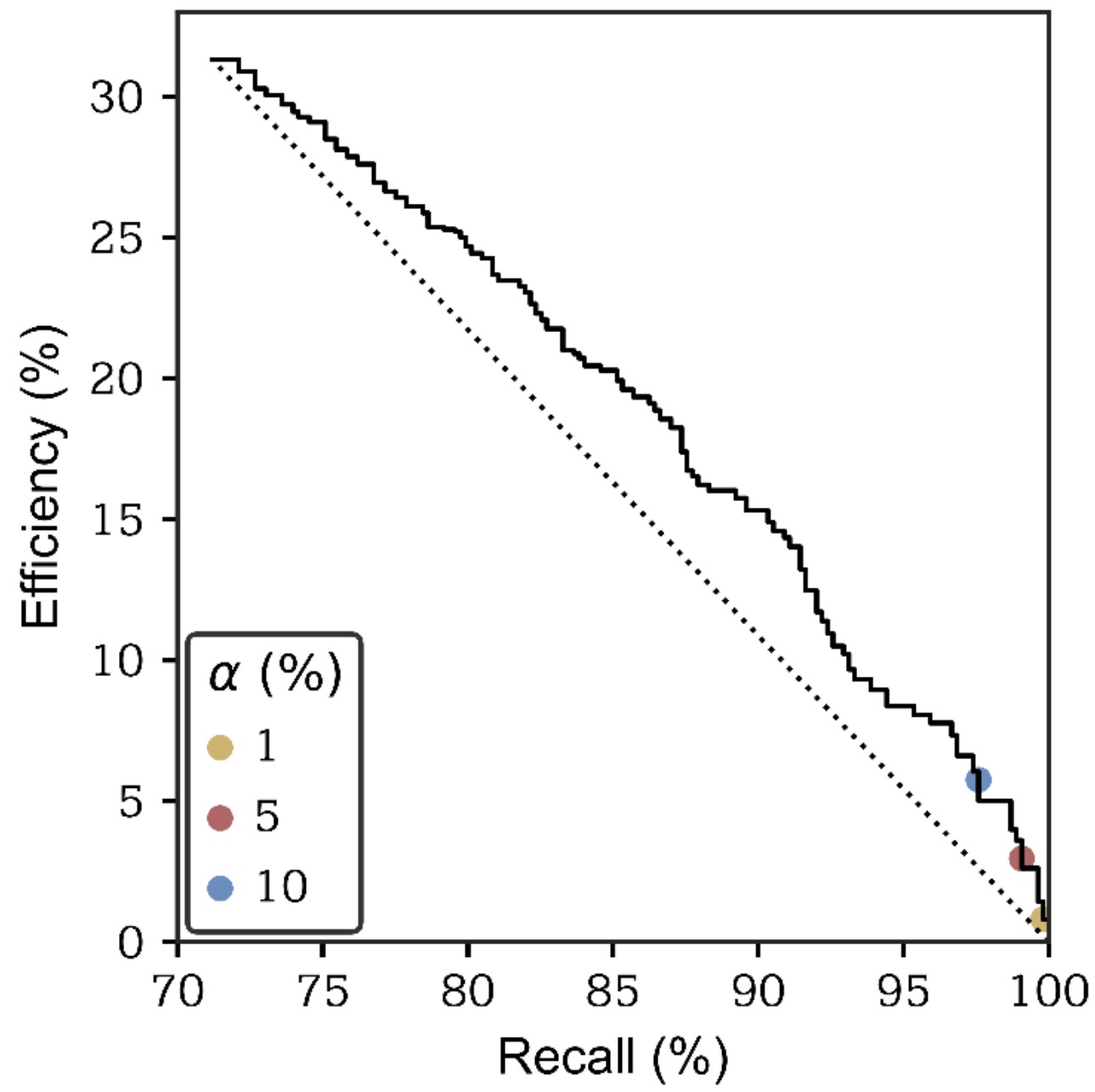

Figure S31. Trade-off between efficiency (percentage of wrong NTS candidate structures filtered out) and recall (percentage of correct NTS candidate structures retained), shown by the black solid line, when prioritizing structures corresponding to AllCCS_NTS dataset as $\mathbb{P}_{ccv}^{CCS} \geq \alpha$ at different $\alpha \in [0\%, 100\%]$, where 0% indicates no removal. If a candidate showed a $P^{OOD} > 90\%$ it was always retained, as it cannot be confidently evaluated with the conformal predictive system. As a result of OOD detection and the correction for calibration-conditional validity, 69% and 71% of the wrong and true candidate structures, respectively, were filtered-in across all α. The dotted black line indicates the theoretical performance of random prioritization. Yellow, red and blue scatter points indicate performances at α values 1%, 5% and 10%, respectively. Note that due to the candidates that were always retained, the random baseline lies above the diagonal and empirical recall appears higher than the target recall given by $100 - \alpha$.

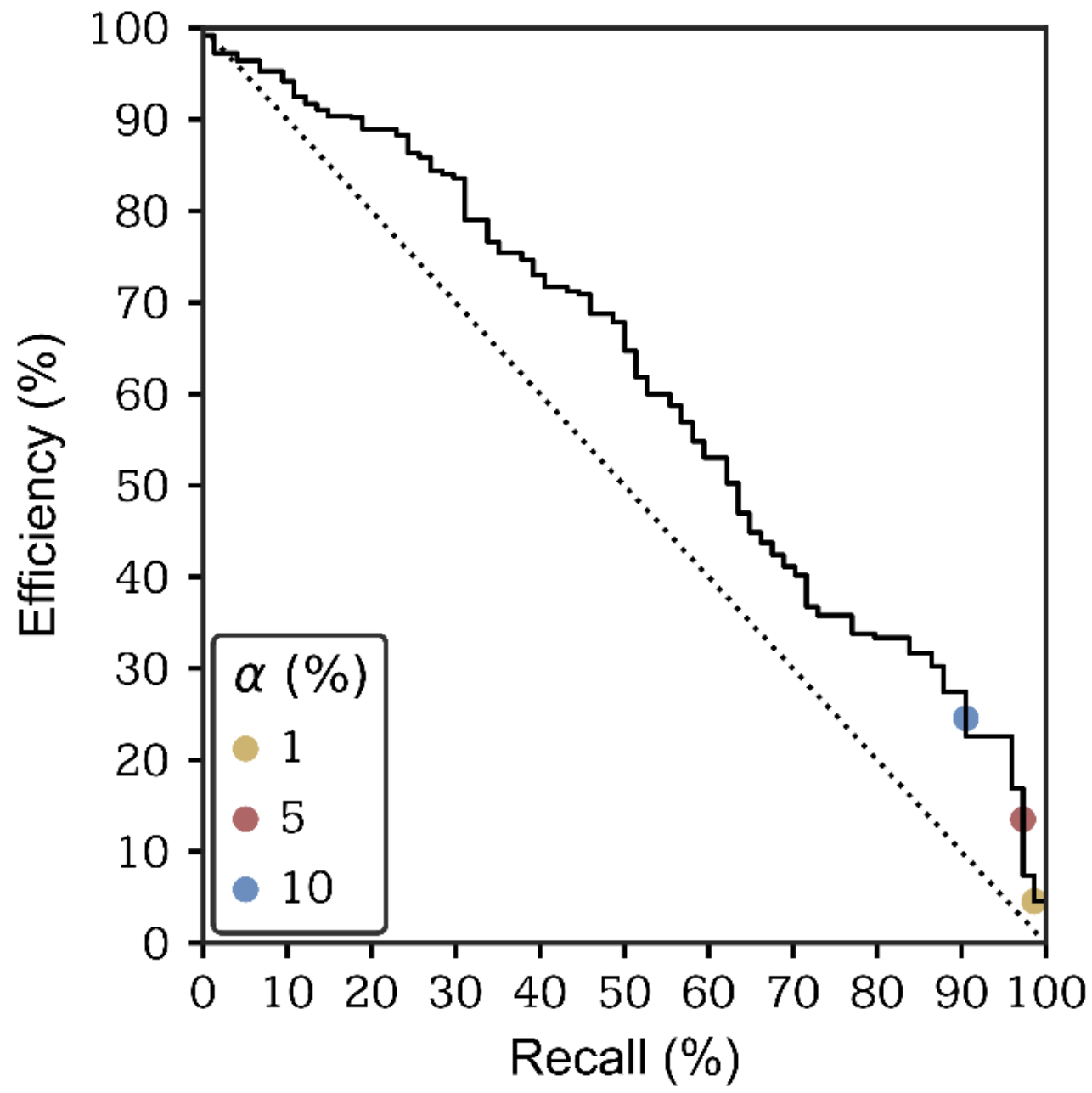

Figure S32. Trade-off between efficiency (percentage of wrong NTS candidate structures filtered out) and recall (percentage of correct NTS candidate structures retained), shown by the black solid line, when prioritizing structures corresponding to the AllCCS_NTS dataset as $\mathbb{P}_{ccv}^{CCS} \geq \alpha$ at different $\alpha \in [0\%, 100\%]$, where 0% indicates no removal. The dotted black line indicates the theoretical performance of random prioritization. Yellow, red and blue scatter points indicate performances at α values 1%, 5% and 10%, respectively. Only NTS features for which the entire candidate list was estimated to be in-domain and for which the calibration-conditional validity correction yielded a $\mathbb{P}_{ccv}^{CCS}$ <100% were considered. This filtering step retained 74 NTS features, comprising ~690 candidate structures.

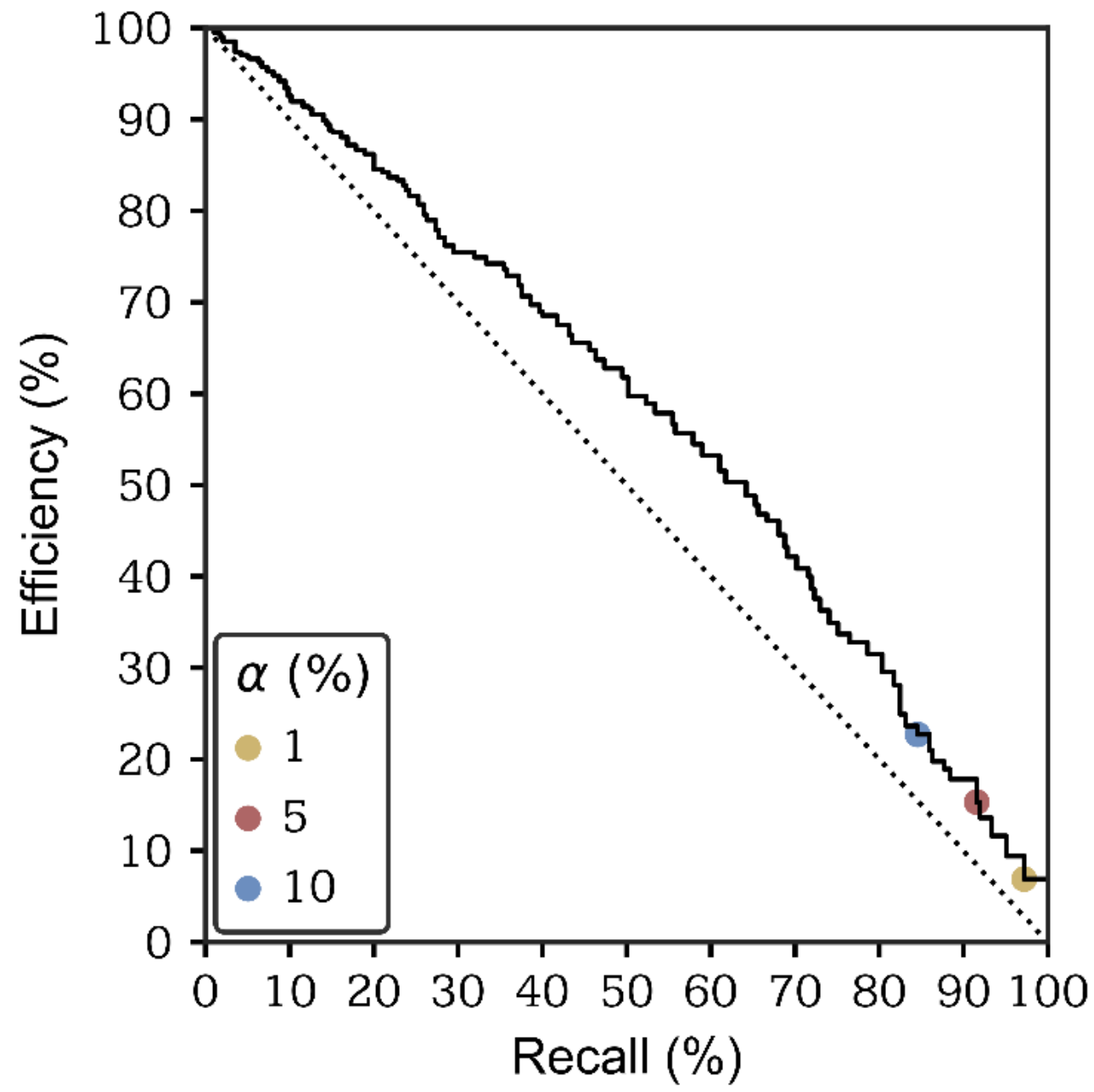

Figure S33. Trade-off between efficiency (percentage of wrong NTS candidate structures filtered out) and recall (percentage of correct NTS candidate structures retained), shown by the black solid line, when prioritizing structures corresponding to the EnvCCS_NTS dataset as $\mathbb{P}_{ccv}^{CCS} \geq \alpha$ at different $\alpha \in [0\%, 100\%]$, where 0% indicates no removal. The dotted black line indicates the theoretical performance of random prioritization. Yellow, red and blue scatter points indicate performances at α values 1%, 5% and 10%, respectively. Only NTS features for which the entire candidate list was estimated to be in-domain and for which the calibration-conditional validity correction yielded a $\mathbb{P}_{ccv}^{CCS}$ <100% were considered. This filtering step retained 285 NTS features, comprising ~2,800 candidate structures.

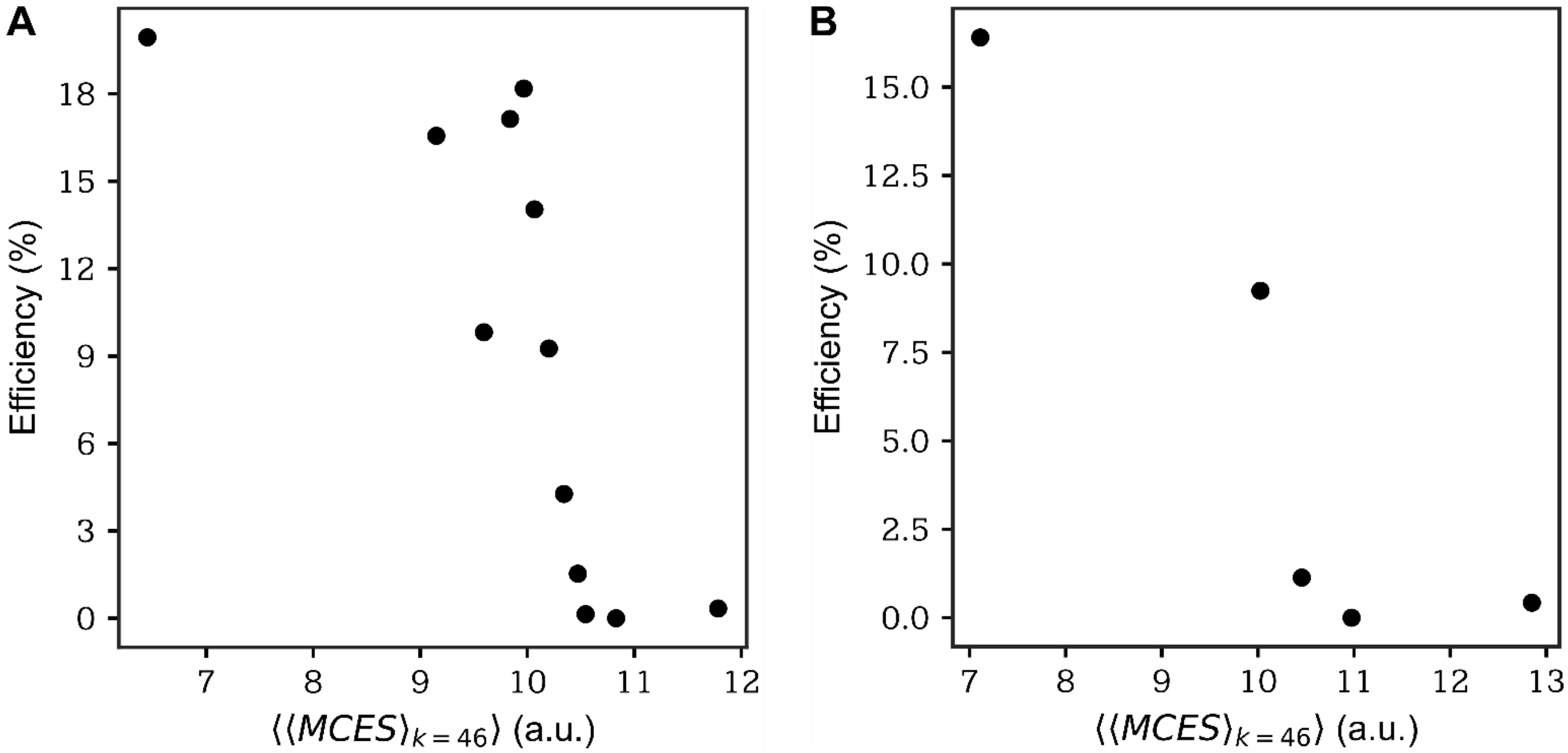

Figure S34. Efficiency (%) at $\alpha = 10\%$ in different regions of the mean distance of the candidate structures per NTS feature to the calibrated chemical space, denoted as $\langle\langle MCES\rangle_{k=46}\rangle$, for EnvCCS_NTS (A) and AllCCS_NTS (B) datasets. The *x*-axis labels correspond to the left extremes of the intervals of $\langle\langle MCES\rangle_{k=46}\rangle$, obtained with the quantile discretization explained for $\langle MCES\rangle_{k=46}$ in Methods – Evaluation Measures – Recall, in this case into bins of 100 NTS features (~950 candidates per class).

NTS Prioritization with Raw MS Data

Candidate structures were suggested for 1,584 and 655 MS spectra of ion species of EnvCCS and AllCCS, respectively, of which 1,531 (97%) and 638 (97%) contained the correct structure among the top 10 candidates. The true structure was annotated as the top 1 match in 90% of the ion species and the molecular formula was correct for 90% of the candidate structures, in both datasets. $[M+H]^+$, $[M+Na]^+$ and $[M-H]^-$ counts for raw EnvCCS_NTS are 708, 202 and 621, respectively, and 358, 91 and 189 for raw AllCCS_NTS.

CRPS-based ranking was assessed for 527 NTS features in the raw EnvCCS_NTS dataset and 123 in the raw AllCCS_NTS. For the raw EnvCCS_NTS, 15.2% of true structures were correctly ranked first using CRPS, compared with 13.9% using CCS differences (10.8% random). The ground truth was ranked among the top 5 in 67.6% and 67.2% of the cases, respectively (51.5% random). For the raw AllCCS_NTS, results were similar, with 17.1% vs. 13.0% (12.3% random) for top 1 ranking performance, and 67.5% vs. 65.9% (52.8% random) for top 5.

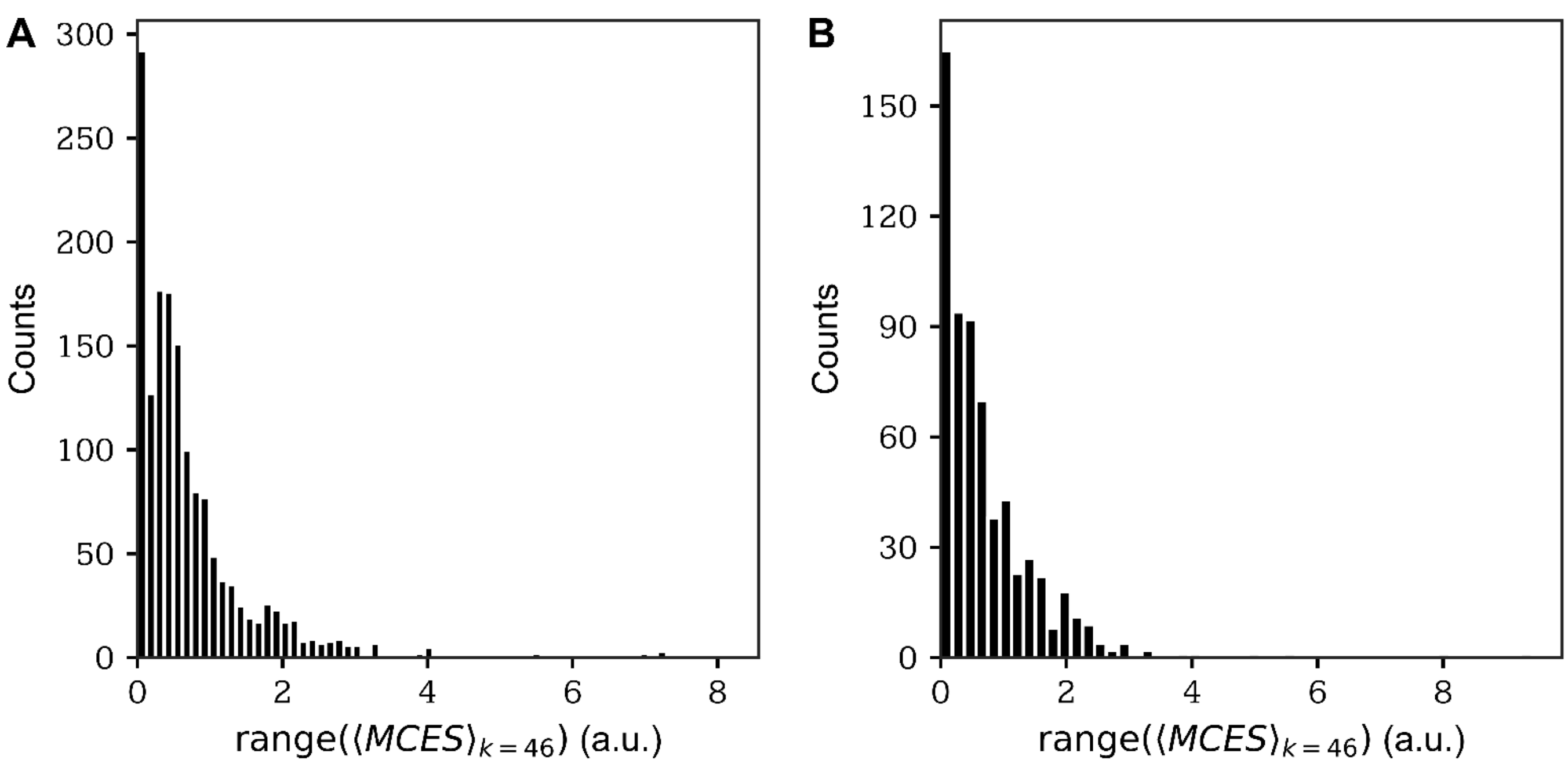

Figure S35. Histogram counts of the range of $\langle MCES\rangle_{k=46}$ for the candidate structures of each NTS feature corresponding to raw EnvCCS_NTS (A) and raw AllCCS_NTS (B) datasets.

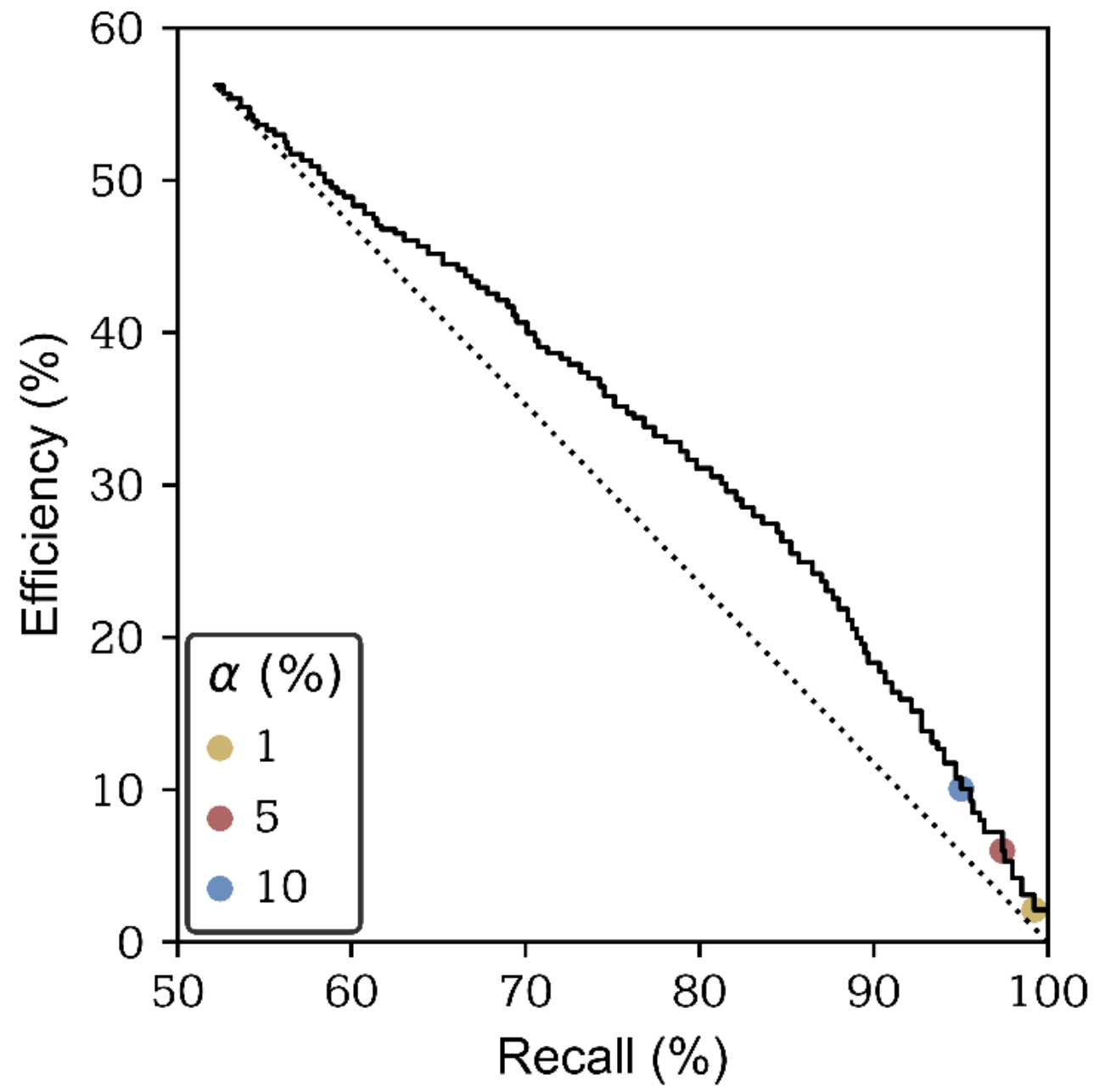

Figure S36. Trade-off between efficiency (percentage of wrong NTS candidate structures filtered out) and recall (percentage of correct NTS candidate structures retained), shown by the black solid line, when prioritizing structures corresponding to the raw EnvCCS_NTS dataset as $\mathbb{P}_{ccv}^{CCS} \geq \alpha$ at different $\alpha \in [0\%, 100\%]$, where 0% indicates no removal. If a candidate showed a P^{OOD} > 90% it was always retained, as it cannot be confidently evaluated with the conformal predictive system. As a result of out-of-domain detection and the correction for calibration-conditional validity, 43% and 52% of the wrong and true candidate structures, respectively, were filtered-in across all α. The dotted black line indicates the theoretical performance of random prioritization. Yellow, red and blue scatter points indicate performances at α values 1%, 5% and 10%, respectively. Note that due to the candidates that were always retained, the random baseline lies above the diagonal and empirical recall appears higher than the target recall given by $100 - \alpha$.

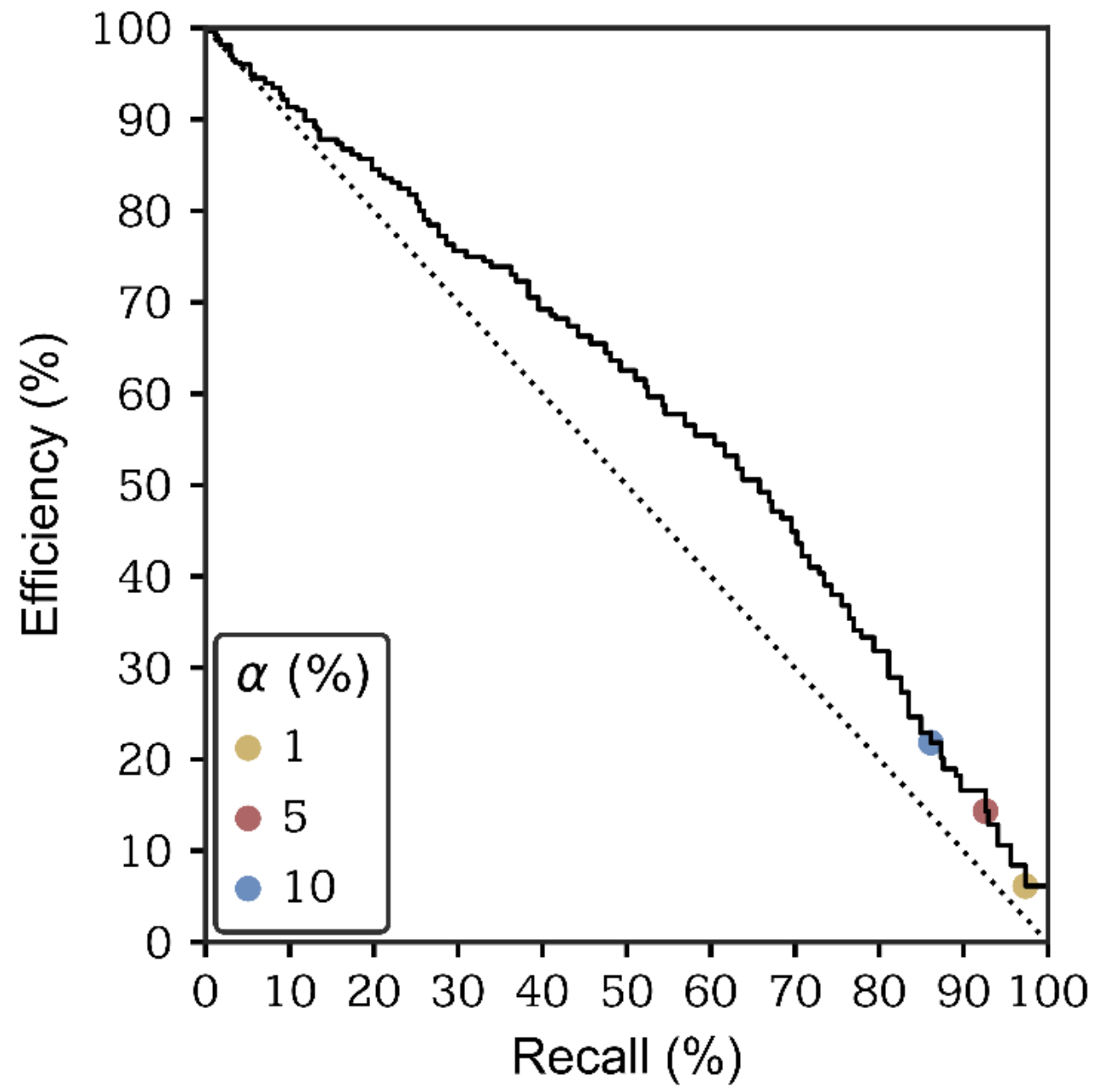

Figure S37. Trade-off between efficiency (percentage of wrong NTS candidate structures filtered out) and recall (percentage of correct NTS candidate structures retained), shown by the black solid line, when prioritizing structures corresponding to the raw EnvCCS_NTS dataset as $\mathbb{P}_{ccv}^{CCS} \geq \alpha$ at different $\alpha \in [0\%, 100\%]$, where 0% indicates no removal. The dotted black line indicates the theoretical performance of random prioritization. Yellow, red and blue scatter points indicate performances at α values 1%, 5% and 10%, respectively. Only NTS features for which the entire candidate list was estimated to be in-domain and for which the calibration-conditional validity correction yielded a $\mathbb{P}_{ccv}^{CCS}$ <100% were considered. This filtering step retained 339 NTS features, comprising ~3,300 candidate structures.

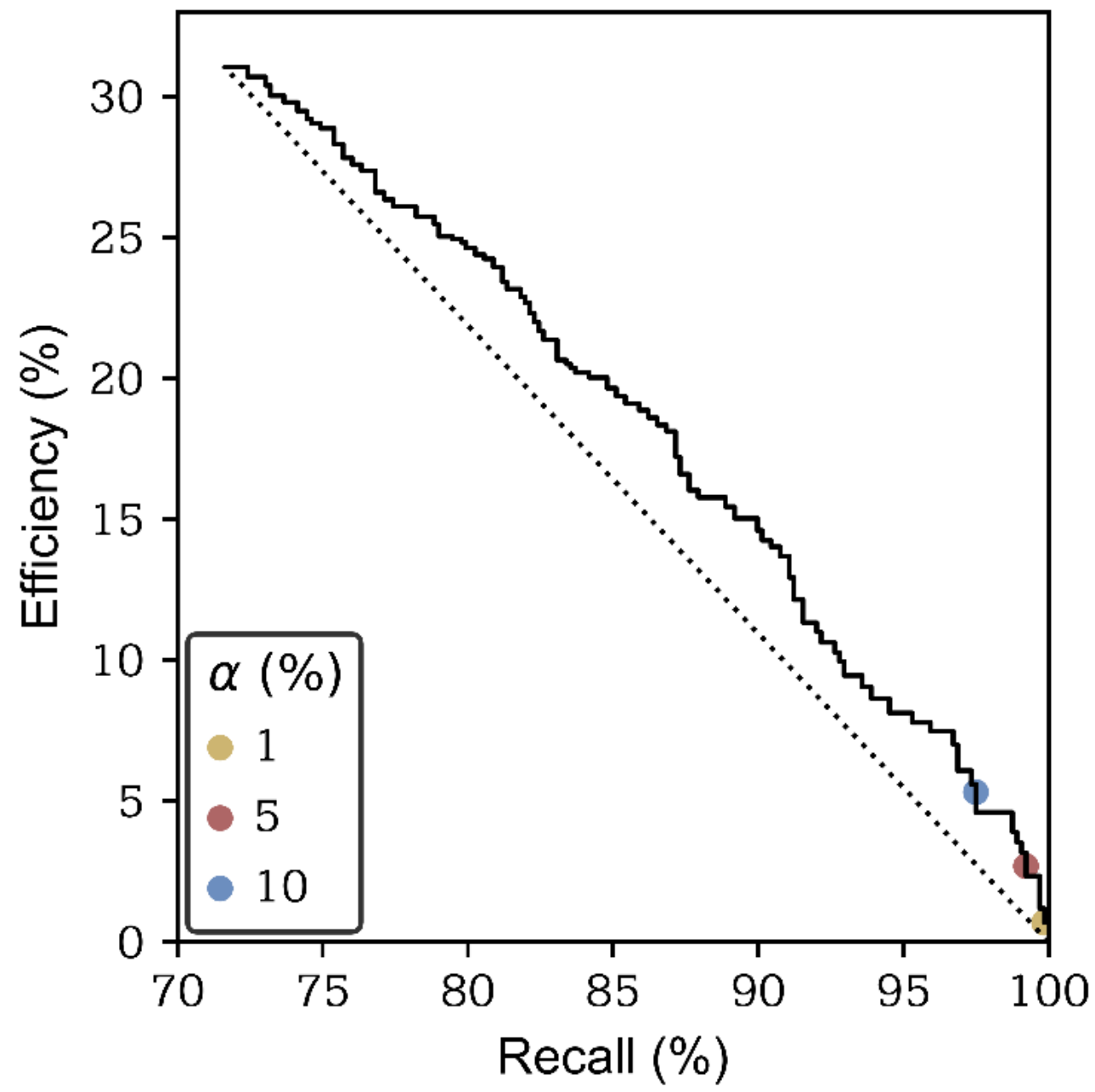

Figure S38. Trade-off between efficiency (percentage of wrong NTS candidate structures filtered out) and recall (percentage of correct NTS candidate structures retained), shown by the black solid line, when prioritizing structures corresponding to the raw AllCCS_NTS dataset as $\mathbb{P}_{ccv}^{CCS} \geq \alpha$ at different $\alpha \in [0\%, 100\%]$, where 0% indicates no removal. If a candidate showed a P^{OOD} > 90% it was always retained, as it cannot be confidently evaluated with the conformal predictive system. As a result of out-of-domain detection and the correction for calibration-conditional validity, 69% and 72% of the wrong and true candidate structures, respectively, were filtered-in across all α. The dotted black line indicates the theoretical performance of random prioritization. Yellow, red and blue scatter points indicate performances at α values 1%, 5% and 10%, respectively. Note that due to the candidates that were always retained, the random baseline lies above the diagonal and empirical recall appears higher than the target recall given by $100 - \alpha$.

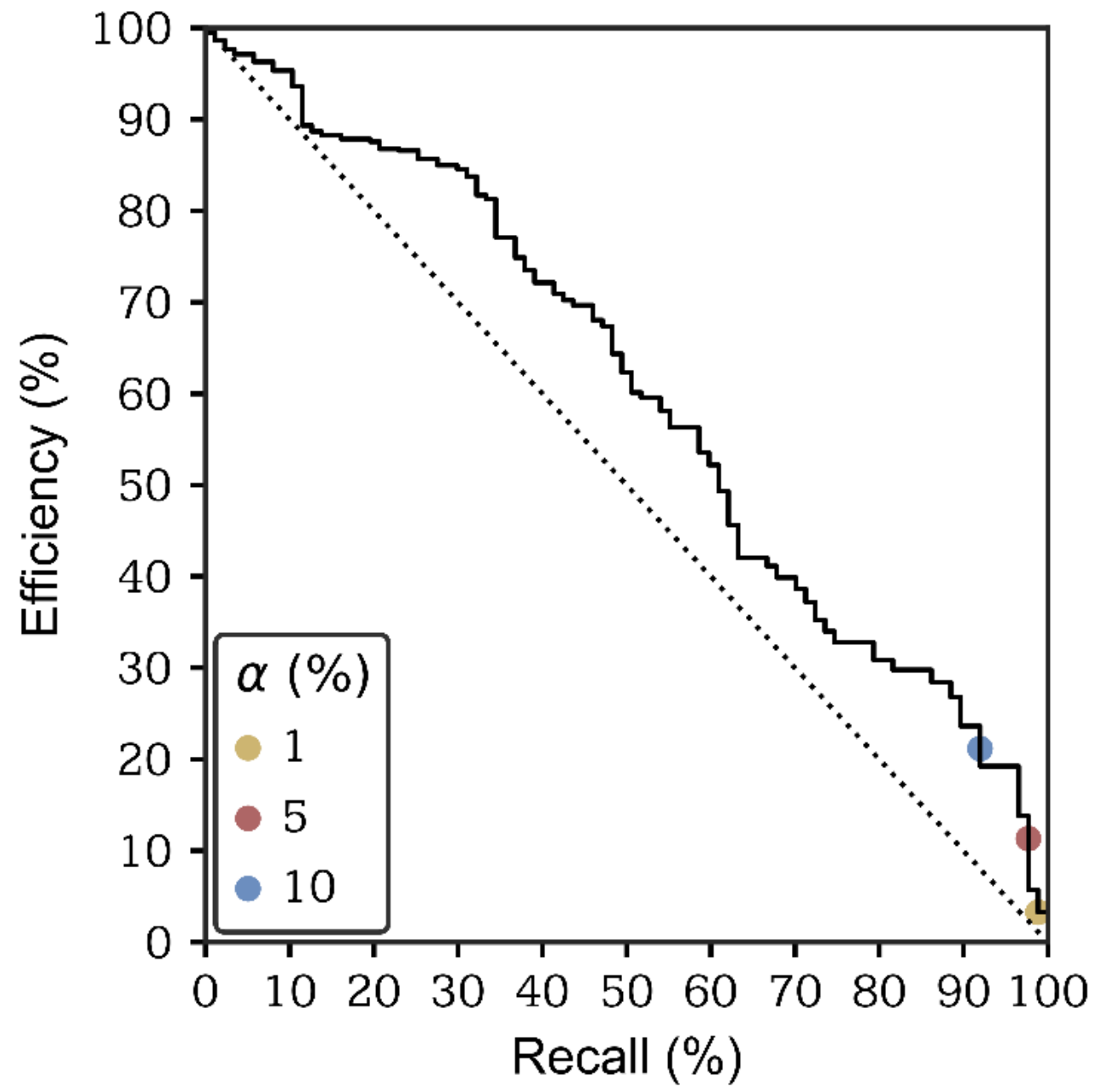

Figure S39. Trade-off between efficiency (percentage of wrong NTS candidate structures filtered out) and recall (percentage of correct NTS candidate structures retained), shown by the black solid line, when prioritizing structures corresponding to the raw AllCCS_NTS dataset as $\mathbb{P}_{ccv}^{CCS} \geq \alpha$ at different $\alpha \in [0\%, 100\%]$, where 0% indicates no removal. The dotted black line indicates the theoretical performance of random prioritization. Yellow, red and blue scatter points indicate performances at α values 1%, 5% and 10%, respectively. Only NTS features for which the entire candidate list was estimated to be in-domain and for which the calibration-conditional validity correction yielded a $\mathbb{P}_{ccv}^{CCS}$ <100% were considered. This filtering step retained 87 NTS features, comprising ~800 candidate structures.

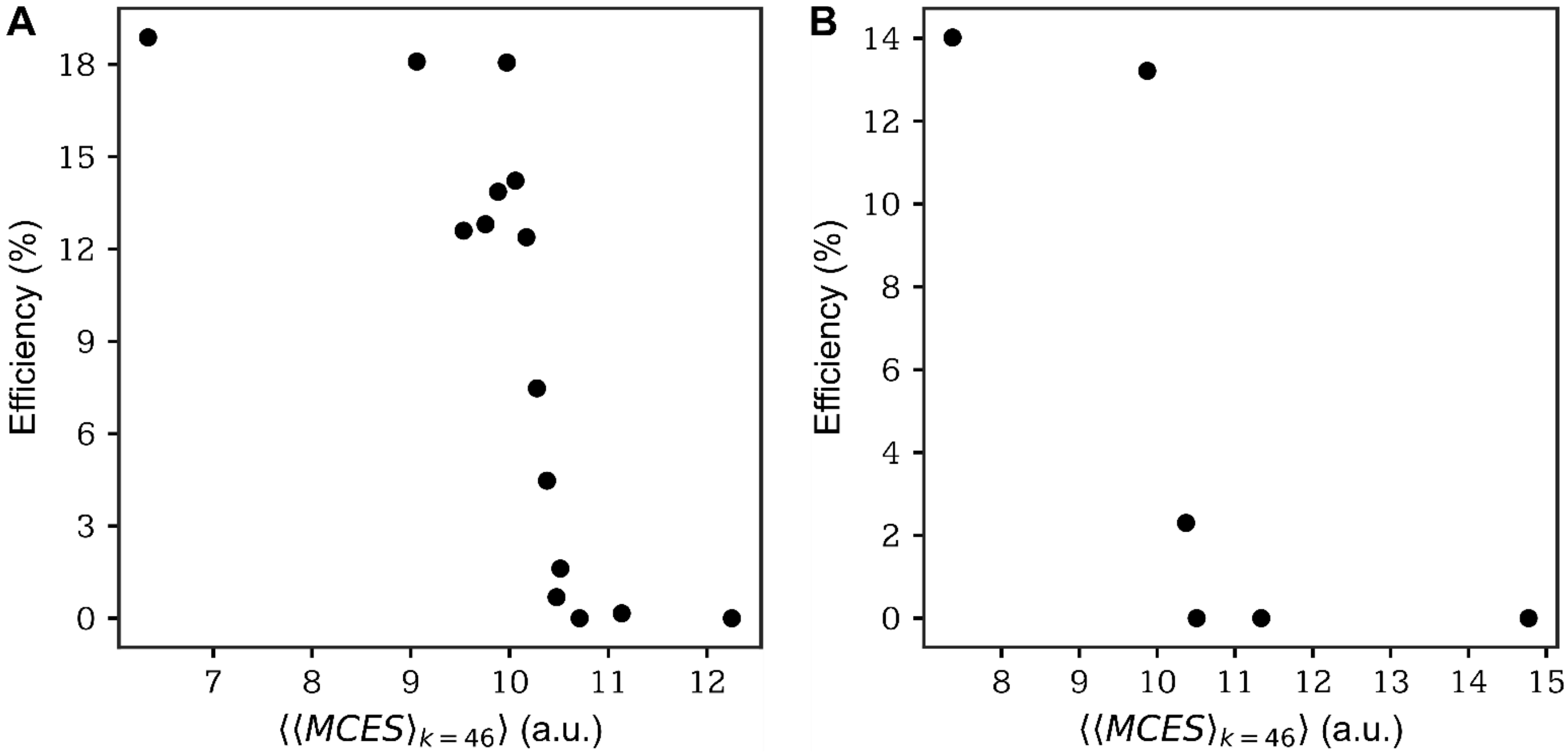

Figure S40. Efficiency (%) at $\alpha = 10\%$ in different regions of the mean distance of the candidate structures per NTS feature to the calibrated chemical space, denoted as $\langle\langle MCES\rangle_{k=46}\rangle$, for the raw EnvCCS_NTS (A) and the raw AllCCS_NTS (B) datasets. The *x*-axis labels correspond to the left extremes of the intervals of $\langle\langle MCES\rangle_{k=46}\rangle$, obtained with the quantile discretization explained for $\langle MCES\rangle_{k=46}$ in Methods – Evaluation Measures – Recall, in this case into bins of 100 NTS features (~930 candidates per class).

We analyze the Mondrian efficiency at $\alpha = 10\%$. Depending on $[M+H]^+$, $[M+Na]^+$ or $[M-H]^-$, the conformal predictive system yielded 14.2%, 7.9% and 4.4% conditional efficiencies, respectively, for raw EnvCCS_NTS as well as 7.4%, 2.3% and 1.8% in the case of raw AllCCS_NTS.

EnvCCS Calibration

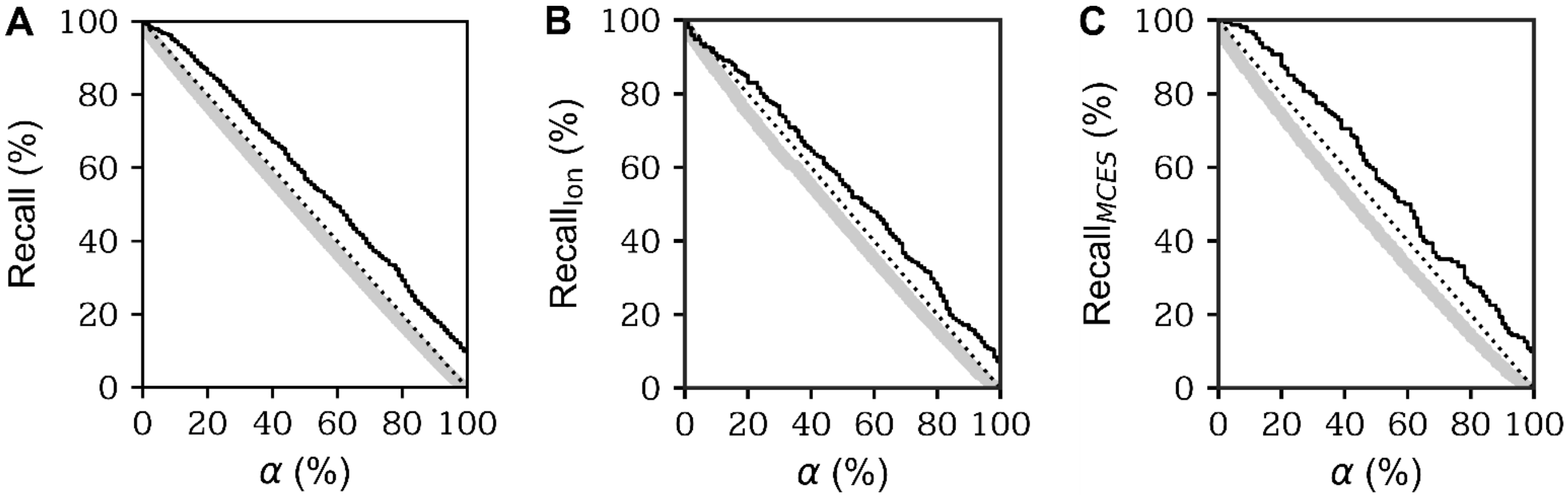

Figure S41. Recall (%) vs. α (%) curves under EnvCCS calibration (A) across all AllCCS ion species with $P^{OOD} \leq 90\%$ ($n_{test}^{ID} = 796$), (B) for the ion type ($n_{test,[M+H]^+}^{ID} = 343$, $n_{test,[M+Na]^+}^{ID} = 247$, $n_{test,[M-H]^-}^{ID} = 206$) with the lowest empirical recall at each α value and (C) for the region of the $\langle MCES \rangle_{k=46}$ dimension with the lowest empirical recall at each α value. The dotted black line indicates the target recall. The grey thicker line indicates the lower bound given by the 5th binomial lower-tail percentile under target recall. Note that only empirical recall in A and B is guaranteed by the Mondrian construction to lie close to the target recall. Also note that empirical recall does not reach 0% a $\alpha = 100\%$ due to the calibration-conditional validity correction. To obtain the $\langle MCES \rangle_{k=46}$ classes for (C), the equal-size discretization approach explained in the main text in Methods – Evaluation Measures – Recall was applied. This resulted in two classes with the following $\langle MCES \rangle_{k=46}$ edges: 7.76 – 9.87 – 10.34.

CRPS-based ranking was assessed for 359 NTS features in AllCCS_NTS dataset and 428 in the raw AllCCS_NTS. For AllCCS_NTS, 16.2% of true structures were correctly ranked first using CRPS, compared with 15.6% using CCS differences (13.2% random). The ground truth was ranked among the top 5 in 63.2% and 62.7% of the cases, respectively (54.1% random). For the raw AllCCS_NTS, results were similar, with 15.0% vs. 14.0% (12.9% random) for top 1 ranking performance, and 62.9% vs. 61.9% (54.0% random) for top 5.

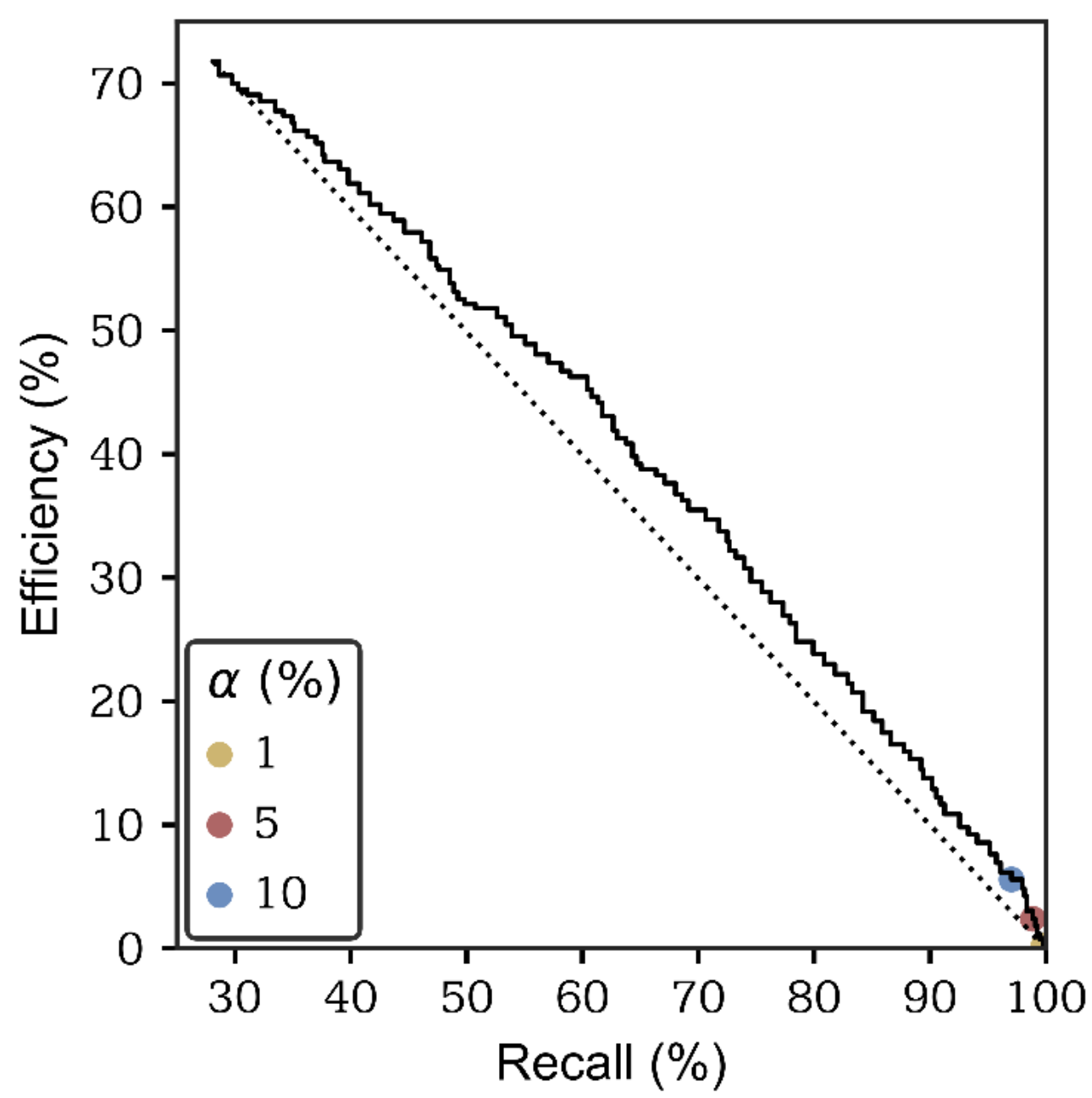

Figure S42. Trade-off under EnvCCS calibration between efficiency (percentage of wrong NTS candidate structures filtered out) and recall (percentage of correct NTS candidate structures retained), shown by the black solid line, when prioritizing structures corresponding to AllCCS_NTS dataset as $\mathbb{P}_{ccv}^{CCS} \geq \alpha$ at different $\alpha \in [0\%, 100\%]$, where 0% indicates no removal. If a candidate showed a $P^{OOD} > 90\%$ it was always retained, as it cannot be confidently evaluated with the conformal predictive system. As a result of out-of-domain detection and the correction for calibration-conditional validity, 29% and 28% of the wrong and true candidate structures, respectively, were filtered-in across all α. The dotted black line indicates the theoretical performance of random prioritization. Yellow, red and blue scatter points indicate performances at α values 1%, 5% and 10%, respectively. Note that due to the candidates that were always retained, the random baseline lies above the diagonal and empirical recall appears higher than the target recall given by $100 - \alpha$.

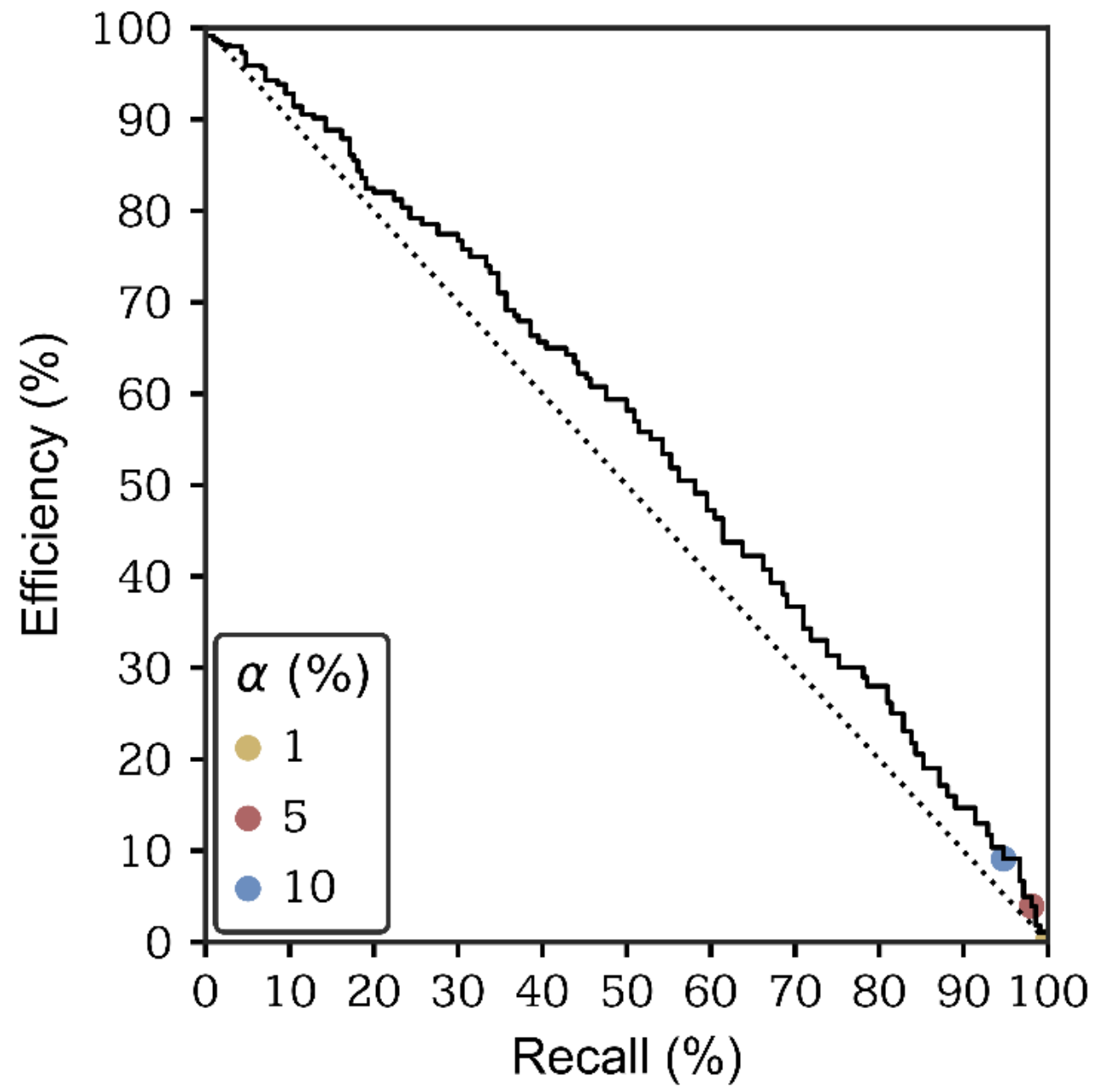

Figure S43. Trade-off under EnvCCS calibration between efficiency (percentage of wrong NTS candidate structures filtered out) and recall (percentage of correct NTS candidate structures retained), shown by the black solid line, when prioritizing structures corresponding to AllCCS_NTS dataset as $\mathbb{P}_{ccv}^{CCS} \geq \alpha$ at different $\alpha \in [0\%, 100\%]$, where 0% indicates no removal. If a candidate showed a P^{OOD} > 90% it was always retained, as it cannot be confidently evaluated with the conformal predictive system. Only NTS features for which the entire candidate list was estimated to be in-domain and for which the calibration-conditional validity correction yielded a $\mathbb{P}_{ccv}^{CCS}$ <100% were considered. This filtering step retained 210 NTS features, comprising ~2,000 candidate structures.

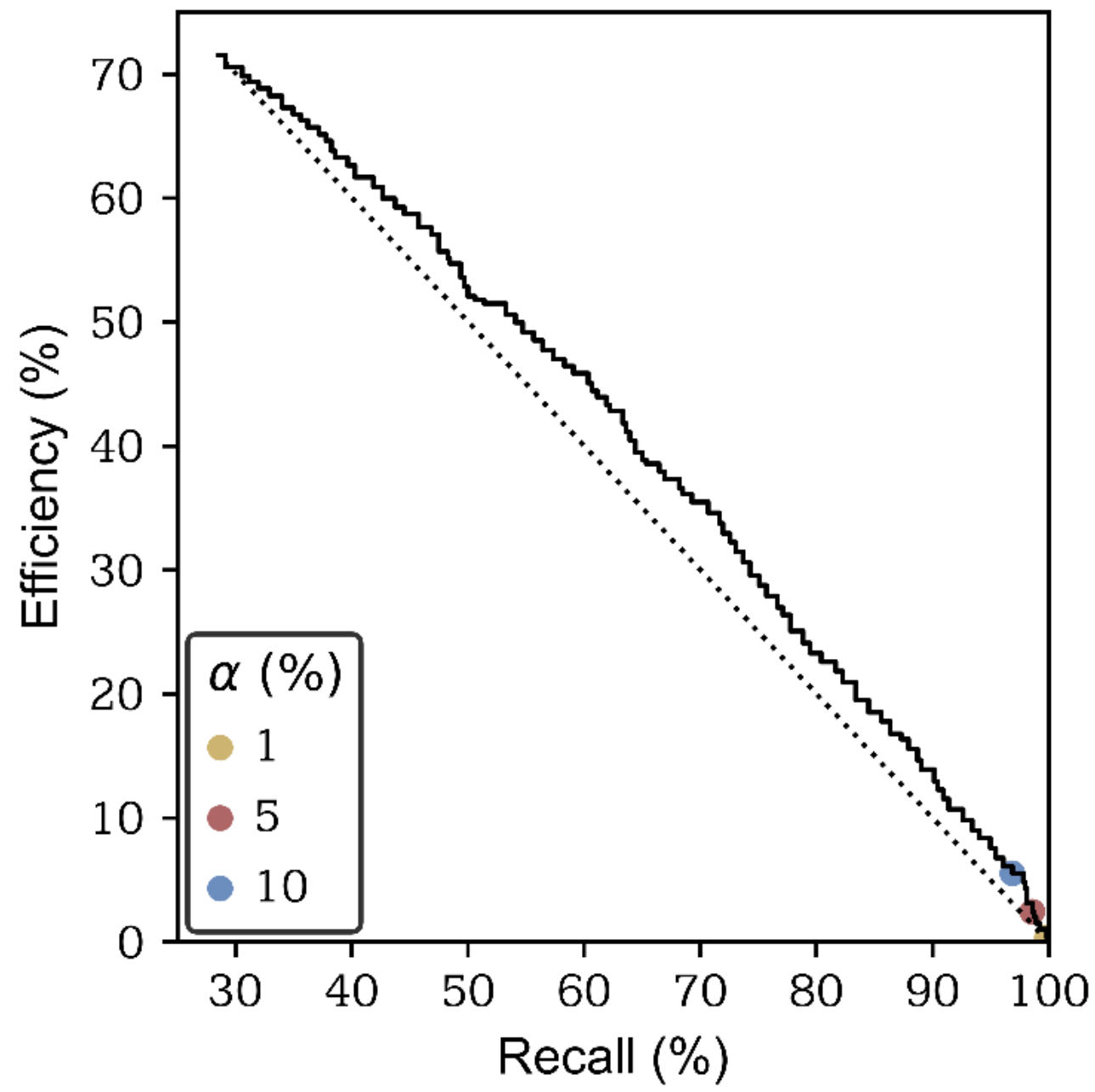

Figure S44. Trade-off under EnvCCS calibration between efficiency (percentage of wrong NTS candidate structures filtered out) and recall (percentage of correct NTS candidate structures retained), shown by the black solid line, when prioritizing structures corresponding to the raw AllCCS_NTS dataset as $\mathbb{P}_{ccv}^{CCS} \geq \alpha$ at different $\alpha \in [0\%, 100\%]$, where 0% indicates no removal. If a candidate showed a P^{OOD} > 90% it was always retained, as it cannot be confidently evaluated with the conformal predictive system. As a result of out-of-domain detection and the correction for calibration-conditional validity, 28% and 29% of the wrong and true candidate structures, respectively, were filtered-in across all α. The dotted black line indicates the theoretical performance of random prioritization. Yellow, red and blue scatter points indicate performances at α values 1%, 5% and 10%, respectively. Note that due to the candidates that were always retained, the random baseline lies above the diagonal and empirical recall appears higher than the target recall given by $100 - \alpha$.

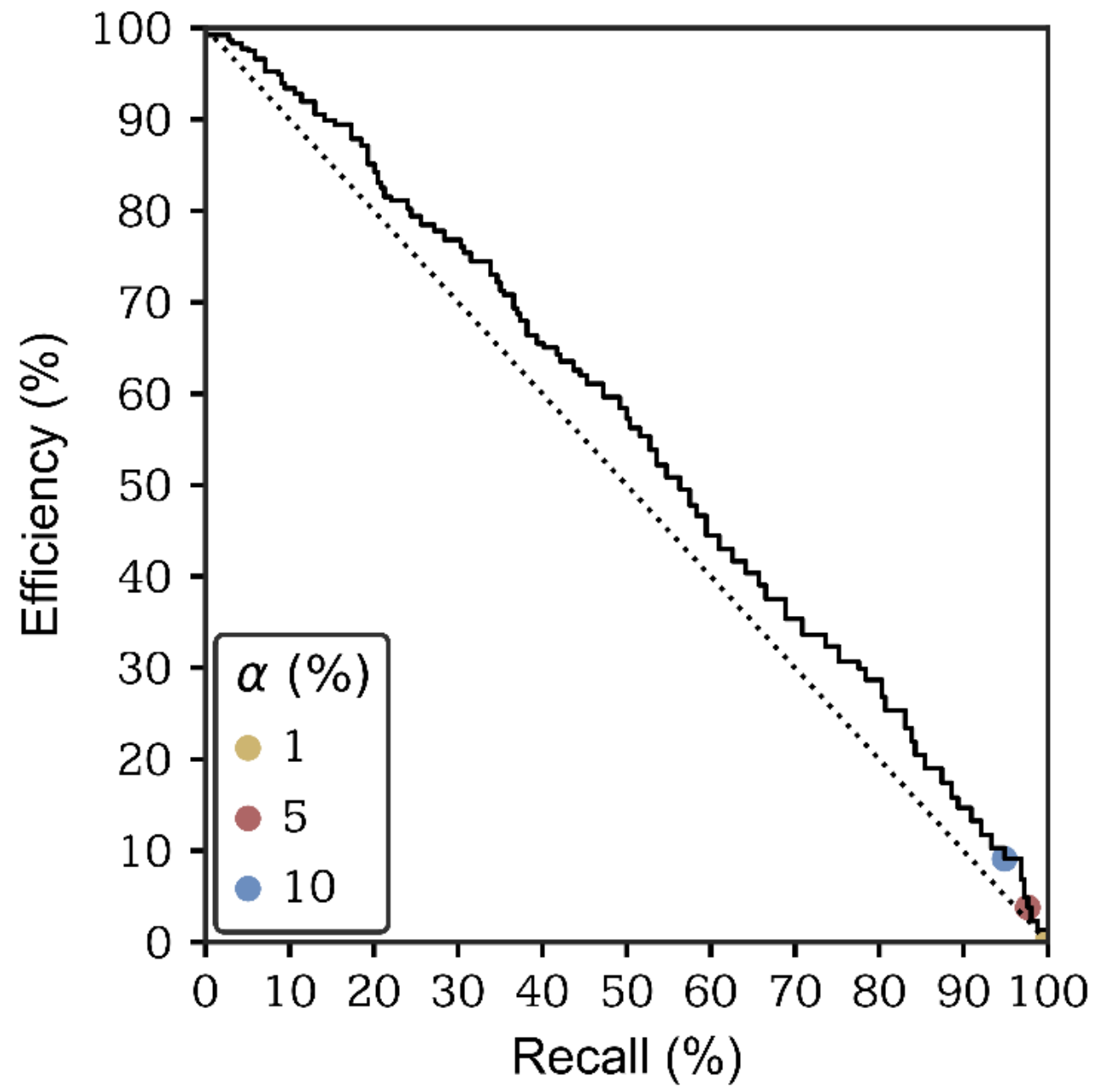

Figure S45. Trade-off under EnvCCS calibration between efficiency (percentage of wrong NTS candidate structures filtered out) and recall (percentage of correct NTS candidate structures retained), shown by the black solid line, when prioritizing structures corresponding to the raw AllCCS_NTS dataset as $\mathbb{P}_{ccv}^{CCS} \geq \alpha$ at different $\alpha \in [0\%, 100\%]$, where 0% indicates no removal. If a candidate showed a P^{OOD} > 90% it was always retained, as it cannot be confidently evaluated with the conformal predictive system. Only NTS features for which the entire candidate list was estimated to be in-domain and for which the calibration-conditional validity correction yielded a $\mathbb{P}_{ccv}^{CCS}$ <100% were considered. This filtering step retained 254 NTS features, comprising ~2,300 candidate structures.

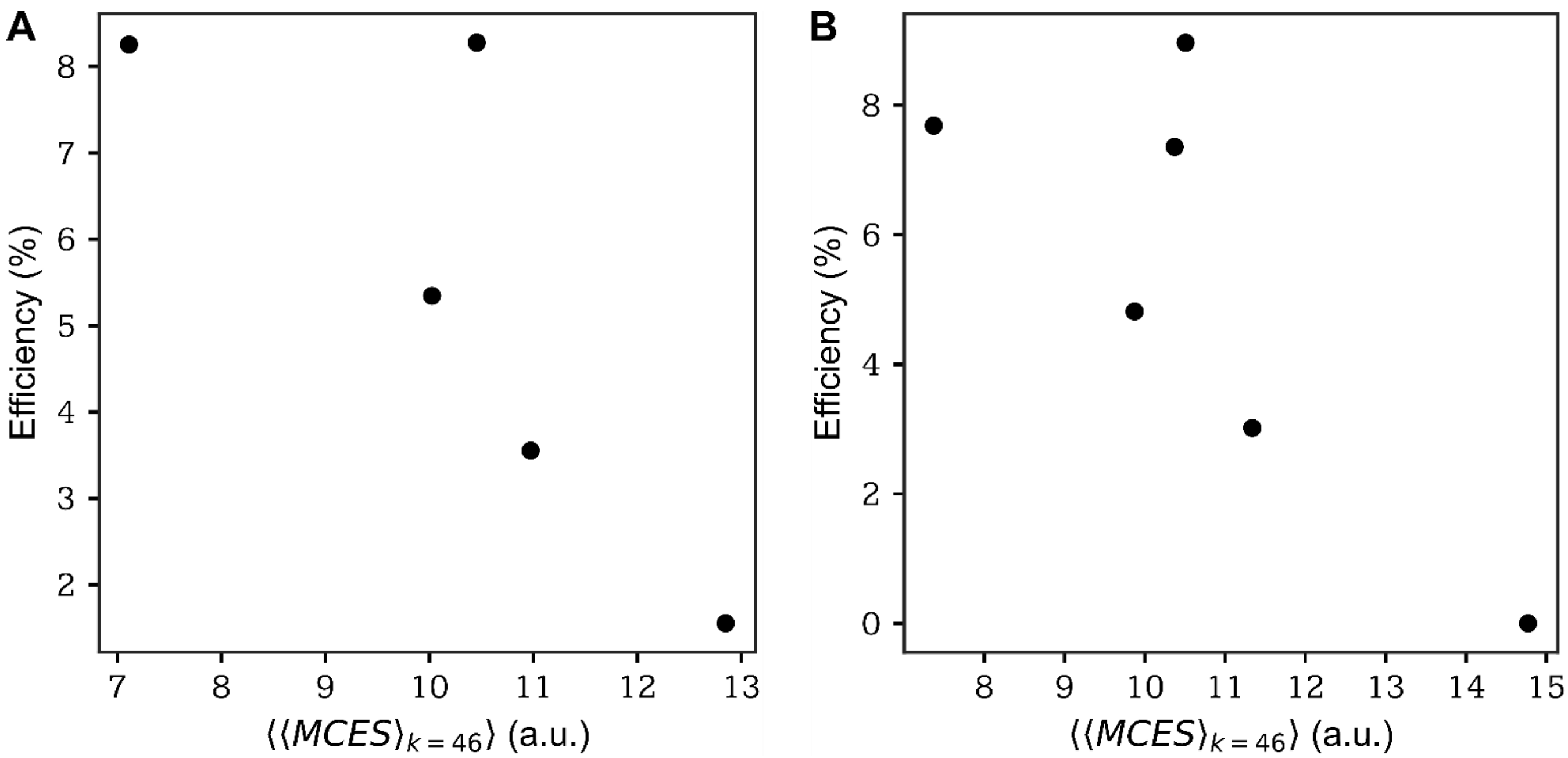

Figure S46. Efficiency (%) at $\alpha = 10\%$ in different regions of the mean distance of the candidate structures per NTS feature to the EnvCCS calibrated chemical space, denoted as $\langle\langle MCES\rangle_{k=46}\rangle$, for AllCCS_NTS (A) and the raw AllCCS_NTS (B) datasets. The *x*-axis labels correspond to the left extremes of the intervals of $\langle\langle MCES\rangle_{k=46}\rangle$, obtained with the quantile discretization explained for $\langle MCES\rangle_{k=46}$ in Methods – Evaluation Measures – Recall, in this case into bins of 100 NTS features (~970 candidates per class).

We analyze the Mondrian efficiency at $\alpha = 10\%$. Depending on $[M+H]^{+}$, $[M+Na]^{+}$ or $[M-H]^{-}$, the conformal predictive system yielded 4.8%, 8.5% and 4.8% conditional efficiencies, respectively, for AllCCS_NTS as well as 4.9%, 7.4% and 4.9% in the case of raw AllCCS_NTS.

References

(1) Kretschmer, F.; Seipp, J.; Ludwig, M.; Klau, G. W.; Böcker, S. Coverage Bias in Small Molecule Machine Learning. *Nat. Commun.* **2025**, *16* (1), 554. https://doi.org/10.1038/s41467-024-55462-w.

(2) Bates, S.; Candès, E.; Lei, L.; Romano, Y.; Sesia, M. Testing for Outliers with Conformal P-Values. *Ann. Statist.* **2023**, *51* (1), 149–178. https://doi.org/10.1214/22-AOS2244.

(3) Ash, J. R.; Wognum, C.; Rodríguez-Pérez, R.; Aldeghi, M.; Cheng, A. C.; Clevert, D.-A.; Engkvist, O.; Fang, C.; Price, D. J.; Hughes-Oliver, J. M.; Walters, W. P. Practically Significant Method Comparison Protocols for Machine Learning in Small Molecule Drug Discovery. *J. Chem. Inf. Model.* **2025**, *65* (18), 9398–9411. https://doi.org/10.1021/acs.jcim.5c01609.

(4) Angelopoulos, A. N.; Bates, S. A Gentle Introduction to Conformal Prediction and Distribution-Free Uncertainty Quantification. arXiv December 7, 2022. https://doi.org/10.48550/arXiv.2107.07511.
